# Supplementary material for: Health-promoting work schedules: protocol for a large-scale cluster randomised controlled trial on the effects of a work schedule without quick returns on sickness absence among healthcare workers
Source: BMJ Open. 2022 Apr 15;12(4):e058309. doi: 10.1136/bmjopen-2021-058309 (PMC9014074; doi:10.1136/bmjopen-2021-058309)
Supplement: Supplementary data [file bmjopen-2021-058309supp001.pdf]

Supplementary file 1

INFORMASJONSSKRIV – versjon 20.12.2020

REQUEST FOR PARTICIPATION IN QUESTIONNAIRE

This is a request if you want to participate in the project on the effect of a shift schedule without quick returns - a randomized controlled trial among health workers who work shifts at Haukeland University Hospital. The main purpose of the survey is to test whether a shift schedule without quick return will reduce sickness absence among health personnel. In addition, it will be investigated whether such shifts lead to changes in sleep and functioning, including physical and mental health, work-related accidents and turnover intention. The study is conducted by researchers at the University of Bergen, the National Institute of Public Health and Haukeland University Hospital.

WHAT DOES THE PROJECT INVOLVE?

Participation means that you answer the questionnaire that is available when logging in to minGat. You will be asked to answer the questionnaire on two occasions. The first time is now, and then you get the questionnaire again in 6 months. The questionnaire contains questions about background (gender, age, marital status, responsibility for caring for children), work and health. Data will be linked to information about shifts (quick return shifts) and sick leave taken from the payroll register 12 months before and 12 months after the intervention. Data from the payroll register contains information about your work plan and about your sick leave. For your information, the hospital will investigate the connection between working hours and sick leave in the specified period, regardless of whether you agree to participate in the survey or not (cf. EU Privacy Regulation, Article 14). This data linkage is not consent-based as it is part of the hospital's quality improvement work.

POSSIBLE BENEFITS AND DISADVANTAGES

POSSIBLE BENEFITS: Some may find it educational and interesting to participate. By participating, you also get to contribute to research and to identify more health-promoting work schedules. You will get feedback on the results by through short articles published on "Innsiden". The results can contribute to uncovering problematic conditions in healthcare workers' working conditions / situation.
POSSIBLE DISADVANTAGES: A possible disadvantage of participation may be that some may trigger some negative emotions if they have a problematic relationship with the topics we ask about.

VOLUNTARY PARTICIPATION AND OPPORTUNITY TO WITHDRAW YOUR CONSENT

It is voluntary to participate. As long as you can be identified in the data material, you have the right to: Access what personal information is registered about you, to have personal information about you corrected, to have personal information about you deleted, to receive a copy of your personal information, and to send a complaint to the Data Inspectorate about the processing of your personal data. In such cases, you can contact project manager Anette Harris (+47 55 58 32 19, anette.harris@uib.no). You can also contact the privacy ombudsman in Helse Bergen if you have questions about the health trust's processing of your personal information.

The legal basis for processing your personal data in the project is that the processing is necessary to perform a task in the public interest, and for quality improvement purposes (GDPR art. 6 (1) e) and art. 9 (2) i), and on the basis of your consent to voluntary participation in the project.

WHAT HAPPENS TO YOUR INFORMATION?

In this project, there are two types of information that are kept separate: 1) Data file with personally identifiable information (such as name, social security number and unique ID number) and 2) data file with the actual answers given and your unique ID number. The latter data is used for statistical analyzes. Only the researchers in the project

have access to these files. Data file with personally identifiable information and data file with your answers are stored separately on a secure server at UiB. All the researchers involved in the project have a statutory duty of confidentiality. When the project period is over, the file with all the personally identifiable information is deleted for good (no later than 01 January 2031). The answers you have given will then be deidentified.

APPROVAL

The Regional Committee for Medical and Health Research Ethics has made a research ethics assessment and approval of the project (2020/200386). The University of Bergen and project manager Anette Harris are responsible for the privacy of the project. An assessment of privacy conventions (DPIA) has been carried out in collaboration with the privacy ombudsman in Helse Bergen and at UiB.

CONTACT INFORMATION

If you have questions about the project, you can contact project manager Anette Harris (phone: 55 58 32 19; e-mail: anette.harris@uib.no) or work package manager Øystein Vedaa (phone: 21 07 88 34; email: oystein.vedaa@fhi.no). You can also contact us if you experience difficult feelings due to participating in the survey.
You can contact the University of Bergen's privacy representative if you have questions about the processing of your personal information in the project (Janecke Helene Veim, telephone: 55 58 20 29, email: janecke.veim@uib.no).

DO YOU WANT TO PARTICIPATE IN THIS RESEARCH PROJECT?

If you tick the box below, you give your consent to participate in this research project, and that data collected can be linked to information about your work schedule and sick leave retrieved from the payroll register, as described in this document. Tick the box below to give your consent:

☐ **I agree to participate in the research project and that data can be linked to my information from the payroll register**
