# Supplementary material for: Health-promoting work schedules: protocol for a large-scale cluster randomised controlled trial on the effects of a work schedule without quick returns on sickness absence among healthcare workers
Source: BMJ Open. 2022 Apr 15;12(4):e058309. doi: 10.1136/bmjopen-2021-058309 (PMC9014074; doi:10.1136/bmjopen-2021-058309)
Supplement: Author's manuscript [file bmjopen-2021-058309.draft_revisions.pdf]

BMJ Open

BMJ Open is committed to open peer review. As part of this commitment we make the peer review history of every article we publish publicly available.

When an article is published we post the peer reviewers' comments and the authors' responses online. We also post the versions of the paper that were used during peer review. These are the versions that the peer review comments apply to.

The versions of the paper that follow are the versions that were submitted during the peer review process. They are not the versions of record or the final published versions. They should not be cited or distributed as the published version of this manuscript.

BMJ Open is an open access journal and the full, final, typeset and author-corrected version of record of the manuscript is available on our site with no access controls, subscription charges or pay-per-view fees (http://bmjopen.bmj.com).

If you have any questions on BMJ Open's open peer review process please email info.bmjopen@bmj.com

BMJ Open

Health promoting work schedules: Protocol for a large-scale cluster randomized controlled trial on the effects of a work schedule without quick returns on sickness absence among healthcare workers

Journal:	*BMJ Open*
Manuscript ID	bmjopen-2021-058309
Article Type:	Protocol
Date Submitted by the Author:	16-Oct-2021

Complete List of Authors:	Vedaa, Oystein; Norwegian Institute of Public Health, Department of Health Promotion; Norwegian University of Science and Technology, Department of Mental Health<br>Djupedal, Ingebjørg Louise Rockwell; University of Bergen, Department of Psychosocial Science<br>Svensen, Erling; Haukeland Universitetssjukehus<br>Waage, Siri; University of Bergen, Department of Global Public Health and Primary Care<br>Bjorvatn, Bjørn; Universitetet i Bergen Det medisinsk-odontologiske fakultet, Department of Global Public Health and Primary Care; Haukeland Universitetssjukehus, Norwegian Competence Center for Sleep Disorders<br>Pallesen, Ståle; University of Bergen<br>Lie, S; University of Bergen, Department of Dentistry<br>Nielsen, Morten; University of Bergen<br>Harris, Anette; Universitetet i Bergen Det Psykologiske Fakultet

Keywords:	OCCUPATIONAL & INDUSTRIAL MEDICINE, SLEEP MEDICINE, Adult psychiatry < PSYCHIATRY

SCHOLARONE™
Manuscripts

Note from the Editors: Instructions for reviewers of study protocols

Since launching in 2011, BMJ Open has published study protocols for planned or ongoing research studies. If data collection is complete, we will not consider the manuscript.

Publishing study protocols enables researchers and funding bodies to stay up to date in their fields by providing exposure to research activity that may not otherwise be widely publicised. This can help prevent unnecessary duplication of work and will hopefully enable collaboration. Publishing protocols in full also makes available more information than is currently required by trial registries and increases transparency, making it easier for others (editors, reviewers and readers) to see and understand any deviations from the protocol that occur during the conduct of the study.

The scientific integrity and the credibility of the study data depend substantially on the study design and methodology, which is why the study protocol requires a thorough peer-review.

BMJ Open will consider for publication protocols for any study design, including observational studies and systematic reviews.

Some things to keep in mind when reviewing the study protocol:

- Protocol papers should report planned or ongoing studies. The dates of the study should be included in the manuscript.
- Unfortunately we are unable to customize the reviewer report form for study protocols. As such, some of the items (i.e., those pertaining to results) on the form should be scores as Not Applicable (N/A).
- While some baseline data can be presented, there should be no results or conclusions present in the study protocol.
- For studies that are ongoing, it is generally the case that very few changes can be made to the methodology. As such, requests for revisions are generally clarifications for the rationale or details relating to the methods. If there is a major flaw in the study that would prevent a sound interpretation of the data, we would expect the study protocol to be rejected.

For peer review only

Health promoting work schedules: Protocol for a large-scale cluster randomized controlled trial on the effects of a work schedule without quick returns on sickness absence among healthcare workers

Øystein Vedaa[1,2,3], Ingebjørg Louise Rockwell Djupedal[4], Erling Svensen[5], Siri Waage[6,7], Bjørn Bjorvatn[6,7], Ståle Pallesen[4,7,8], Stein Atle Lie[9], Morten Birkeland Nielsen[4,10], Anette Harris[4]

[1] Department of Health Promotion, Norwegian Institute of Public Health, Bergen, Norway;

[2] Department of Mental Health, Norwegian University of Science and Technology, Trondheim, Norway;

[3] Department of Research and Development, St Olavs University Hospital, Trondheim, Norway

[4] Department of Psychosocial Science, University of Bergen, Bergen, Norway;

[5] Haukeland University Hospital, Bergen, Norway;

[6] Department of Global Public Health and Primary Care, University of Bergen, Bergen, Norway;

[7] Norwegian Competence Center for Sleep Disorders, Haukeland University Hospital, Bergen, Norway;

[8] Optentia at the Vaal Triangle Campus of the North-West University, South-Africa;

[9] Department of Clinical Dentistry, University of Bergen, Bergen, Norway;

[10] National Institute of Occupational Health, Oslo, Norway

Correspondence to: Øystein Vedaa, Department of Health Promotion, Norwegian Institute of Public Health, Zander Kaaes gate 7, 5018 Bergen, Norway; e-mail: Oystein.Vedaa@fhi.no

Word count: 6064

For peer review only

Abstract

Introduction In shift work, quick returns refer to transitions between two shifts with less than 11 hours available rest time. Twenty-three per cent of employees in European countries reported having quick returns. Quick returns are related to short sleep duration, fatigue, sleepiness, work-related accidents, and sickness absence. The present study is the first randomized controlled trial (RCT) to investigate the effect of a work schedule without quick returns for six months, compared to a work schedule that maintains quick returns during the same time frame.

Methods and analysis A parallel-group cluster RCT in a target sample of about 4000 healthcare workers at Haukeland University Hospital in Norway will be conducted. More than 70 hospital units will be randomized to a work schedule without quick returns for six months or continue with a schedule that maintains quick returns. The primary outcome is objective records of sickness absence; secondary outcomes are questionnaire data ($n \approx 4000$ invited) on sleep and functioning, physical and psychological health, work-related accidents, and turnover intention. For a subsample, sleep diaries and objective sleep registrations with radar technology ($n \approx 50$) will be collected.

Ethics and dissemination The study protocol was approved by the Regional Committee for Medical and Health Research Ethics in Western Norway (2020/200386). Findings from the trial will be disseminated in peer-reviewed journals and presented at national and international conferences. Exploratory analyses of potential mediators and moderators will be reported. User-friendly outputs will be disseminated to relevant stakeholders, unions and other relevant societal groups.

Trial registration number NCT04693182; Pre-recruitment.

Key words: Quick returns, Shift work, Sickness absence, Sick leave

For peer review only

Article summary:

Strength and limitations of the study:

- This is the first randomized controlled trial to investigate the effect of a work schedule without quick returns.
- The primary outcome measure is objective register data on sickness absence with no missing data.
- As this is an evaluation of an organizational quality improvement measure implemented for all employees at the hospital, we get to study the effect on the entire target population with full representativeness.
- Two primary concerns in this trial are:
    - how well the intervention group will succeed in abolishing quick returns from the shift schedule (given that this is a study conducted in a naturalistic setting),
    - and that a shift schedule that does not include quick returns may unintentionally include other unfavourable shift characteristics that could potentially confound the results (e.g., more consecutive evening shifts

For peer review only

INTRODUCTION

An important principle when planning shift schedules is that employees are apportioned sufficient time to rest and recover between shifts. According to the EU's Working Time Directive (2003/88 / EC),[1] employees are entitled to minimum 11 hours of rest between two consecutive shifts. Still, in some countries, including Norway, employers and the employees' representatives can agree on rest periods less than 11 hours between two shifts. In this realm, the term *quick return* refers to transitions between two shifts with less than 11 hours available rest time. Quick returns occur most often between an evening shift and a day shift the following day, but can also occur between a night shift and an evening shift, and between a day shift and a night shift the subsequent night.[2] In the sixth European Working Conditions Survey published in 2016,[3] 23 per cent of employees in European countries reported having at least one quick return during the last month. Quick returns seem to be particularly prevalent in the healthcare sector. In a large Danish register survey (n = 69,200), it was shown that, on average per year, 65 per cent of nurses, 38 per cent of physicians, and 26 per cent of medical secretaries had quick returns in their work schedule.[4]

Eleven hours define the upper limit of potential time for rest between two shifts in a quick return, while the actual time available is often substantially shorter. A Norwegian study investigating payroll data from nurses found that almost 2/3 of the quick returns involved rest time less than 9 hours between two shifts, and some employees (2%) even had rest time of less than 7 hours.[5] The time available for sleep and recuperation is further curtailed by the time it takes to commute to and from work, time for self-care, meals, family obligations and house chores. A systematic literature review reported that sleep duration in quick returns between evening and day shifts typically is reduced to 5-6.5 hours, compared to 7-8 hours on non-quick return nights.[2] In addition to reduced sleep duration, the most robust findings in the literature review were that quick returns were associated with more fatigue, higher levels of

For peer review only

sleepiness, and shift work disorder (i.e., sleep problems or sleepiness related to a recurring shift schedule). Individual studies also showed that quick returns were associated with poorer sleep quality, impaired general health and well-being, higher self-reported stress, and lower job satisfaction.[2]

The most immediate consequence of quick returns is probably shortened sleep.[6] It is reasonable to think that this in turn leads to a number of other negative consequences. In a diary study (sleep- and work-schedule), we found that nurses reported higher sleepiness during the day shift when they had quick return to the day shift, as compared to during other regular day shifts.[6] In fact, the results showed that the nurses were as sleepy during the day shift after a quick return as they were during night shifts. It is conceivable that high sleepiness represents a greater problem when it occurs during day shifts than during night shifts, since day shifts are often busier[7] and typically experienced as more stressful.[6] The combination of a high level of sleepiness during a stressful shift might represent a type of circumstance that increases the risk of accidents. Indeed, the association between quick returns and work-related accidents or injuries is established in previous research. In a large register-based study from Denmark, researchers linked payroll data of healthcare workers with national registers of injuries. The results showed that quick returns were associated with a 39 per cent higher risk of injury, compared with having 15-17 hours off between two shifts.[4] A longitudinal study found an increased risk of needlestick injuries among nurses who reported having quick returns as compared to nurses without quick returns.[8] A study based on cross-sectional data found that quick returns were associated with an increased risk of falling asleep at work, of experiencing work-related injuries to themselves, of injuring patients or others, and of damaging equipment at work.[9] In fact, the risk of experiencing injuries to themselves and damaging equipment at work was greater with quick returns than with night shifts. Another longitudinal study, partly based on the same data, demonstrated that nurses who experienced

For peer review only

an increase in the number of quick returns over time also had an increased risk of work-related accidents, whereas a decrease in the number of quick returns over time was associated with reduced risk of accidents.[10]

Over the past five years, researchers have increasingly begun to use register/payroll data on exposure to shift work when examining the consequences of different shift characteristics. These data are registered by the employees, typically at healthcare institutions and include information about the date and start and stop time for all shifts performed. In some cases, it is also possible to retrieve data on sickness absence from the same registers. These data comprise information on the date of each day of absence (self-certified and medically certified absence) due to illness. In a Finnish study using such register data from healthcare workers, the relationship between quick returns and short-term sick leave (1 to 3 days) was investigated. The results showed that having few quick returns (defined as 3 or fewer over a period of 28 days) was associated with a lower risk of short-term sick leave, while having many quick returns (5 or more over a period of 28 days) was associated with a higher risk of short-term sickness absence, compared to having no quick returns.[11] In a study based on Danish and Finnish register data, it was found that healthcare workers who had at least 13 quick returns during a year had a higher risk of long-term sick leave than those with fewer quick returns.[12] These findings are in line with results using corresponding register data in Norway.[5] In one study, the findings showed that exposure to quick returns one month was associated with a higher risk of sick leave the following month. On average, nurses had 3 quick returns per month, which corresponded to 21 per cent more sickness absence days the subsequent month (over and above the sickness absence days of workers without quick return).[5]

Research on quick return and health and safety related outcomes have so far all been based on correlational studies. We do not yet know whether these health outcomes are caused by

For peer review only

exposure to quick returns. The present study is the first randomized controlled trial (RCT) conducted to determine the effects of abolishing quick return from the work schedule.

Aims

This paper describes the protocol for a two-arm cluster randomized controlled trial that assesses the consequences of a shift work schedule abolishing quick returns, compared to a schedule maintaining quick returns for a six months period. First, we will examine any differential change in sickness absence (primary outcome) during the six-month intervention period. Second, we will examine if there are differential changes in sleep and functioning, physical and mental health, work-related accidents, and turnover intention, among others (secondary outcomes). Third, we will investigate if individual characteristics associated with shift work tolerance including sex, age, personality and subjectively reported sleep need moderate the negative effects of quick returns on the primary and secondary outcomes. Finally, the study will investigate if individual factors like satisfaction with work schedule, job satisfaction, job engagement and work-family interference moderate the negative effects of quick returns on the primary and secondary outcomes.

For peer review only

METHODS AND ANALYSIS

The protocol for the current trial follows the CONSORT 2010 checklist for randomised trials. The trial is further pre-registered with the Clinical Trials website (ClinicalTrials.gov identified: NCT04693182). The CONSORT checklist for the current trial is available as online supplementary file 1.

Figure 1 shows the CONSORT Flow Diagram for the current trial. The flow chart illustrates the timeline for recruitment, randomization, assessments and for undertaking primary and secondary analyses.

Insert Figure 1 about here

Research design

A cluster randomized controlled trial comparing a six months work schedule abolishing quick returns (intervention) with that of a six months work schedule maintaining a normal amount of quick returns (control) will be conducted. The clusters in this trial represent hospital units that are randomly selected to receive (or not receive) the intervention. 'Normal amount of quick returns' refer to that which is the common practice at the respective hospital unit in recent years (i.e., when no explicit changes have been made to the work schedule), which means that the total number of quick returns at the unit will vary from 329–2356 per year (on average, nurses have three quick returns per month at this hospital[5]). In September 2020, the hospital units were informed about the conditions they would be randomized to at the start of the study in 2021. Thus, the autumn of 2020 was spent planning the shift schedule for 2021 (i.e., removing quick returns for the intervention group and maintaining quick returns for the

For peer review only

control group). Most hospital units started the intervention period in the first half of 2021, while some units started the intervention period in the second half of 2021.The intervention period in this study is six calendar months.

The primary outcome is sickness absence retrieved from the local registers kept by the hospital (including short- and long-term sick leave). The baseline measurements will be sickness absence from the year preceding the intervention, which for each individual participant will be matched on duration and season to that of the intervention period. We will apply for ethical approval to use the register data from all employees at the randomised hospital units without obtaining individual consent. In addition, a consent-based part of the trial will be conducted, in which secondary outcome measures will be collected via questionnaire at baseline and six-month follow-up. All employees ($n \approx 4000$) at the randomized units will be asked to complete a digital questionnaire. This will be made available to the employees when they log on to enter their working hours ("MinGat"). Baseline assessment will occur the month preceding the intervention period, and follow-up assessment will occur the last month of the intervention period. A subsample ($n \approx 50$) will be asked to record their sleep with advanced sleep radar technology (Somnofy™)[13] and subjectively with sleep diaries for ≥1 week at the baseline and follow-up assessments, respectively.

Participants and procedure

Recruitment

This trial is carried out in collaboration with the human resources department at Haukeland University Hospital, Bergen, Norway. All hospital care units that have 24-hour staffing at Haukeland University Hospital will be randomized. All healthcare workers working shifts will be included, except for physicians. Physicians are to be excluded since they often have a

different shift schedule and compensation scheme compared to other occupational groups at the hospital. Hereinafter, 'all employees' refer to all healthcare workers engaged in shift work at the randomised hospital units, except for physicians. All employees (n ≈ 4000) at the randomized hospital units will be asked to complete a questionnaire prior to, and at the end of, the intervention period. Recruitment for this part of the trial will take place via the hospital's internal website or through the site in which the employees enter their working hours ("MinGat"). Researchers (the authors of this paper) and human resources personnel at the hospital will attend staff meetings at all included units to inform about the research project and encourage participation. A subsample of n ≈ 50 employees (evenly distributed from the intervention and the control units) will be recruited by convenience for the objective sleep monitoring section of the trial.

Eligibility

The unit-level inclusion criteria are that the units should have 1) healthcare workers (other than physicians) who work rotating shifts, 2) employees who regularly have quick returns in their work schedule, and 3) a new shift rotation year commencing from the first half of 2021 (which is the case for most units at the included hospitals). Exclusion criteria at the unit-level are 1) units recently (or will in the near future) went through other major organizational changes that may confound the results of the trial (this includes during the period from one year before the intervention starts until the intervention period is over), or 2) unit's manager or a substantial number of employees strongly oppose participation. Haukeland University Hospital had a total of 76 units which were considered for eligibility, 67 of which were deemed eligible for the trial. **Figure 1** provides an overview of the number of units excluded before the randomization took place.

This trial consists of three different data collections with an expected dissimilar number of participants: A) a register study, i.e. the primary investigation, in which we expect no missing

For peer review only

data, B) a questionnaire study, i.e. the secondary investigation, with an expected response rate of 40-50 per cent,[14] and C) the sleep monitoring study, i.e., secondary investigation, conducted on a subsample of ≈50 employees recruited by convenience. All employees from the randomised hospital units working ≥80 percent of full-time equivalent will participate in the register-based study (investigation A) and the same group will be asked to participate in the questionnaire-based study (investigation B). Finally, participants in the sleep monitoring study (investigation C) will be recruited by convenience from the same sample of healthcare workers requiring that they are working ≥80 percent of full-time equivalent.

Randomisation

Eligible hospital units were matched in pairs according to unit types (cardiology, orthopaedic, neurology, gastroenterology, rehabilitation, psychiatry etc), and then randomized to one of the two conditions in a 1:1 ratio, according to a predetermined randomisation list generated by an online randomisation programme (www.randomization.com).

Intervention

The intervention entails implementing a shift schedule which abolishes quick returns for a six-month intervention period. The mean number of quick returns in the various hospital units in this trial varies from 3–32 per year. The intervention means that this number is abolished or reduced as much as possible. For practical reasons the intervention may be a matter of reducing rather than completely abolishing quick returns. This might be in the case of ensuring adequate staffing (e.g., due to sickness absence), and since employees for various reasons may make short-notice shift swaps in which it is not possible to comply with the rule of avoiding quick returns. The human resources department at the hospital will assist shift planners in identifying appropriate shift schedules that do not include quick returns. **Table 1** shows some of the examples that were used to show shift planners how this could be done.

For peer review only

The control condition in this trial implies that employees maintain the same number of quick returns as in previous years for the six-month intervention period. It is important to note that hospital units in the control group are not expected to experience any increase in the number of quick returns.

Insert Table 1 about here

Assessments

All assessments/instruments in this trial are described below. **Table 2** provides an overview of the source and timing of the assessments. The primary outcome in this trial is sickness absence (number of days or spells). We will compare the sickness absence in intervention group with the control group during the intervention period, while adjusting for previous sick leave from the corresponding period the year preceding the intervention (matched on duration and season). Other measures included in this trial are secondary outcomes or outcomes used in exploratory or subsidiary analyses.

Demographics

Demographic information will be obtained both from the register at the hospital as well as from a questionnaire. Information on sex, age and percentage of full-time equivalent will be available from the register data; while information on marital status, highest completed education/degree, years of experience with shift work, and if the participant has children living at home will be collected through the questionnaire.

Primary outcome

For peer review only

Sickness absence data will be retrieved from the local records kept by the hospital.[5] This record includes information about the date of any absence of the individual employee, implying that it includes information about both short- and long-term sickness absence. Further, these data include information on whether the absence is self-certified or whether it is certified by a physician, whether the absence is due to a sick child of whom the employee has childcare responsibility of, and whether the absence is due to COVID-19 related issues (e.g., quarantine).

Secondary outcomes

The Bergen Insomnia Scale (BIS)[15] will be used to measure sleep problems among participants. The scale originally comprised six items that assess symptoms of insomnia. An additional item will be included to the scale in which we will ask about the duration of any sleep problems. This makes it possible to define insomnia according to the diagnostic criteria in the International Classification of Sleep Disorders-Third Edition,[16] Diagnostic and Statistical Manual of Mental Disorders-Fifth Edition, and the International Classification of Diseases-11th Revision.

Shift work disorder (SWD) will be measured with three standardised questions.[17] SWD was evaluated with three questions based on the criteria from the third edition of the International Classification of Sleep Disorders (ICSD-3).[16] The questions were: a) Do you have a work schedule that sometimes overlap with the time you usually sleep?, b) if yes, does this cause insomnia and/or excessive sleepiness due to reduced amount of sleep?, c) if yes, has this lasted for at least three months? Participants will be classified as having SWD when responding “yes” to all three questions.

The Swedish Occupational Fatigue Inventory (SOFI) will be used to measure lack of energy, physical exertion, physical discomfort, lack of motivation and sleepiness.[18] Participants are

For peer review only

asked to indicate the extent to which they have recently (or for a specified period of time) experienced a list of 20 psychological and physical sensations related to fatigue.

The revised Circadian Type Inventory (rCTI) comprises 11 items, five of which assesses flexibility and six assesses languidity.[19] High scores on flexibility reflect better ability to sleep and work at odd times, whereas high scores on languidity indicate difficulties overcoming drowsiness and feelings of lethargy following sleep loss.

The Horne-Östberg Morningness Eveningness Questionnaire (MEQ) is the most widely used morningness-eveningness inventory,[20] and is designed to determine preferred timing of sleep and activities during the 24-hour day.[21] The MEQ reduced version (rMEQ) will be used in the present trial, which is comprised of five items from the original scale.[22]

Hopkins Symptoms Checklist - 5 (HSCL-5) will be used to measure general psychological distress.[23] HSCL-5 includes five questions about nervousness or inner turmoil, fear or feeling anxious, feeling hopeless about the future, depression or melancholy, worry or restlessness. An average score can be calculated across the five items with values that vary from 1 to 4, in which higher scores indicate a higher degree of psychological distress. The composite score is sometimes recoded into a two-part variable in which a score higher than 2.00 is defined as a high score.

Job Satisfaction Index (JSI) comprise five items measuring satisfaction with work (e.g., “I find real enjoyment in my work”).[24] Each item is answered on a 5-point Likert scale, ranging from 1 (strongly disagree) to 5 (strongly agree). Higher scores reflect higher levels of overall job satisfaction.

The Work Family Interface Scale[25] will be used to evaluate the four types of work–family spillover. Consisting of 14 items, the scale was designed to measure both negative and positive work–to– family (NWFS and PWFS) and family–to–work spillover (NFWS and

For peer review only

PFWS). The responses were graded by a frequency based on a 1–5 Likert scale, with alternatives ranging from never to very often.

Work-related negative incidents will be assessed using eight items measuring the number of self-reported work-related accidents, near accidents and dozing off at work or while driving to or from work. These questions have been developed in connection with the Norwegian Survey of Shift work, Sleep and Health among Nurses (SUSSH), and have been used in several previous publications.[26]

The Turnover Intention Scale (TIS) will be used to measure turnover intention, which is comprised of three items adapted from Michigan Organizational Assessment Questionnaire.[27] The three items are: "I will actively look for a new job in the next year," "I often think about quitting," and "I will probably look for a new job by the next year." Responses were recorded on a 5-point Likert scale from 1 (strongly disagree) to 5 (strongly agree), yielding a score range of 3–15. A high score indicates a high degree of turnover intention.

The Utrecht Work Engagement Scale (UWES - 9) will be used to measure work engagement.[28] The UWES is originally comprised of 17 items rated on a 7-point scale ranging from "never" (0) to "always/every day" (6). The 9-item version of the UWES includes three items for each of the three factors; Vigor (e.g., "At my job, I feel strong and vigorous"), Dedication (e.g., "I am enthusiastic about my job"), and Absorption (e.g., "When I am working, I forget everything else around me"). A higher score indicates more work engagement.

Subjective Health Complaints inventory (SHC)[29] consists of a list of 29 common health complaints that participants grade the intensity of which they experience each complaint on a four-point scale (0 = not at all; 1 = a little; 2 = some; 3 = severe). In this study, we include

For peer review only

three of the five subscales; i.e. musculoskeletal complaints, pseudoneurological complaints, and gastrointestinal complaints.

Recovery Experience Questionnaire (REQ)[30] will be used to measure recovery experiences. REQ is originally a 16-item questionnaire with the four subscales psychological detachment, relaxation, mastery, and control. The present study includes the subscales of psychological detachment and relaxation. Each item is scored on a 5-point Likert scale ranging from 1 (strongly disagree) to 5 (strongly agree) of which a higher score indicates better detachment/relaxation.

Epworth Sleepiness Scale (ESS)[31] will be used to measure participants sleepiness. ESS is an eight-item questionnaire asking the participants how likely they are to doze off or fall asleep in different situations of everyday life including (e.g. while sitting and reading, watching TV, when sitting and talking to someone, etc.). For each item, participants report the chance of dozing as never (0), slight (1), moderate (2), or high (3) (total score range between 0 – 24). A higher score indicates higher level of sleepiness.

Additional measures of unwanted/negative effects and other exploratory analyses

Other factors that may have an impact on how the employees react to the intervention will also be investigated. The participants' attitudes to the intervention and the research project will be measured, in addition to how they experience the implementation of the intervention. A set of questions measuring possible negative or unwanted effects of the intervention will be developed for the purpose of this trial. These questions will specifically ask if the changed work schedule has led to disturbed sleep, more stress, worry, depression, overall less time for recovery between work periods, problems in work-family balance, disrupted social relationships, poorer psychosocial climate at work, experience of reduced quality of care offered to patients, etc. For some employees, it is possible that a work schedule that does not

For peer review only

allow for quick returns represents a restricted opportunity to co-design their schedule (i.e., self-rostering) and reduces the duration of free periods. Therefore, we will measure the participants' perceived change in relation to these parameters. Furthermore, we will include questions about satisfaction with work schedule, commute time, habitual and preferred sleep duration, current use of prescribed or over-the-counter sleep medication, current use of light treatment to improve sleep, and participants' physical activity level. Finally, the questionnaire will include an open text box in which participants can write freely, for example about anything they would like to convey related to the intervention (e.g. topics/themes they felt was inadequately addressed in the survey).

Sleep will be assessed more thoroughly for a subsample of ≈50 employees. The measures of sleep will include daily self-rating of sleep-wake patterns reported using the consensus sleep diary,[17] as well as sleep measured objectively using the Xethru sensor, a low-powered ultra-wideband radar.[32] The sleep registration will occur for ≥7 days at baseline and at six-month follow-up.

Sample size

The necessary sample size (assuming a power of .80 and significance level of .05) was calculated to be 448 participants in each condition. This calculation was based on the mean values of sickness absence days per month (0.9 days, SD=1.6), as reported in Vedaa et al (2016).[5] In the present trial, we will also examine potential moderators and mediators in exploratory analyses, which will require an appreciably larger sample size. For the primary outcome measure in this trial (sickness absence from register data), we will have an expected sample size of ≈ 4000. This includes all employees in the randomized hospital units and is considered sufficient for all conceivable purposes of this trial.

For peer review only - http://bmjopen.bmj.com/site/about/guidelines.xhtml

Data analysis plan

All analyses will be conducted based on the intention-to-treat population, unless otherwise stated. To examine the effects of a shift schedule abated of quick returns on primary and secondary outcomes, the observed rates or scores will be analysed by means of latent growth models (or other equivalent models such as generalized linear mixed models). The observed rates or scores before and during the intervention period will be modelled by a random intercept and a fixed slope. The effect of the intervention will be estimated by using the group variable (intervention vs. control) as a predictor of the slope. Between-group effect sizes (Cohen's d) will be calculated by dividing the mean difference in estimated change in scores from baseline to the follow-up assessment by the pooled SD at baseline. Robust maximum likelihood will be used as the estimator, providing unbiased estimates under the assumption of data being missing at random,[33] which might be partly met through the inclusion of baseline scores to the model. The primary outcome measure in this trial is sickness absence data retrieved from the register at the hospital, in which we expect no missing data. However, it is reasonable to expect some missing data on the secondary outcome measures, as data are collected through questionnaire or via the sleep radar and sleep diary.

As some data for the follow-up questionnaire and sleep radar/diary assessment will be missing not at random, the robustness of the results under the missing-at-random assumption will be tested by sensitivity analyses in which the missing scores at follow-up will be replaced by baseline values for each respective individual. Since it is possible to imagine that some participants may experience worsening because of the intervention, we will consider carrying out more rigorous sensitivity analyses. For example, by replacing missing scores at the follow-up assessment with baseline scores multiplied by a given factor (higher or lower than 1.00 depending on the direction that indicates a worsening) in the intervention group and by

For peer review only

1.00 in the control group. These sensitivity analyses will only be performed on selected variables depending on the focus in the respective article.

The intention-to-treat analyses may be accompanied by selected per-protocol analyses in which we, based on payroll data, define a group that has completely abolished or had a satisfactory reduction in the number of quick returns during the intervention period.

The primary outcome of sick leave will mainly be analysed in terms of the total number of sickness absence days and periods (spells) for a given period *before* compared to *during* the intervention period.[5] The models of sickness absence will take into account the zero inflation in this type of data. Other operationalisations of sickness absence might also be considered in accordance with recommendations in the literature.[34] For a further investigation of the sickness absence data, we will consider the use of more complex survival analyses (e.g., Cox proportional hazards model), and we will also consider modelling time to return to work (from sickness absence) and/or time before taking sickness absence according to group allocation.

Since the introduction of a work schedule without quick returns may entail an alternative schedule with an increase in other undesirable characteristics (e.g., more consecutive evening shifts), we will consider conducting analyses that adjust for such characteristics.

Mediator and moderator analyses will be performed for exploratory purposes, based on the basic principle for such analyses in randomised controlled trials as described by others (e.g., [35]). For example, some of the data collected on demographics, sleep-related personality traits (rCTI and MEQ), mental health, among others, can be used to examine factors that may moderate the impact of the intervention.

Ethics and dissemination

For peer review only

For peer review only - http://bmjopen.bmj.com/site/about/guidelines.xhtml

The study protocol was approved by the Regional Committee for Medical and Health Research Ethics in Western Norway (2020/200386). In this trial, all employees at the included hospital units will be randomized to one of two conditions, and we will retrieve register data on working hours and sickness absence without collecting individual consent. This poses an ethical dilemma since all participation in research – especially when people are exposed to an intervention – should be consent-based. However, the intervention in this trial is to abolish or substantially reduce quick returns, and *not* to increase any exposure. This is thus considered not to represent a significant burden on the participants, as the presence of quick returns is already a violation of the Working Environment Act. In addition, we expect that the intervention primarily will have beneficial effects on employees' health and safety. Abolishing or reducing the number of quick returns is a quality improvement measure that the Health Trust wants to implement independently of the present research project. The fact that the intervention is carried out as a research project is considered an advantage for the employees, as far as we are able to uncover any unintended negative effects of the intervention and further to be able to empirically document potential benefits on health and safety.

The result of this trial will potentially impact subsequent standards and practice when it comes to planning shift schedules and their compliance with the Working Environment Act. As vast number of employees might be affected by the trial results, it is equally important that the results are representative of the employees. We believe this justifies the use of the employees' register data without obtaining individual consent.

Participants will be required to provide informed consent before participating in the questionnaire and sleep diary/radar part of the trial. The recruitment and consent process emphasizes that participation is voluntary and that participants can withdraw from this part of the trial at any time point without any consequences. Self-report data are recorded in

For peer review only

electronic files that are encrypted and password protected. No identifying information will be stored alongside the self-report data. Furthermore, only researchers directly involved in data analysis will be granted supervised access to de-identified participant data.

Findings from this randomized controlled trial will be disseminated in peer-reviewed publications and as conference presentations. After the research project is completed, Haukeland University Hospital will arrange a conference for stakeholders where the results and experience from the research will be disseminated and discussed.

Stakeholder and public involvement

This trial is carried out in close collaboration with the HR department at Haukeland University Hospital. In addition, representatives from all relevant unions at the hospital will be involved in the planning and implementation of the research project. The findings of the trial will be disseminated via academics, and by stakeholder/union advocacy and other relevant public and community groups.

Patient involvement

No patient involved.

DISCUSSION

To the best of our knowledge, this is the first randomized controlled trial to investigate the effect of a work schedule abolishing quick returns. Previous research on quick returns has been dominated by cross-sectional studies and a few longitudinal investigations. Although quick returns have consistently been associated with negative health and safety outcomes, it is unclear whether quick returns are the cause of these negative outcomes. This trial will thus be the first sincere attempt to establishing such a causal relationship.

For peer review only

There are several major strengths to this trial. The intervention is carried out in all eligible hospital units at Haukeland University Hospital, in which we retrieve objective register data (notably with no missing data) on the primary outcome measure – sickness absence. Hence reporting bias such as social desirability and memory biases will be avoided. This study is unique as it will imply complete access to the entire target population, also including individuals who typically choose not to participate in such studies. Hence this ensures full representativeness, strengthening the external validity of the study. Further, we have access to objective data on exposure to shift work (quick returns and other shift characteristics) during the intervention period. This provides us the opportunity to accurately assess compliance with the intervention and the true reduction in quick returns that occur, as well as monitoring other systemic differences that might occur in the shift schedule between the two parallel conditions. It is also an asset that we combine objective data with data collected via questionnaire. This provides us the opportunity to study the effect of abolishing or reducing quick returns on sleep, health and safety, as well as being able, for example, to study potential moderators to any effects we observe.

There are also some possible limitations with this trial that should be mentioned. The trial is conducted in a naturalistic setting which does not allow for the same strict control as generally would be preferred in experimental designs. One main concern is how well the intervention group will succeed in abolishing quick returns from the shift schedule. We expect that for many individuals it will be a matter of reducing the number of quick returns, rather than complete abolition, for example, since such shift transitions occasionally may be necessary to ensure adequate staffing. Another concern is that a shift schedule that does not include quick returns may unintentionally include other unfavourable shift characteristics that could potentially confound the results. However, during the implementation of the trial, shift planners are provided with recommendations on how to set up shift schedules without quick

For peer review only

returns, e.g. avoiding backward shift rotations, which as far as possible avoids other unfavourable shift characteristics. Further, for the participants in this trial it will be obvious which study condition they have been allocated to, thus their expectations can potentially have an impact on results based on self-reported data.[36]

If a shift schedule without quick returns is shown to be associated with less sickness absence or positive effects on other outcomes compared to a control group, this may encourage a stricter compliance with the workers' right to have at least 11 hours off between two subsequent shifts. The results of this trial will provide valuable information to stakeholders (nurses responsible for developing shift schedules, trade unions, politicians, and innovators) about the effect of quick returns and individual tolerance to quick returns.

For peer review only

Author statement: AH, ØV, SP, BB, SW, SAL, ES, and MBN conceived the study. ØV and ILRD produced the first draft of the manuscript. All authors assisted in drafting of the final, submitted version of manuscript and all authors have approved this version.

Conflict of Interest: The authors declare that they have no conflict of interest.

Funding: The study was funded from The Research Council of Norway (303671) and the University of Bergen, Bergen, Norway.

Data statement: De-identified data that underlie the results reported from the trial described in this protocol will be available to researchers from accredited research institutions. Access to data will be limited to investigators who provide a methodologically sound proposal and will be limited to a specified time period (commencing about 3 months after publication of a respective Article and ending after 5 years). To ensure compliance with the General Data Protection Regulation, data processing must be covered by the European Commission's standard contractual clauses for the transfer of data, which must be signed by the data requesters. Proposals and requests for data access should be directed to the corresponding author of the respective Article. User-friendly output from the trial will be disseminated to stakeholder and other relevant organisations.

Acknowledgments: We would like to thank Ljiljana Djuric-Rakovic and John Olav Larssen at Haukeland University Hospital for their invaluable help in setting up and distributing the electronic questionnaires for this study. We would also like to thank Helga Berdal Lorentzen and Ole-Daniel Tuft Virkesdal at the HR department at Haukeland University Hospital, and employee representatives of the Norwegian Nurses Organisation, Trade Union Delta, the joint organization for Child Welfare Educators, Social Workers and Learning Disability Nurse and others trade unions for their support and contribution in the implementation of this research project. We would also like to thank Lukas Krondorf at Vital Things AS for technical support during the registration of nurses' sleep using radar technology.

For peer review only

References

1. EU Directive. Directive 2003/88/EC of the European Parliament and of the Council of 4 November 2003 concerning certain aspects of the organisation of working time. In: European Parliament, ed., 2003.
2. Vedaa Ø, Harris A, Bjorvatn B, et al. Systematic review of the relationship between quick returns in rotating shift work and health-related outcomes. *Ergonomics* 2016;59:1-14.
3. European Foundation for the Improvement of Living and Working Conditions. Sixth European working conditions survey–overview report. Luxembourg: Publications Office of the European Union., 2016.
4. Nielsen HB, Hansen ÅM, Conway SH, et al. Short time between shifts and risk of injury among Danish hospital workers: a register-based cohort study. *Scandinavian Journal of Work, Environment & Health* 2018 doi: 10.5271/sjweh.3770
5. Vedaa Ø, Pallesen S, Waage S, et al. Short rest between shift intervals increases the risk of sick leave: a prospective registry study. *Occupational and Environmental Medicine* 2017;74:496-501.
6. Vedaa Ø, Mørland E, Larsen M, et al. Sleep detriments associated with quick returns in rotating shift work: A diary study. *Journal of Occupational and Environmental Medicine* 2017;59:522-27.
7. Nicoletti C, Spengler CM, Läubli T. Physical workload, trapezius muscle activity, and neck pain in nurses' night and day shifts: a physiological evaluation. *Applied ergonomics* 2014;45:741-46.
8. Trinkoff AM, Le R, Geiger-Brown J, et al. Work schedule, needle use, and needlestick injuries among registered nurses. *Infection Control and Hospital Epidemiology* 2007;28:156-64. doi: 10.1086/510785
9. Vedaa Ø, Harris A, Erevik E, et al. Short rest between shifts (quick returns) and night work are associated with work related accidents. *International Archives of Occupational and Environmental Health* 2019;92:829-35. doi: doi.org/10.1007/s00420-019-01421-8
10. Vedaa Ø, Harris A, Waage S, et al. A longitudinal study on the association between quick returns and occupational accidents. *Scandinavian Journal of Work, Environment & Health* 2020 doi: 10.5271/sjweh.3906
11. Ropponen A, Koskinen A, Puttonen S, et al. Exposure to working-hour characteristics and short sickness absence in hospital workers: A case-crossover study using objective data. *Int J Nurs Stud* 2019;91:14-21.
12. Larsen AD, Ropponen A, Hansen J, et al. Working time characteristics and long-term sickness absence: a large register-based study of Danish and Finnish nurses. *Int J Nurs Stud* 2020 doi: https://doi.org/10.1016/j.ijnurstu.2020.103639
13. Toften S, Pallesen S, Hrozanova M, et al. Validation of sleep stage classification using non-contact radar technology and machine learning (Somnofy®). *Sleep Medicine* 2020;75:54-61.
14. Baruch Y, Holtom BC. Survey response rate levels and trends in organizational research. *Human Relations* 2008;61:1139-60.
15. Pallesen S, Bjorvatn B, Nordhus IH, et al. A new scale for measuring insomnia: the Bergen Insomnia Scale. *Percept Mot Skills* 2008;107:691-706.
16. American Academy of Sleep M. International classification of sleep disorders, 3rd ed. 3rd ed. ed: Darien, IL: American Academy of Sleep Medicine 2014.
17. Waage S, Moen BE, Pallesen S, et al. Shift work disorder among oil rig workers in the North Sea. *Sleep* 2009;32(4):558-65. [published Online First: 2009/05/06]
18. Ahsberg E, Gamberale F, Gustafsson K. Perceived fatigue after mental work: an experimental evaluation of a fatigue inventory. *Ergonomics* 2000;43(2):252-68. doi: Doi 10.1080/001401300184594
19. Di Milia L, Smith PA, Folkard S. A validation of the revised circadian type inventory in a working sample. *Personality and Individual Differences* 2005;39:1293-305.

For peer review only

20. Adan A, Archer SN, Hidalgo MP, et al. Circadian Typology: A Comprehensive Review. *Chronobiology International, 2012, Vol29(9), p1153-1175* 2012;29(9):1153-75. doi: 10.3109/07420528.2012.719971
21. Horne JA, Ostberg O. A self-assessment questionnaire to determine morningness-eveningness in human circadian rhythms. *International journal of chronobiology* 1976;4(2):97-110. [published Online First: 1976/01/01]
22. Adan A, Almirall H. Horne & Östberg morningness-eveningness questionnaire: A reduced scale. *Personality and Individual Differences* 1991;12(3):241-53. doi: https://doi.org/10.1016/0191-8869(91)90110-W
23. Schmalbach B, Zenger M, Tibubos AN, et al. Psychometric properties of two brief versions of the Hopkins Symptom Checklist: HSCL-5 and HSCL-10. *Assessment* 2021;28:617-31.
24. Brayfield AH, Rothe HF. An index of job satisfaction. *Journal of applied psychology* 1951;35:307.
25. Kinnunen U, Feldt T, Geurts S, et al. Types of work-family interface: Well-being correlates of negative and positive spillover between work and family. *Scandinavian Journal of Psychology* 2006;47:149-62.
26. Vedaa O, Harris A, Erevik EK, et al. Short rest between shifts (quick returns) and night work is associated with work-related accidents. *Int Arch Occup Environ Health* 2019 doi: 10.1007/s00420-019-01421-8 [published Online First: 2019/03/18]
27. Bowling NA, Hammond GD. A meta-analytic examination of the construct validity of the Michigan Organizational Assessment Questionnaire Job Satisfaction Subscale. *Journal of Vocational Behavior* 2008;73:63-77.
28. Schaufeli WB, Salanova M, González-Romá V, et al. The measurement of engagement and burnout: a confirmative analytic approach. *Journal of Happiness Studies* 2002;3:71– 92.
29. Eriksen HR, Ihlebæk C, Ursin H. A scoring system for subjective health complaints (SHC). *Scandinavian Journal of Public Health* 1999;27:63-72.
30. Sonnentag S, Fritz C. The Recovery Experience Questionnaire: development and validation of a measure for assessing recuperation and unwinding from work. *Journal of Occupational Health Psychology* 2007;12:204-21.
31. Johns MW. A new method for measuring daytime sleepiness: the Epworth sleepiness scale. *Sleep* 1991;14:540-45.
32. Pallesen S, Grønli J, Myhre K, et al. A pilot study of impulse radio ultra wideband radar technology as a new tool for sleep assessment. *Journal of Clinical Sleep Medicine* 2018;14:1249-54.
33. Enders CK. Applied missing data analysis. New York, NY, US: Guilford Press 2010.
34. Hensing G, Alexanderson K, Allebeck P, et al. How to measure sickness absence? Literature review and suggestion of five basic measures. *Scandinavian Journal of Public Health* 1998;26:133-44.
35. Kraemer HC, Wilson GT, Fairburn CG, et al. Mediators and moderators of treatment effects in randomized clinical trials. *Archives of General Psychiatry* 2002;59:877-83.
36. Orne MT. On the social psychology of the psychological experiment: With particular reference to demand characteristics and their implications. *American Psychologist* 1962;17:776–83.

For peer review only

Table 1. Examples of a two-week cycle of rotating shift work with and without quick returns

	Week 1							Week 2						
	Monday	Tuesday	Wednesday	Thursday	Friday	Saturday	Sunday	Monday	Tuesday	Wednesday	Thursday	Friday	Saturday	Sunday
Scenario1: Rotating three-shift **with** quick returns	Day	Day	Night	Night				Evening	Day	Day		Evening	Day	Evening
Scenario1: Rotating three-shift **without** quick returns	Day	Day	Night	Night				Day	Day	Day		Evening	Evening	Evening
Scenario2: Rotating three-shift **with** quick returns	Evening	Day	Day		Night	Night	Night			Evening	Day	Day		
Scenario2: Rotating three-shift **without** quick returns	Day	Evening	Evening		Night	Night	Night			Day	Day	Day		
Scenario3: Weekend shift **with** quick returns	Evening	Day	Day		Evening	Day	Evening	Day		Day	Day			
Scenario3: Weekend shift **without** quick returns	Day	Day	Day		Day	Evening	Evening		Day	Day	Evening			
Scenario4: Rotating two-shift **with** quick returns		Day	Day	Evening	Day			Evening	Day	Evening		Day		
Scenario4: Rotating two-shift **without** quick returns	Evening		Day	Day	Day			Evening	Evening		Day	Day		

Note. Rotating three-shift refers to a shift schedule in which the workers alternates between day-, evening- and night shifts. Rotating two-shift refers to a shift schedule in which the workers alternates between only two of the shifts (e.g., only working day and evening shifts).

For peer review only

Table 2. Key measures and timing of assessment

	Baseline	Six-month follow-up
**Primary outcome**		
From hospital register		
Sickness absence	X	X
**Secondary outcomes**		
Self-reported questionnaires		
The Bergen Insomnia Scale (BIS)	X	X
Shift work disorder (SWD)	X	X
The Swedish Occupational Fatigue Inventory (SOFI)	X	X
The revised Circadian Type Inventory (rCTI)	X	
The Horne-Östberg Morningness Eveningness Questionnaire (MEQ)	X	
The Hopkins Symptom Checklist - 5 (HSCL- 5)	X	X
Job Satisfaction Index (JSI)	X	X
The Work-Family Interface Scale (WFIS)	X	X
Work-related negative incidents	X	X
The Turnover Intention Scale (TIS)	X	X
The Utrecht Work Engagement Scale (UWES - 9)	X	X
Subjective Health Complaints inventory (SHC) (three of five subscales)	X	X
Recovery Experience Questionnaire (REQ) (two of four dimensions)	X	X
Epworth Sleepiness Scale (ESS)	X	X
Sleep monitoring study (≈50)		
Sleep diary (≥7 days)	X	X
Xethru sensor (≥7 days)	X	X
**Additional measures**		
Self-reported questionnaires		
Unwanted/negative effects		X
Self-rostering	X	X
Experience of the implementation of the intervention		X
Physical activity	X	X
Commute time	X	
Sleep duration and perceived need for sleep	X	X
Use of sleep medication and light treatment	X	X
Satisfaction with work schedule	X	X
Preferred presence of quick return in work schedule	X	X
**Demographics and background information**		
From hospital register		
Sex	X	
Age	X	
Percentage of full-time equivalent	X	X
Payroll data	X	X
Self-reported questionnaires		

For peer review only

2[illegible]

Marital status	X
Highest completed degree	X
Years of experience with shift work	X
Children living at home	X

For peer review only

Figure caption

Figure 1. CONSORT Flow Diagram of timeline for recruitment, randomization, assessments and for undertaking primary and secondary analyses

For peer review only

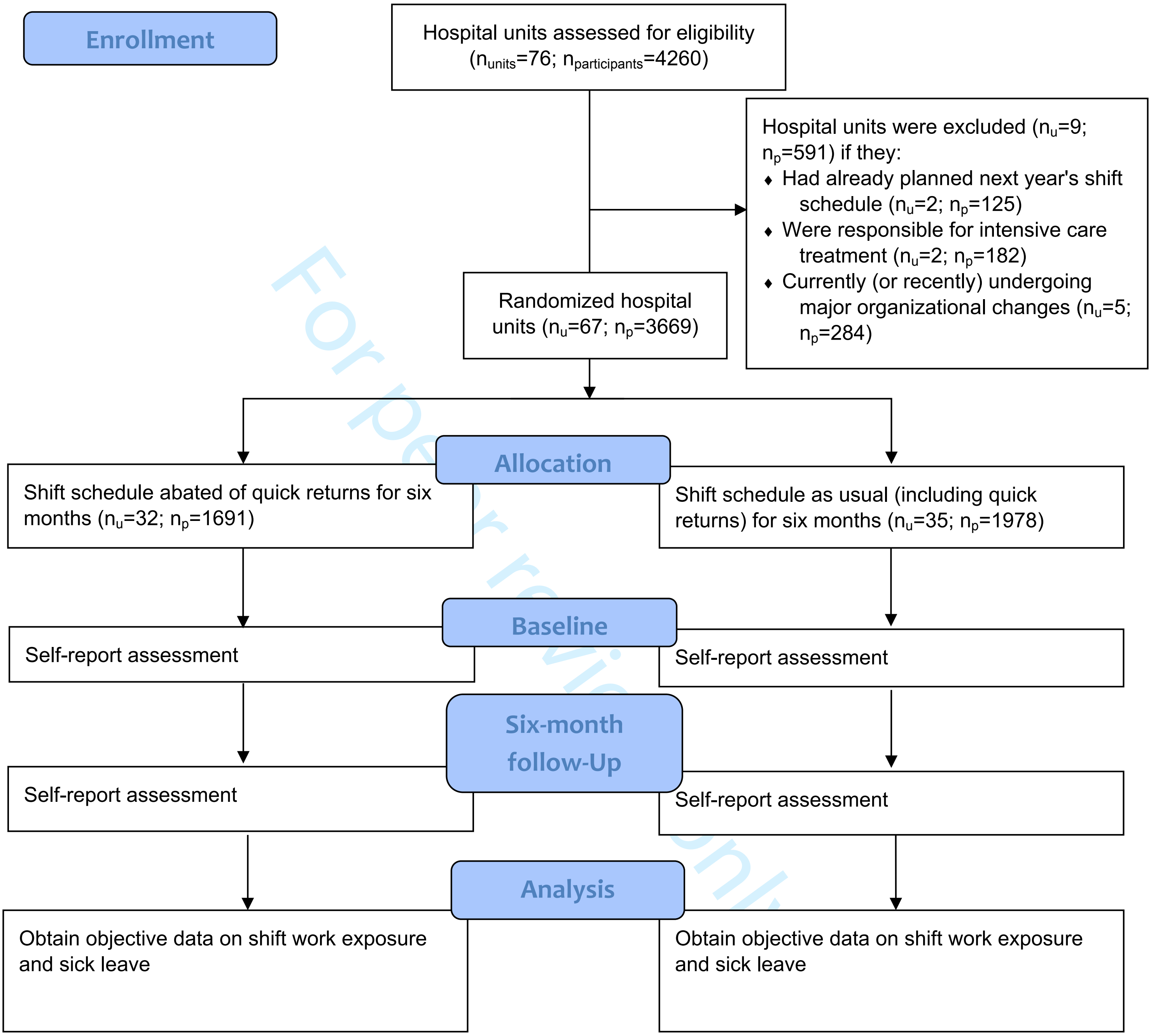

Figure 1. CONSORT Flow Diagram of timeline for recruitment, randomization, assessments and for undertaking primary and secondary analyses

Table 1: CONSORT 2010 checklist of information to include when reporting a cluster randomised trial

Section/Topic	Item No	Standard Checklist item	Extension for cluster designs	Page No *
**Title and abstract**				
	1a	Identification as a randomised trial in the title	Identification as a cluster randomised trial in the title	1
	1b	Structured summary of trial design, methods, results, and conclusions (for specific guidance see CONSORT for abstracts)[1,2]	See table 2	2
**Introduction**				
**Background and objectives**	2a	Scientific background and explanation of rationale	Rationale for using a cluster design	3-6
	2b	Specific objectives or hypotheses	Whether objectives pertain to the cluster level, the individual participant level or both	6
**Methods**				
**Trial design**	3a	Description of trial design (such as parallel, factorial) including allocation ratio	Definition of cluster and description of how the design features apply to the clusters	7-8
	3b	Important changes to methods after trial commencement (such as eligibility criteria), with reasons		**NA**
**Participants**	4a	Eligibility criteria for participants	Eligibility criteria for clusters	9-10
	4b	Settings and locations where the data were collected		**8-9**
**Interventions**	5	The interventions for each group with sufficient details to allow replication, including how and when they were actually administered	Whether interventions pertain to the cluster level, the individual participant level or both	10-11
**Outcomes**	6a	Completely defined pre-specified primary and	Whether outcome measures pertain to the cluster level, the	11-16

		secondary outcome measures, including how and when they were assessed	individual participant level or both	
	6b	Any changes to trial outcomes after the trial commenced, with reasons		
**Sample size**	7a	How sample size was determined	Method of calculation, number of clusters(s) (and whether equal or unequal cluster sizes are assumed), cluster size, a coefficient of intracluster correlation (ICC or *k*), and an indication of its uncertainty	16-17
	7b	When applicable, explanation of any interim analyses and stopping guidelines		
**Randomisation:**				
**Sequence generation**	8a	Method used to generate the random allocation sequence		**10**
	8b	Type of randomisation; details of any restriction (such as blocking and block size)	Details of stratification or matching if used	10
**Allocation concealment mechanism**	9	Mechanism used to implement the random allocation sequence (such as sequentially numbered containers), describing any steps taken to conceal the sequence until interventions were assigned	Specification that allocation was based on clusters rather than individuals and whether allocation concealment (if any) was at the cluster level, the individual participant level or both	10
**Implementation**	10	Who generated the random allocation sequence, who enrolled participants, and who assigned participants to interventions	Replace by 10a, 10b and 10c	8-10
	10a		Who generated the random allocation sequence, who enrolled clusters, and who assigned clusters to interventions	8-10

For peer review only - http://bmjopen.bmj.com/site/about/guidelines.xhtml

	10b		Mechanism by which individual participants were included in clusters for the purposes of the trial (such as complete enumeration, random sampling)	8-10
	10c		From whom consent was sought (representatives of the cluster, or individual cluster members, or both), and whether consent was sought before or after randomisation	8-10
**Blinding**	11a	If done, who was blinded after assignment to interventions (for example, participants, care providers, those assessing outcomes) and how		**NA**
	11b	If relevant, description of the similarity of interventions		**10-11**
**Statistical methods**	12a	Statistical methods used to compare groups for primary and secondary outcomes	How clustering was taken into account	17-18
	12b	Methods for additional analyses, such as subgroup analyses and adjusted analyses		**17-18**
**Results**				
**Participant flow (a diagram is strongly recommended)**	13a	For each group, the numbers of participants who were randomly assigned, received intended treatment, and were analysed for the primary outcome	For each group, the numbers of clusters that were randomly assigned, received intended treatment, and were analysed for the primary outcome	Figure 1
	13b	For each group, losses and exclusions after randomisation, together with reasons	For each group, losses and exclusions for both clusters and individual cluster members	Figure 1

For peer review only

**Recruitment**	14a	Dates defining the periods of recruitment and follow-up		**To be reported**
	14b	Why the trial ended or was stopped		
**Baseline data**	15	A table showing baseline demographic and clinical characteristics for each group	Baseline characteristics for the individual and cluster levels as applicable for each group	**To be reported**
**Numbers analysed**	16	For each group, number of participants (denominator) included in each analysis and whether the analysis was by original assigned groups	For each group, number of clusters included in each analysis	**To be reported**
**Outcomes and estimation**	17a	For each primary and secondary outcome, results for each group, and the estimated effect size and its precision (such as 95% confidence interval)	Results at the individual or cluster level as applicable and a coefficient of intracluster correlation (ICC or k) for each primary outcome	**To be reported**
	17b	For binary outcomes, presentation of both absolute and relative effect sizes is recommended		
**Ancillary analyses**	18	Results of any other analyses performed, including subgroup analyses and adjusted analyses, distinguishing pre-specified from exploratory		
**Harms**	19	All important harms or unintended effects in each group (for specific guidance see CONSORT for harms[3])		**To be reported**
**Discussion**				20-22
**Limitations**	20	Trial limitations, addressing sources of potential bias, imprecision, and, if relevant, multiplicity of analyses		20-22
**Generalisability**	21	Generalisability (external validity, applicability) of the trial findings	Generalisability to clusters and/or individual participants (as relevant)	20-22

**Interpretation**	22	Interpretation consistent with results, balancing benefits and harms, and considering other relevant evidence	**To be reported (this is just the protocol)**
**Other information**			
**Registration**	23	Registration number and name of trial registry	**2 and 7**
**Protocol**	24	Where the full trial protocol can be accessed, if available	**This is the protocol**
**Funding**	25	Sources of funding and other support (such as supply of drugs), role of funders	**1**

* *Note: page numbers optional depending on journal requirements*

For peer review only

Table 2: Extension of CONSORT for abstracts[1,2] to reports of cluster randomised trials

Item	Standard Checklist item	Extension for cluster trials
**Title**	Identification of study as randomised	**Identification of study as cluster randomised**
**Trial design**	Description of the trial design (e.g. parallel, cluster, non-inferiority)	
**Methods**		
**Participants**	Eligibility criteria for participants and the settings where the data were collected	**Eligibility criteria for clusters**
**Interventions**	Interventions intended for each group	
**Objective**	Specific objective or hypothesis	**Whether objective or hypothesis pertains to the cluster level, the individual participant level or both**
**Outcome**	Clearly defined primary outcome for this report	**Whether the primary outcome pertains to the cluster level, the individual participant level or both**
**Randomization**	How participants were allocated to interventions	**How clusters were allocated to interventions**
**Blinding (masking)**	Whether or not participants, care givers, and those assessing the outcomes were blinded to group assignment	
**Results**		
**Numbers randomized**	Number of participants randomized to each group	**Number of clusters randomized to each group**
**Recruitment**	Trial status[1]	
**Numbers analysed**	Number of participants analysed in each group	**Number of clusters analysed in each group**
**Outcome**	For the primary outcome, a result for each group and the estimated effect size and its precision	**Results at the cluster or individual participant level as applicable for each primary outcome**
**Harms**	Important adverse events or side effects	
**Conclusions**	General interpretation of the results	
**Trial registration**	Registration number and name of trial register	
**Funding**	Source of funding	

[1] Relevant to Conference Abstracts

REFERENCES

1 Hopewell S, Clarke M, Moher D, Wager E, Middleton P, Altman DG, et al. CONSORT for reporting randomised trials in journal and conference abstracts. *Lancet* 2008, 371:281-283

2 Hopewell S, Clarke M, Moher D, Wager E, Middleton P, Altman DG at al (2008) CONSORT for reporting randomized controlled trials in journal and conference abstracts: explanation and elaboration. *PLoS Med* 5(1): e20

3 Ioannidis JP, Evans SJ, Gotzsche PC, O'Neill RT, Altman DG, Schulz K, Moher D. Better reporting of harms in randomized trials: an extension of the CONSORT statement. *Ann Intern Med* 2004; 141(10):781-788.

For peer review only

Table 1: CONSORT 2010 checklist of information to include when reporting a cluster randomised trial

Section/Topic	Item No	Standard Checklist item	Extension for cluster designs	Page No *
**Title and abstract**				
	1a	Identification as a randomised trial in the title	Identification as a cluster randomised trial in the title	1
	1b	Structured summary of trial design, methods, results, and conclusions (for specific guidance see CONSORT for abstracts)[1,2]	See table 2	2
**Introduction**				
**Background and objectives**	2a	Scientific background and explanation of rationale	Rationale for using a cluster design	3-6
	2b	Specific objectives or hypotheses	Whether objectives pertain to the cluster level, the individual participant level or both	6
**Methods**				
**Trial design**	3a	Description of trial design (such as parallel, factorial) including allocation ratio	Definition of cluster and description of how the design features apply to the clusters	7-8
	3b	Important changes to methods after trial commencement (such as eligibility criteria), with reasons		**NA**
**Participants**	4a	Eligibility criteria for participants	Eligibility criteria for clusters	9-10
	4b	Settings and locations where the data were collected		**8-9**
**Interventions**	5	The interventions for each group with sufficient details to allow replication, including how and when they were actually administered	Whether interventions pertain to the cluster level, the individual participant level or both	10-11
**Outcomes**	6a	Completely defined pre-specified primary and	Whether outcome measures pertain to the cluster level, the	11-16

		secondary outcome measures, including how and when they were assessed	individual participant level or both	
	6b	Any changes to trial outcomes after the trial commenced, with reasons		
**Sample size**	7a	How sample size was determined	Method of calculation, number of clusters(s) (and whether equal or unequal cluster sizes are assumed), cluster size, a coefficient of intracluster correlation (ICC or *k*), and an indication of its uncertainty	16-17
	7b	When applicable, explanation of any interim analyses and stopping guidelines		
**Randomisation:**				
**Sequence generation**	8a	Method used to generate the random allocation sequence		**10**
	8b	Type of randomisation; details of any restriction (such as blocking and block size)	Details of stratification or matching if used	10
**Allocation concealment mechanism**	9	Mechanism used to implement the random allocation sequence (such as sequentially numbered containers), describing any steps taken to conceal the sequence until interventions were assigned	Specification that allocation was based on clusters rather than individuals and whether allocation concealment (if any) was at the cluster level, the individual participant level or both	10
**Implementation**	10	Who generated the random allocation sequence, who enrolled participants, and who assigned participants to interventions	Replace by 10a, 10b and 10c	8-10
	10a		Who generated the random allocation sequence, who enrolled clusters, and who assigned clusters to interventions	8-10

	10b		Mechanism by which individual participants were included in clusters for the purposes of the trial (such as complete enumeration, random sampling)	8-10
	10c		From whom consent was sought (representatives of the cluster, or individual cluster members, or both), and whether consent was sought before or after randomisation	8-10
**Blinding**	11a	If done, who was blinded after assignment to interventions (for example, participants, care providers, those assessing outcomes) and how		**NA**
	11b	If relevant, description of the similarity of interventions		**10-11**
**Statistical methods**	12a	Statistical methods used to compare groups for primary and secondary outcomes	How clustering was taken into account	17-18
	12b	Methods for additional analyses, such as subgroup analyses and adjusted analyses		**17-18**
**Results**				
**Participant flow (a diagram is strongly recommended)**	13a	For each group, the numbers of participants who were randomly assigned, received intended treatment, and were analysed for the primary outcome	For each group, the numbers of clusters that were randomly assigned, received intended treatment, and were analysed for the primary outcome	Figure 1
	13b	For each group, losses and exclusions after randomisation, together with reasons	For each group, losses and exclusions for both clusters and individual cluster members	Figure 1

For peer review only - http://bmjopen.bmj.com/site/about/guidelines.xhtml

**Recruitment**	14a	Dates defining the periods of recruitment and follow-up		**To be reported**
	14b	Why the trial ended or was stopped		
**Baseline data**	15	A table showing baseline demographic and clinical characteristics for each group	Baseline characteristics for the individual and cluster levels as applicable for each group	**To be reported**
**Numbers analysed**	16	For each group, number of participants (denominator) included in each analysis and whether the analysis was by original assigned groups	For each group, number of clusters included in each analysis	**To be reported**
**Outcomes and estimation**	17a	For each primary and secondary outcome, results for each group, and the estimated effect size and its precision (such as 95% confidence interval)	Results at the individual or cluster level as applicable and a coefficient of intracluster correlation (ICC or k) for each primary outcome	**To be reported**
	17b	For binary outcomes, presentation of both absolute and relative effect sizes is recommended		
**Ancillary analyses**	18	Results of any other analyses performed, including subgroup analyses and adjusted analyses, distinguishing pre-specified from exploratory		
**Harms**	19	All important harms or unintended effects in each group (for specific guidance see CONSORT for harms[3])		**To be reported**
**Discussion**				20-22
**Limitations**	20	Trial limitations, addressing sources of potential bias, imprecision, and, if relevant, multiplicity of analyses		20-22
**Generalisability**	21	Generalisability (external validity, applicability) of the trial findings	Generalisability to clusters and/or individual participants (as relevant)	20-22

For peer review only

**Interpretation**	22	Interpretation consistent with results, balancing benefits and harms, and considering other relevant evidence	**To be reported (this is just the protocol)**
**Other information**			
**Registration**	23	Registration number and name of trial registry	**2 and 7**
**Protocol**	24	Where the full trial protocol can be accessed, if available	**This is the protocol**
**Funding**	25	Sources of funding and other support (such as supply of drugs), role of funders	**1**

** Note: page numbers optional depending on journal requirements*

For peer review only

Table 2: Extension of CONSORT for abstracts[1,2] to reports of cluster randomised trials

Item	Standard Checklist item	Extension for cluster trials
**Title**	Identification of study as randomised	**Identification of study as cluster randomised**
**Trial design**	Description of the trial design (e.g. parallel, cluster, non-inferiority)	
**Methods**		
**Participants**	Eligibility criteria for participants and the settings where the data were collected	**Eligibility criteria for clusters**
**Interventions**	Interventions intended for each group	
**Objective**	Specific objective or hypothesis	**Whether objective or hypothesis pertains to the cluster level, the individual participant level or both**
**Outcome**	Clearly defined primary outcome for this report	**Whether the primary outcome pertains to the cluster level, the individual participant level or both**
**Randomization**	How participants were allocated to interventions	**How clusters were allocated to interventions**
**Blinding (masking)**	Whether or not participants, care givers, and those assessing the outcomes were blinded to group assignment	
**Results**		
**Numbers randomized**	Number of participants randomized to each group	**Number of clusters randomized to each group**
**Recruitment**	Trial status[1]	
**Numbers analysed**	Number of participants analysed in each group	**Number of clusters analysed in each group**
**Outcome**	For the primary outcome, a result for each group and the estimated effect size and its precision	**Results at the cluster or individual participant level as applicable for each primary outcome**
**Harms**	Important adverse events or side effects	
**Conclusions**	General interpretation of the results	
**Trial registration**	Registration number and name of trial register	
**Funding**	Source of funding	

[1] Relevant to Conference Abstracts

For peer review only

REFERENCES

[1] Hopewell S, Clarke M, Moher D, Wager E, Middleton P, Altman DG, et al. CONSORT for reporting randomised trials in journal and conference abstracts. *Lancet* 2008, 371:281-283

[2] Hopewell S, Clarke M, Moher D, Wager E, Middleton P, Altman DG at al (2008) CONSORT for reporting randomized controlled trials in journal and conference abstracts: explanation and elaboration. *PLoS Med* 5(1): e20

[3] Ioannidis JP, Evans SJ, Gotzsche PC, O'Neill RT, Altman DG, Schulz K, Moher D. Better reporting of harms in randomized trials: an extension of the CONSORT statement. *Ann Intern Med* 2004; 141(10):781-788.

For peer review only

BMJ Open

Health promoting work schedules: Protocol for a large-scale cluster randomized controlled trial on the effects of a work schedule without quick returns on sickness absence among healthcare workers

Journal:	*BMJ Open*
Manuscript ID	bmjopen-2021-058309.R1
Article Type:	Protocol
Date Submitted by the Author:	17-Jan-2022

Complete List of Authors:	Vedaa, Oystein; Norwegian Institute of Public Health, Department of Health Promotion; Norwegian University of Science and Technology, Department of Mental Health<br>Djupedal, Ingebjørg Louise Rockwell; University of Bergen, Department of Psychosocial Science<br>Svensen, Erling; Haukeland Universitetssjukehus<br>Waage, Siri; University of Bergen, Department of Global Public Health and Primary Care<br>Bjorvatn, Bjørn; Universitetet i Bergen Det medisinsk-odontologiske fakultet, Department of Global Public Health and Primary Care; Haukeland Universitetssjukehus, Norwegian Competence Center for Sleep Disorders<br>Pallesen, Ståle; University of Bergen<br>Lie, S; University of Bergen, Department of Dentistry<br>Nielsen, Morten; University of Bergen<br>Harris, Anette; Universitetet i Bergen Det Psykologiske Fakultet

**Primary Subject Heading**:	Occupational and environmental medicine
Secondary Subject Heading:	Public health
Keywords:	OCCUPATIONAL & INDUSTRIAL MEDICINE, Adult psychiatry < PSYCHIATRY, SLEEP MEDICINE

SCHOLARONE™
Manuscripts

Health promoting work schedules: Protocol for a large-scale cluster randomized controlled trial on the effects of a work schedule without quick returns on sickness absence among healthcare workers

Øystein Vedaa[1,2,3], Ingebjørg Louise Rockwell Djupedal[4], Erling Svensen[5], Siri Waage[6,7], Bjørn Bjorvatn[6,7], Ståle Pallesen[4,7,8], Stein Atle Lie[9], Morten Birkeland Nielsen[4,10], Anette Harris[4]

[1] Department of Health Promotion, Norwegian Institute of Public Health, Bergen, Norway;

[2] Department of Mental Health, Norwegian University of Science and Technology, Trondheim, Norway;

[3] Department of Research and Development, St Olavs University Hospital, Trondheim, Norway

[4] Department of Psychosocial Science, University of Bergen, Bergen, Norway;

[5] Haukeland University Hospital, Bergen, Norway;

[6] Department of Global Public Health and Primary Care, University of Bergen, Bergen, Norway;

[7] Norwegian Competence Center for Sleep Disorders, Haukeland University Hospital, Bergen, Norway;

[8] Optentia at the Vaal Triangle Campus of the North-West University, South-Africa;

[9] Department of Clinical Dentistry, University of Bergen, Bergen, Norway;

[10] National Institute of Occupational Health, Oslo, Norway

Correspondence to: Øystein Vedaa, Department of Health Promotion, Norwegian Institute of Public Health, Zander Kaaes gate 7, 5018 Bergen, Norway; e-mail: Oystein.Vedaa@fhi.no

Word count: 5975

Date and version identifier:

- Issue date: 14 Jan 2022

Protocol amendment number: 02

For peer review only

Abstract

Introduction In shift work, quick returns refer to transitions between two shifts with less than 11 hours available rest time. Twenty-three per cent of employees in European countries reported having quick returns. Quick returns are related to short sleep duration, fatigue, sleepiness, work-related accidents, and sickness absence. The present study is the first randomized controlled trial (RCT) to investigate the effect of a work schedule without quick returns for six months, compared to a work schedule that maintains quick returns during the same time frame.

Methods and analysis A parallel-group cluster RCT in a target sample of about 4000 healthcare workers at Haukeland University Hospital in Norway will be conducted. About 70 hospital units will be randomized to a work schedule without quick returns for six months or continue with a schedule that maintains quick returns. The primary outcome is objective records of sickness absence; secondary outcomes are questionnaire data (n ≈ 4000 invited) on sleep and functioning, physical and psychological health, work-related accidents, and turnover intention. For a subsample, sleep diaries and objective sleep registrations with radar technology (n ≈ 50) will be collected.

Ethics and dissemination The study protocol was approved by the Regional Committee for Medical and Health Research Ethics in Western Norway (2020/200386). Findings from the trial will be disseminated in peer-reviewed journals and presented at national and international conferences. Exploratory analyses of potential mediators and moderators will be reported. User-friendly outputs will be disseminated to relevant stakeholders, unions and other relevant societal groups.

Trial registration number NCT04693182; Pre-recruitment.

Key words: Quick returns, Shift work, Sickness absence, Sick leave

For peer review only

Article summary:

Strength and limitations of the study:

- This is the first randomized controlled trial to investigate the effect of a work schedule without quick returns.
- The primary outcome measure is objective register data on sickness absence with no missing data.
- As this is an evaluation of an organizational quality improvement measure implemented for all employees at the hospital, we get to study the effect on the entire target population with full representativeness.
- One concern in this trial is how well the intervention group will succeed in abolishing quick returns from the shift schedule (given that this is a study conducted in a naturalistic setting).
- Another concern in this trial is that a shift schedule that does not include quick returns may unintentionally include other unfavourable shift characteristics that could potentially confound the results (e.g., more consecutive evening shifts).

For peer review only

INTRODUCTION

An important principle when planning shift schedules is that employees are apportioned sufficient time to rest and recover between shifts. According to the EU's Working Time Directive (2003/88 / EC),[1] employees are entitled to minimum 11 hours of rest between two consecutive shifts. Still, in some countries, including Norway, employers and the employees' representatives can agree on rest periods less than 11 hours between two shifts. In this realm, the term *quick return* refers to transitions between two shifts with less than 11 hours available rest time. Quick returns occur most often between an evening shift and a day shift the following day, but can also occur between a night shift and an evening shift, and between a day shift and a night shift the subsequent night.[2] In the sixth European Working Conditions Survey published in 2016,[3] 23 per cent of employees in European countries reported having at least one quick return during the last month. Quick returns seem to be particularly prevalent in the healthcare sector. In a large Danish register survey (n = 69,200), it was shown that, on average per year, 65 per cent of nurses, 38 per cent of physicians, and 26 per cent of medical secretaries had quick returns in their work schedule.[4]

Eleven hours define the upper limit of potential time for rest between two shifts in a quick return, while the actual time available is often substantially shorter. A Norwegian study investigating payroll data from nurses found that almost 2/3 of the quick returns involved rest time less than 9 hours between two shifts, and some employees (2%) even had rest time of less than 7 hours.[5] The time available for sleep and recuperation is further curtailed by the time it takes to commute to and from work, time for self-care, meals, family obligations and house chores. A systematic literature review reported that sleep duration in quick returns between evening and day shifts typically is reduced to 5-6.5 hours, compared to 7-8 hours on non-quick return nights.[2] In addition to reduced sleep duration, the most robust findings in the literature review were that quick returns were associated with more fatigue, higher levels of

For peer review only

sleepiness, and shift work disorder (i.e., sleep problems or sleepiness related to a recurring shift schedule). Individual studies also showed that quick returns were associated with poorer sleep quality, impaired general health and well-being, higher self-reported stress, and lower job satisfaction.[2]

The most immediate consequence of quick returns is probably shortened sleep.[6] It is reasonable to think that this in turn leads to a number of other negative consequences. In a diary study (sleep- and work-schedule), we found that nurses reported higher sleepiness during the day shift when they had quick return to the day shift, as compared to during other regular day shifts.[6] In fact, the results showed that the nurses were as sleepy during the day shift after a quick return as they were during night shifts. It is conceivable that high sleepiness represents a greater problem when it occurs during day shifts than during night shifts, since day shifts are often busier[7] and typically experienced as more stressful.[6] The combination of a high level of sleepiness during a stressful shift might represent a type of circumstance that increases the risk of accidents. Indeed, the association between quick returns and work-related accidents or injuries is established in previous research. In a large register-based study from Denmark, researchers linked payroll data of healthcare workers with national registers of injuries. The results showed that quick returns were associated with a 39 per cent higher risk of injury, compared with having 15-17 hours off between two shifts.[4] A longitudinal study found an increased risk of needlestick injuries among nurses who reported having quick returns as compared to nurses without quick returns.[8] A study based on cross-sectional data found that quick returns were associated with an increased risk of falling asleep at work, of experiencing work-related injuries to themselves, of injuring patients or others, and of damaging equipment at work.[9] In fact, the risk of experiencing injuries to themselves and damaging equipment at work was greater with quick returns than with night shifts. Another longitudinal study, partly based on the same data, demonstrated that nurses who experienced

For peer review only

an increase in the number of quick returns over time also had an increased risk of work-related accidents, whereas a decrease in the number of quick returns over time was associated with reduced risk of accidents.[10]

Over the past five years, researchers have increasingly begun to use register/payroll data on exposure to shift work when examining the consequences of different shift characteristics. These data are registered by the employees, typically at healthcare institutions and include information about the date and start and stop time for all shifts performed. In some cases, it is also possible to retrieve data on sickness absence from the same registers. These data comprise information on the date of each day of absence (self-certified and medically certified absence) due to illness. In a Finnish study using such register data from healthcare workers, the relationship between quick returns and short-term sick leave (1 to 3 days) was investigated. The results showed that having few quick returns (defined as 3 or fewer over a period of 28 days) was associated with a lower risk of short-term sick leave, while having many quick returns (5 or more over a period of 28 days) was associated with a higher risk of short-term sickness absence, compared to having no quick returns.[11] In a study based on Danish and Finnish register data, it was found that healthcare workers who had at least 13 quick returns during a year had a higher risk of long-term sick leave than those with fewer quick returns.[12] These findings are in line with results using corresponding register data in Norway.[5] In one study, the findings showed that exposure to quick returns one month was associated with a higher risk of sick leave the following month. On average, nurses had 3 quick returns per month, which corresponded to 21 per cent more sickness absence days the subsequent month (over and above the sickness absence days of workers without quick return).[5]

Research on quick return and health and safety related outcomes have so far all been based on correlational studies. We do not yet know whether these health outcomes are caused by

For peer review only

exposure to quick returns. The present study is the first randomized controlled trial (RCT) conducted to determine the effects of abolishing quick return from the work schedule.

Aims

This paper describes the protocol for a two-arm cluster randomized controlled trial that assesses the consequences of a shift work schedule abolishing quick returns, compared to a schedule maintaining quick returns for a six months period. First, we will examine any differential change in sickness absence (primary outcome) during the six-month intervention period. Second, we will examine if there are differential changes in sleep and functioning, physical and mental health, work-related accidents, and turnover intention, among others (secondary outcomes). Third, we will investigate if individual characteristics associated with shift work tolerance including sex, age, personality and subjectively reported sleep need moderate the negative effects of quick returns on the primary and secondary outcomes. Finally, the study will investigate if individual factors like satisfaction with work schedule, job satisfaction, job engagement and work-family interference moderate the negative effects of quick returns on the primary and secondary outcomes.

For peer review only

METHODS AND ANALYSIS

The protocol for the current trial follows the SPIRIT checklist for intervention trials. The trial is further pre-registered with the Clinical Trials website (ClinicalTrials.gov identified: NCT04693182).

Figure 1 shows the Flow Diagram for the current trial. The flow chart illustrates the timeline for recruitment, randomization, assessments and for undertaking primary and secondary analyses.

Insert Figure 1 about here

Research design

A cluster randomized controlled trial comparing a six months work schedule abolishing quick returns (intervention) with that of a six months work schedule maintaining a normal amount of quick returns (control) will be conducted. The clusters in this trial represent hospital units that are randomly selected to receive (or not receive) the intervention. 'Normal amount of quick returns' refer to that which is the common practice at the respective hospital unit in recent years (i.e., when no explicit changes have been made to the work schedule), which means that the total number of quick returns at the unit will vary from 329–2356 per year (on average, nurses have three quick returns per month at this hospital[5]). In September 2020, the hospital units were informed about the conditions they would be randomized to at the start of the study in 2021. Thus, the autumn of 2020 was spent planning the shift schedule for 2021 (i.e., removing quick returns for the intervention group and maintaining quick returns for the control group). Most hospital units started the intervention period in the first half of 2021,

For peer review only

while some units started the intervention period in the second half of 2021.The intervention period in this study is six calendar months.

The primary outcome is sickness absence retrieved from the local registers kept by the hospital (including short- and long-term sick leave). The baseline measurements will be sickness absence from the year preceding the intervention, which for each individual participant will be matched on duration and season to that of the intervention period. We will apply for ethical approval to use the register data from all employees at the randomised hospital units without obtaining individual consent. In addition, a consent-based part of the trial will be conducted, in which secondary outcome measures will be collected via questionnaire at baseline and six-month follow-up. All employees (n ≈ 4000) at the randomized units will be asked to complete a digital questionnaire. This will be made available to the employees when they log on to enter their working hours ("MinGat"). Baseline assessment will occur prior to the intervention period, and follow-up assessment will occur towards the end of the intervention period. A subsample (n ≈ 50) will be asked to record their sleep with advanced sleep radar technology (Somnofy™)[13] and subjectively with sleep diaries for ≥1 week at the baseline and follow-up assessments, respectively.

Participants and procedure

Recruitment

This trial is carried out in collaboration with the human resources department at Haukeland University Hospital, Bergen, Norway. All hospital care units that have 24-hour staffing at Haukeland University Hospital will be considered for inclusion in this trial. This will include all healthcare workers working shifts, except for physicians. Physicians are to be excluded since they often have a different shift schedule and compensation scheme compared to other occupational groups at the hospital. Hereinafter, 'all employees' refer to all healthcare workers

For peer review only

engaged in shift work at the randomised hospital units, except for physicians. All employees (n ≈ 4000) at the randomized hospital units will be asked to complete a questionnaire prior to, and at the end of, the intervention period. Recruitment for this part of the trial will take place via the hospital's internal website or through the site in which the employees enter their working hours ("MinGat"). Researchers (the authors of this paper) and human resources personnel at the hospital will attend staff meetings at all included units to inform about the research project and encourage participation. A subsample of n ≈ 50 employees (evenly distributed from the intervention and the control units) will be recruited by convenience for the objective sleep monitoring section of the trial.

Eligibility

The unit-level inclusion criteria are that the units should have 1) healthcare workers (other than physicians) who work rotating shifts, 2) employees who regularly have quick returns in their work schedule, and 3) a new shift rotation year commencing from the first half of 2021 (which is the case for most units at the included hospitals). Exclusion criteria at the unit-level are 1) units recently (or will in the near future) went through other major organizational changes that may confound the results of the trial (this includes during the period from one year before the intervention starts until the intervention period is over), or 2) unit's manager or a substantial number of employees strongly oppose participation. Haukeland University Hospital had a total of 76 units which were considered for eligibility, 67 of which were deemed eligible for the trial. **Figure 1** provides an overview of the number of units excluded before the randomization took place.

This trial consists of three different data collections with an expected dissimilar number of participants: A) a register study, i.e. the primary investigation, in which we expect no missing data, B) a questionnaire study, i.e. the secondary investigation, with an expected response rate of 40-50 per cent,[14] and C) the sleep monitoring study, i.e., secondary investigation,

For peer review only

conducted on a subsample of ≈50 employees recruited by convenience. All employees from the randomised hospital units working ≥80 percent of full-time equivalent will participate in the register-based study (investigation A) and the same group will be asked to participate in the questionnaire-based study (investigation B). Finally, participants in the sleep monitoring study (investigation C) will be recruited by convenience from the same sample of healthcare workers requiring that they are working ≥80 percent of full-time equivalent.

Randomisation and masking

The randomization in this trial occurred at the cluster level, in which hospital units constituted the clusters. As shown in Figure 1, a total of 67 hospital units were randomized. Hospital units can vary in terms of how much staff they need over the 24-hour day, hence, the work schedule and the occurrence of, for example, quick returns and night shifts can vary across the units. Similar units were therefore grouped together based on the fact that they shared some attributes or characteristics. Then a stratified randomization was performed to the two study conditions in a 1: 1 ratio. One subgroup could, for example, consist of units with emergency functions, another with intensive care functions, one with mental health care, and one with maternity care, etc. In total we had 10 strata and the sizes of each stratum varied between 2 and 19 hospital units. The randomization list for each stratum was generated by the online randomization webpage, www.randomization.com, and the list for each stratum was saved.

It is not possible for participants to be blinded to the group to which they are assigned. However, statistical analyses will be done by a researcher who is masked to group allocation.

Intervention

The intervention entails implementing a shift schedule which abolishes quick returns for a six-month intervention period. The mean number of quick returns in the various hospital units in this trial varies from 3–32 per year. The intervention means that this number is abolished or

For peer review only

reduced as much as possible. For practical reasons the intervention may be a matter of reducing rather than completely abolishing quick returns. This might be in the case of ensuring adequate staffing (e.g., due to sickness absence), and since employees for various reasons may make short-notice shift swaps in which it is not possible to comply with the rule of avoiding quick returns. The human resources department at the hospital will assist shift planners in identifying appropriate shift schedules that do not include quick returns. **Table 1** shows some of the examples that were used to show shift planners how this could be done.

The control condition in this trial implies that employees maintain the same number of quick returns as in previous years for the six-month intervention period. It is important to note that hospital units in the control group are not expected to experience any increase in the number of quick returns.

Insert Table 1 about here

Assessments

All assessments/instruments in this trial are described below. **Table 2** provides an overview of the source and timing of the assessments. The primary outcome in this trial is sickness absence (number of days or spells). We will compare the sickness absence in intervention group with the control group during the intervention period, while adjusting for previous sick leave from the corresponding period the year preceding the intervention (matched on duration and season). Other measures included in this trial are secondary outcomes or outcomes used in exploratory or subsidiary analyses.

Demographics

For peer review only

Demographic information will be obtained both from the register at the hospital as well as from a questionnaire. Information on sex, age and percentage of full-time equivalent will be available from the register data; while information on marital status, highest completed education/degree, years of experience with shift work, and if the participant has children living at home will be collected through the questionnaire.

Primary outcome

Sickness absence data will be retrieved from the local records kept by the hospital.[5] This record includes information about the date of any absence of the individual employee, implying that it includes information about both short- and long-term sickness absence. Further, these data include information on whether the absence is self-certified or whether it is certified by a physician, whether the absence is due to a sick child of whom the employee has childcare responsibility of, and whether the absence is due to COVID-19 related issues (e.g., quarantine).

Secondary outcomes

The Bergen Insomnia Scale (BIS)[15] will be used to measure sleep problems among participants. The scale originally comprised six items that assess symptoms of insomnia. An additional item will be included to the scale in which we will ask about the duration of any sleep problems. This makes it possible to define insomnia according to the diagnostic criteria in the International Classification of Sleep Disorders-Third Edition,[16] Diagnostic and Statistical Manual of Mental Disorders-Fifth Edition, and the International Classification of Diseases-11th Revision.

Shift work disorder (SWD) will be measured with three standardised questions.[17] SWD was evaluated with three questions based on the criteria from the third edition of the International Classification of Sleep Disorders (ICSD-3).[16] The questions were: a) Do you have a work

For peer review only

schedule that sometimes overlap with the time you usually sleep?, b) if yes, does this cause insomnia and/or excessive sleepiness due to reduced amount of sleep?, c) if yes, has this lasted for at least three months? Participants will be classified as having SWD when responding “yes” to all three questions.

The revised Swedish Occupational Fatigue Inventory (SOFI) will be used to measure lack of energy, physical exertion, physical discomfort, lack of motivation and sleepiness.[18] Participants are asked to indicate the extent to which they have recently (or for a specified period of time) experienced a list of 20 psychological and physical sensations related to fatigue.

The revised Circadian Type Inventory (rCTI) comprises 11 items, five of which assesses flexibility and six assesses languidity.[19] High scores on flexibility reflect better ability to sleep and work at odd times, whereas high scores on languidity indicate difficulties overcoming drowsiness and feelings of lethargy following sleep loss.

The Horne-Östberg Morningness Eveningness Questionnaire (MEQ) is the most widely used morningness-eveningness inventory,[20] and is designed to determine preferred timing of sleep and activities during the 24-hour day.[21] The MEQ reduced version (rMEQ) will be used in the present trial, which is comprised of five items from the original scale.[22]

Hopkins Symptoms Checklist - 5 (HSCL-5) will be used to measure general psychological distress.[23] HSCL-5 includes five questions about nervousness or inner turmoil, fear or feeling anxious, feeling hopeless about the future, depression or melancholy, worry or restlessness. An average score can be calculated across the five items with values that vary from 1 to 4, in which higher scores indicate a higher degree of psychological distress. The composite score is sometimes recoded into a two-part variable in which a score higher than 2.00 is defined as a high score.

For peer review only

Job Satisfaction Index (JSI) comprise five items measuring satisfaction with work (e.g., "I find real enjoyment in my work").[24] Each item is answered on a 5-point Likert scale, ranging from 1 (strongly disagree) to 5 (strongly agree). Higher scores reflect higher levels of overall job satisfaction.

The Work Family Interface Scale[25] will be used to evaluate the four types of work–family spillover. Consisting of 14 items, the scale was designed to measure both negative and positive work–to– family (NWFS and PWFS) and family–to–work spillover (NFWS and PFWS). The responses were graded by a frequency based on a 1–5 Likert scale, with alternatives ranging from never to very often.

Work-related negative incidents will be assessed using eight items measuring the number of self-reported work-related accidents, near accidents and dozing off at work or while driving to or from work. These questions have been developed in connection with the Norwegian Survey of Shift work, Sleep and Health among Nurses (SUSSH), and have been used in several previous publications.[26]

The Turnover Intention Scale (TIS) will be used to measure turnover intention, which is comprised of three items adapted from Michigan Organizational Assessment Questionnaire.[27] The three items are: "I will actively look for a new job in the next year," "I often think about quitting," and "I will probably look for a new job by the next year." Responses were recorded on a 5-point Likert scale from 1 (strongly disagree) to 5 (strongly agree), yielding a score range of 3–15. A high score indicates a high degree of turnover intention.

The Utrecht Work Engagement Scale (UWES - 9) will be used to measure work engagement.[28] The UWES is originally comprised of 17 items rated on a 7-point scale ranging from "never" (0) to "always/every day" (6). The 9-item version of the UWES includes three items for each of the three factors; Vigor (e.g., "At my job, I feel strong and vigorous"),

For peer review only

Dedication (e.g., “I am enthusiastic about my job”), and Absorption (e.g., “When I am working, I forget everything else around me”). A higher score indicates more work engagement.

Subjective Health Complaints inventory (SHC)[29] consists of a list of 29 common health complaints that participants grade the intensity of which they experience each complaint on a four-point scale (0 = not at all; 1 = a little; 2 = some; 3 = severe). In this study, we include three of the five subscales; i.e. musculoskeletal complaints, pseudoneurological complaints, and gastrointestinal complaints.

Recovery Experience Questionnaire (REQ)[30] will be used to measure recovery experiences. REQ is originally a 16-item questionnaire with the four subscales psychological detachment, relaxation, mastery, and control. The present study includes the subscales of psychological detachment and relaxation. Each item is scored on a 5-point Likert scale ranging from 1 (strongly disagree) to 5 (strongly agree) of which a higher score indicates better detachment/relaxation.

Epworth Sleepiness Scale (ESS)[31] will be used to measure participants sleepiness. ESS is an eight-item questionnaire asking the participants how likely they are to doze off or fall asleep in different situations of everyday life including (e.g. while sitting and reading, watching TV, when sitting and talking to someone, etc.). For each item, participants report the chance of dozing as never (0), slight (1), moderate (2), or high (3) (total score range between 0 – 24). A higher score indicates higher level of sleepiness.

Additional measures of unwanted/negative effects and other exploratory analyses

Other factors that may have an impact on how the employees react to the intervention will also be investigated. The participants' attitudes to the intervention and the research project will be measured, in addition to how they experience the implementation of the intervention.

For peer review only

A set of questions measuring possible negative or unwanted effects of the intervention will be developed for the purpose of this trial. These questions will specifically ask if the changed work schedule has led to disturbed sleep, more stress, worry, depression, overall less time for recovery between work periods, problems in work-family balance, disrupted social relationships, poorer psychosocial climate at work, experience of reduced quality of care offered to patients, etc. For some employees, it is possible that a work schedule that does not allow for quick returns represents a restricted opportunity to co-design their schedule (i.e., self-rostering) and reduces the duration of free periods. Therefore, we will measure the participants' perceived change in relation to these parameters. Furthermore, we will include questions about satisfaction with work schedule, commute time, habitual and preferred sleep duration, current use of prescribed or over-the-counter sleep medication, current use of light treatment to improve sleep, and participants' physical activity level. Finally, the questionnaire will include an open text box in which participants can write freely, for example about anything they would like to convey related to the intervention (e.g. topics/themes they felt was inadequately addressed in the survey).

Sleep will be assessed more thoroughly for a subsample of ≈50 employees. The measures of sleep will include daily self-rating of sleep-wake patterns reported using the consensus sleep diary,[17] as well as sleep measured objectively using the Xethru sensor, a low-powered ultra-wideband radar.[32] The sleep registration will occur for ≥7 days at baseline and at six-month follow-up.

Sample size

In this trial, all available hospital units at Haukeland University Hospital with healthcare workers who work rotating shifts were assessed for eligibility. This included 76 units and 4260 healthcare workers. As shown in Figure 1, a total of 67 of these units were finally included, i.e. 3669 healthcare workers. Based on previous published data[5] we have calculated

For peer review only

that a total of 2028 participants is sufficient to reveal a difference in days of sick leave of 0.9 and 1.25 with an ICC of 0.1 and an average size of the units of 52 (calculation made in: StataCorp. 2015).[33] With 67 hospital units and 3669 participants, we will thus be well within the number of participants required for the primary outcome variable and we consider this sufficient for all conceivable purposes of this trial.

Data analysis plan

All analyses will be conducted based on the intention-to-treat population, unless otherwise stated. To examine the effects of a shift schedule abated of quick returns on primary and secondary outcomes, the observed rates or scores will be analysed by means of latent growth models (or other equivalent models such as generalized linear mixed models). The observed rates or scores before and during the intervention period will be modelled by a random intercept and a fixed slope. The effect of the intervention will be estimated by using the group variable (intervention vs. control) as a predictor of the slope. Between-group effect sizes (Cohen's d) will be calculated by dividing the mean difference in estimated change in scores from baseline to the follow-up assessment by the pooled SD at baseline. Robust maximum likelihood will be used as the estimator, providing unbiased estimates under the assumption of data being missing at random,[34] which might be partly met through the inclusion of baseline scores to the model. The primary outcome measure in this trial is sickness absence data retrieved from the register at the hospital, in which we expect no missing data. However, it is reasonable to expect some missing data on the secondary outcome measures, as data are collected through questionnaire or via the sleep radar and sleep diary.

As some data for the follow-up questionnaire and sleep radar/diary assessment will be missing not at random, the robustness of the results under the missing-at-random assumption will be tested by sensitivity analyses in which the missing scores at follow-up will be replaced by baseline values for each respective individual. Since it is possible to imagine that some

For peer review only

participants may experience worsening because of the intervention, we will consider carrying out more rigorous sensitivity analyses. For example, by replacing missing scores at the follow-up assessment with baseline scores multiplied by a given factor (higher or lower than 1.00 depending on the direction that indicates a worsening) in the intervention group and by 1.00 in the control group. These sensitivity analyses will only be performed on selected variables depending on the focus in the respective article.

The intention-to-treat analyses may be accompanied by selected per-protocol analyses in which we, based on payroll data, define a group that has completely abolished or had a satisfactory reduction in the number of quick returns during the intervention period.

The primary outcome of sick leave will mainly be analysed in terms of the total number of sickness absence days and periods (spells) for a given period *before* compared to *during* the intervention period.[5] The models of sickness absence will take into account the zero inflation in this type of data. Other operationalisations of sickness absence might also be considered in accordance with recommendations in the literature.[35] For a further investigation of the sickness absence data, we will consider the use of more complex survival analyses (e.g., Cox proportional hazards model), and we will also consider modelling time to return to work (from sickness absence) and/or time before taking sickness absence according to group allocation.

Since the introduction of a work schedule without quick returns may entail an alternative schedule with an increase in other undesirable characteristics (e.g., more consecutive evening shifts), we will consider conducting analyses that adjust for such characteristics.

Mediator and moderator analyses will be performed for exploratory purposes, based on the basic principle for such analyses in randomised controlled trials as described by others (e.g., [36]). For example, some of the data collected on demographics, sleep-related personality traits

For peer review only

(rCTI and MEQ), mental health, among others, can be used to examine factors that may moderate the impact of the intervention.

Stakeholder and public involvement

This trial is carried out in close collaboration with the HR department at Haukeland University Hospital. In addition, representatives from all relevant trade unions at the hospital will be involved in the planning and implementation of the research project. The findings of the trial will be disseminated via scholars in terms of scientific paper and conference presentations, and by stakeholder/union advocacy and other relevant public and community groups. Further, Haukeland University Hospital will arrange a conference for other relevant stakeholders, in which research results will be presented and the implications of the findings will be discussed.

Patient involvement

No patient involved.

Ethics and dissemination

The study protocol was approved by the Regional Committee for Medical and Health Research Ethics in Western Norway (2020/200386). In this trial, all employees at the included hospital units will be randomized to one of two conditions, and we will retrieve register data on working hours and sickness absence without collecting individual consent. This poses an ethical dilemma since all participation in research – especially when people are exposed to an intervention – should be consent-based. However, the intervention in this trial is to abolish or substantially reduce quick returns, and *not* to increase any exposure. This is thus considered not to represent a significant burden on the participants, as the presence of quick returns is already a violation of the Working Environment Act. In addition, we expect that the intervention primarily will have beneficial effects on employees' health and safety.

For peer review only

Abolishing or reducing the number of quick returns is a quality improvement measure that the Health Trust wants to implement independently of the present research project. The fact that the intervention is carried out as a research project is considered an advantage for the employees, as far as we are able to uncover any unintended negative effects of the intervention and further to be able to empirically document potential benefits on health and safety.

The result of this trial will potentially impact subsequent standards and practice when it comes to planning shift schedules and their compliance with the Working Environment Act. As vast number of employees might be affected by the trial results, it is equally important that the results are representative of the employees. We believe this justifies the use of the employees' register data without obtaining individual consent.

Participants will be required to provide informed consent before participating in the questionnaire and sleep diary/radar part of the trial. The recruitment and consent process emphasizes that participation is voluntary and that participants can withdraw from this part of the trial at any time point without any consequences. Self-report data are recorded in electronic files that are encrypted and password protected. No identifying information will be stored alongside the self-report data. Furthermore, only researchers directly involved in data analysis will be granted supervised access to de-identified participant data.

Findings from this randomized controlled trial will be disseminated in peer-reviewed publications and as conference presentations. After the research project is completed, Haukeland University Hospital will arrange a conference for stakeholders where the results and experience from the research will be disseminated and discussed.

DISCUSSION

For peer review only

To the best of our knowledge, this is the first randomized controlled trial to investigate the effect of a work schedule abolishing quick returns. Previous research on quick returns has been dominated by cross-sectional studies and a few longitudinal investigations. Although quick returns have consistently been associated with negative health and safety outcomes, it is unclear whether quick returns are the cause of these negative outcomes. This trial will thus be the first sincere attempt to establishing such a causal relationship.

There are several major strengths to this trial. The intervention is carried out in all eligible hospital units at Haukeland University Hospital, in which we retrieve objective register data (notably with no missing data) on the primary outcome measure – sickness absence. Hence reporting bias such as social desirability and memory biases will be avoided. This study is unique as it will imply complete access to the entire target population, also including individuals who typically choose not to participate in such studies. Hence this ensures full representativeness, strengthening the external validity of the study. Further, we have access to objective data on exposure to shift work (quick returns and other shift characteristics) during the intervention period. This provides us the opportunity to accurately assess compliance with the intervention and the true reduction in quick returns that occur, as well as monitoring other systemic differences that might occur in the shift schedule between the two parallel conditions. It is also an asset that we combine objective data with data collected via questionnaire. This provides us the opportunity to study the effect of abolishing or reducing quick returns on sleep, health and safety, as well as being able, for example, to study potential moderators to any effects we observe.

There are also some possible limitations with this trial that should be mentioned. The trial is conducted in a naturalistic setting which does not allow for the same strict control as generally would be preferred in experimental designs. One main concern is how well the intervention group will succeed in abolishing quick returns from the shift schedule. We expect that for

For peer review only

many individuals it will be a matter of reducing the number of quick returns, rather than complete abolition, for example, since such shift transitions occasionally may be necessary to ensure adequate staffing. Another concern is that a shift schedule that does not include quick returns may unintentionally include other unfavourable shift characteristics that could potentially confound the results. However, during the implementation of the trial, shift planners are provided with recommendations on how to set up shift schedules without quick returns, e.g. avoiding backward shift rotations, which as far as possible avoids other unfavourable shift characteristics. Further, for the participants in this trial it will be obvious which study condition they have been allocated to, thus their expectations can potentially have an impact on results based on self-reported data.[37] A questionnaire was used to measure most secondary outcome variables in this trial. An important limitation with such subjective reports is possible bias related to the validity of the instruments and recall bias.[38] However, most of the variables were based on standardized questionnaires with adequate psychometric properties. Furthermore, most variables are subjective by their very nature and need accordingly to be measured with self-reports.

If a shift schedule without quick returns is shown to be associated with less sickness absence or positive effects on other outcomes compared to a control group, this may encourage a stricter compliance with the workers' right to have at least 11 hours off between two subsequent shifts. The results of this trial will provide valuable information to stakeholders (nurses responsible for developing shift schedules, trade unions, politicians, and innovators) about the effect of quick returns and individual tolerance to quick returns.

For peer review only

Author statement: AH, ØV, SP, BB, SW, SAL, ES, and MBN conceived the study. ØV and ILRD produced the first draft of the manuscript. All authors assisted in drafting of the final, submitted version of manuscript and all authors have approved this version.

Conflict of Interest: The authors declare that they have no conflict of interest.

Funding: The study was funded from The Research Council of Norway (303671) and the University of Bergen, Bergen, Norway. The Research Council of Norway, Drammensveien 288, 0283 Oslo, Norway, Telephone: +47 22 03 70 00, E-mail: post@forskningsradet.no. University of Bergen, P.O.Box 7800, 5020 Bergen, Norway, Telephone: +47 55 58 80 81, E-mail: post@uib.no. The sponsors had no role in a study design, collection, management, analysis, and interpretation of data; writing of the report; and the decision to submit the report for publication, including whether they will have ultimate authority over any of these activities. The sponsors had no authority over any of the above activities.

Data statement: De-identified data that underlie the results reported from the trial described in this protocol will be available to researchers from accredited research institutions. Access to data will be limited to investigators who provide a methodologically sound proposal and will be limited to a specified time period (commencing about 3 months after publication of a respective Article and ending after 5 years). To ensure compliance with the General Data Protection Regulation, data processing must be covered by the European Commission's standard contractual clauses for the transfer of data, which must be signed by the data requesters. Proposals and requests for data access should be directed to the corresponding author of the respective Article. User-friendly output from the trial will be disseminated to stakeholder and other relevant organisations.

Acknowledgments: We would like to thank Ljiljana Djuric-Rakovic and John Olav Larssen at Haukeland University Hospital for their invaluable help in setting up and distributing the electronic questionnaires for this study. We would also like to thank Helga Berdal Lorentzen and Ole-Daniel Tuft Virkesdal at the HR department at Haukeland University Hospital, and employee representatives of the Norwegian Nurses Organisation, Trade Union Delta, the joint organization for Child Welfare Educators, Social Workers and Learning Disability Nurse and others trade unions for their support and contribution in the implementation of this research project. We would also like to thank Lukas Krondorf at Vital Things AS for technical support during the registration of nurses' sleep using radar technology.

For peer review only

References

1. EU Directive. Directive 2003/88/EC of the European Parliament and of the Council of 4 November 2003 concerning certain aspects of the organisation of working time. In: European Parliament, ed., 2003.

2. Vedaa Ø, Harris A, Bjorvatn B, et al. Systematic review of the relationship between quick returns in rotating shift work and health-related outcomes. *Ergonomics* 2016;59:1-14.

3. European Foundation for the Improvement of Living and Working Conditions. Sixth European working conditions survey–overview report. Luxembourg: Publications Office of the European Union., 2016.

4. Nielsen HB, Hansen ÅM, Conway SH, et al. Short time between shifts and risk of injury among Danish hospital workers: a register-based cohort study. *Scandinavian Journal of Work, Environment & Health* 2018 doi: 10.5271/sjweh.3770

5. Vedaa Ø, Pallesen S, Waage S, et al. Short rest between shift intervals increases the risk of sick leave: a prospective registry study. *Occupational and Environmental Medicine* 2017;74:496-501.

6. Vedaa Ø, Mørland E, Larsen M, et al. Sleep detriments associated with quick returns in rotating shift work: A diary study. *Journal of Occupational and Environmental Medicine* 2017;59:522-27.

7. Nicoletti C, Spengler CM, Läubli T. Physical workload, trapezius muscle activity, and neck pain in nurses' night and day shifts: a physiological evaluation. *Applied ergonomics* 2014;45:741-46.

8. Trinkoff AM, Le R, Geiger-Brown J, et al. Work schedule, needle use, and needlestick injuries among registered nurses. *Infection Control and Hospital Epidemiology* 2007;28:156-64. doi: 10.1086/510785

9. Vedaa Ø, Harris A, Erevik E, et al. Short rest between shifts (quick returns) and night work are associated with work related accidents. *International Archives of Occupational and Environmental Health* 2019;92:829-35. doi: doi.org/10.1007/s00420-019-01421-8

For peer review only

10. Vedaa Ø, Harris A, Waage S, et al. A longitudinal study on the association between quick returns and occupational accidents. *Scandinavian Journal of Work, Environment & Health* 2020 doi: 10.5271/sjweh.3906

11. Ropponen A, Koskinen A, Puttonen S, et al. Exposure to working-hour characteristics and short sickness absence in hospital workers: A case-crossover study using objective data. *Int J Nurs Stud* 2019;91:14-21.

12. Larsen AD, Ropponen A, Hansen J, et al. Working time characteristics and long-term sickness absence: a large register-based study of Danish and Finnish nurses. *Int J Nurs Stud* 2020 doi: https://doi.org/10.1016/j.ijnurstu.2020.103639

13. Toften S, Pallesen S, Hrozanova M, et al. Validation of sleep stage classification using non-contact radar technology and machine learning (Somnofy®). *Sleep Medicine* 2020;75:54-61.

14. Baruch Y, Holtom BC. Survey response rate levels and trends in organizational research. *Human Relations* 2008;61:1139-60.

15. Pallesen S, Bjorvatn B, Nordhus IH, et al. A new scale for measuring insomnia: the Bergen Insomnia Scale. *Percept Mot Skills* 2008;107:691-706.

16. American Academy of Sleep M. International classification of sleep disorders, 3rd ed. 3rd ed. ed: Darien, IL: American Academy of Sleep Medicine 2014.

17. Waage S, Moen BE, Pallesen S, et al. Shift work disorder among oil rig workers in the North Sea. *Sleep* 2009;32(4):558-65. [published Online First: 2009/05/06]

18. Åhsberg E. Dimensions of fatigue in different working populations. *Scandinavian Journal of Psychology* 2000;31:231-41.

19. Di Milia L, Smith PA, Folkard S. A validation of the revised circadian type inventory in a working sample. *Personality and Individual Differences* 2005;39:1293-305.

20. Adan A, Archer SN, Hidalgo MP, et al. Circadian Typology: A Comprehensive Review. *Chronobiology International, 2012, Vol29(9), p1153-1175* 2012;29(9):1153-75. doi: 10.3109/07420528.2012.719971

For peer review only

21. Horne JA, Ostberg O. A self-assessment questionnaire to determine morningness-eveningness in human circadian rhythms. *International journal of chronobiology* 1976;4(2):97-110. [published Online First: 1976/01/01]

22. Adan A, Almirall H. Horne & Östberg morningness-eveningness questionnaire: A reduced scale. *Personality and Individual Differences* 1991;12(3):241-53. doi: https://doi.org/10.1016/0191-8869(91)90110-W

23. Schmalbach B, Zenger M, Tibubos AN, et al. Psychometric properties of two brief versions of the Hopkins Symptom Checklist: HSCL-5 and HSCL-10. *Assessment* 2021;28:617-31.

24. Brayfield AH, Rothe HF. An index of job satisfaction. *Journal of applied psychology* 1951;35:307.

25. Kinnunen U, Feldt T, Geurts S, et al. Types of work-family interface: Well-being correlates of negative and positive spillover between work and family. *Scandinavian Journal of Psychology* 2006;47:149-62.

26. Vedaa O, Harris A, Erevik EK, et al. Short rest between shifts (quick returns) and night work is associated with work-related accidents. *Int Arch Occup Environ Health* 2019 doi: 10.1007/s00420-019-01421-8 [published Online First: 2019/03/18]

27. Singh J, Verbeke W, Rhoads GK. Do organizational practices matter in role stress processes? A study of direct and moderating effects for marketing-oriented boundary spanners. *Journal of Marketing* 1996;60:69-86. doi: 10.2307/1251842

28. Schaufeli WB, Salanova M, González-Romá V, et al. The measurement of engagement and burnout: a confirmative analytic approach. *Journal of Happiness Studies* 2002;3:71– 92.

29. Eriksen HR, Ihlebæk C, Ursin H. A scoring system for subjective health complaints (SHC). *Scandinavian Journal of Public Health* 1999;27:63-72.

30. Sonnentag S, Fritz C. The Recovery Experience Questionnaire: development and validation of a measure for assessing recuperation and unwinding from work. *Journal of Occupational Health Psychology* 2007;12:204-21.

31. Johns MW. A new method for measuring daytime sleepiness: the Epworth sleepiness scale. *Sleep* 1991;14:540-45.

For peer review only

32. Pallesen S, Grønli J, Myhre K, et al. A pilot study of impulse radio ultra wideband radar technology as a new tool for sleep assessment. *Journal of Clinical Sleep Medicine* 2018;14:1249-54.

33. StataCorp. Stata Statistical Software: Release 14. College Station, TX: StataCorp LP 2015.

34. Enders CK. Applied missing data analysis. New York, NY, US: Guilford Press 2010.

35. Hensing G, Alexanderson K, Allebeck P, et al. How to measure sickness absence? Literature review and suggestion of five basic measures. *Scandinavian Journal of Public Health* 1998;26:133-44.

36. Kraemer HC, Wilson GT, Fairburn CG, et al. Mediators and moderators of treatment effects in randomized clinical trials. *Archives of General Psychiatry* 2002;59:877-83.

37. Orne MT. On the social psychology of the psychological experiment: With particular reference to demand characteristics and their implications. *American Psychologist* 1962;17:776–83.

38. Weiss NS. Analytic approaches for dealing with possible recall bias in case-control studies—reply. *American Journal of Epidemiology* 1995;141:299.

For peer review only

Table 1. Examples of a two-week cycle of rotating shift work with and without quick returns

	Week 1							Week 2						
	Monday	Tuesday	Wednesday	Thursday	Friday	Saturday	Sunday	Monday	Tuesday	Wednesday	Thursday	Friday	Saturday	Sunday
Scenario1: Rotating three-shift **with** quick returns	Day	Day	Night	Night				Evening	Day	Day		Evening	Day	Evening
Scenario1: Rotating three-shift **without** quick returns	Day	Day	Night	Night				Day	Day	Day		Evening	Evening	Evening
Scenario2: Rotating three-shift **with** quick returns	Evening	Day	Day		Night	Night	Night			Evening	Day	Day		
Scenario2: Rotating three-shift **without** quick returns	Day	Evening	Evening		Night	Night	Night			Day	Day	Day		
Scenario3: Weekend shift **with** quick returns	Evening	Day	Day		Evening	Day	Evening	Day		Day	Day			
Scenario3: Weekend shift **without** quick returns	Day	Day	Day		Day	Evening	Evening		Day	Day	Evening			
Scenario4: Rotating two-shift **with** quick returns		Day	Day	Evening	Day			Evening	Day	Evening		Day		
Scenario4: Rotating two-shift **without** quick returns	Evening		Day	Day	Day			Evening	Evening		Day	Day		

Note. Rotating three-shift refers to a shift schedule in which the workers alternates between day-, evening- and night shifts. Rotating two-shift refers to a shift schedule in which the workers alternates between only two of the shifts (e.g., only working day and evening shifts).

For peer review only

Table 2. Key measures and timing of assessment

	Baseline	Six-month follow-up
**Primary outcome**		
From hospital register		
Sickness absence	X	X
**Secondary outcomes**		
Self-reported questionnaires		
The Bergen Insomnia Scale (BIS)	X	X
Shift work disorder (SWD)	X	X
The Swedish Occupational Fatigue Inventory (SOFI)	X	X
The revised Circadian Type Inventory (rCTI)	X	
The Horne-Östberg Morningness Eveningness Questionnaire (MEQ)	X	
The Hopkins Symptom Checklist - 5 (HSCL- 5)	X	X
Job Satisfaction Index (JSI)	X	X
The Work-Family Interface Scale (WFIS)	X	X
Work-related negative incidents	X	X
The Turnover Intention Scale (TIS)	X	X
The Utrecht Work Engagement Scale (UWES - 9)	X	X
Subjective Health Complaints inventory (SHC) (three of five subscales)	X	X
Recovery Experience Questionnaire (REQ) (two of four dimensions)	X	X
Epworth Sleepiness Scale (ESS)	X	X
Sleep monitoring study (≈50)		
Sleep diary (≥7 days)	X	X
Xethru sensor (≥7 days)	X	X
**Additional measures**		
Self-reported questionnaires		
Unwanted/negative effects		X
Self-rostering	X	X
Experience of the implementation of the intervention		X
Physical activity	X	X
Commute time	X	
Sleep duration and perceived need for sleep	X	X
Use of sleep medication and light treatment	X	X
Satisfaction with work schedule	X	X
Preferred presence of quick return in work schedule	X	X
**Demographics and background information**		
From hospital register		
Sex	X	
Age	X	
Percentage of full-time equivalent	X	X
Payroll data	X	X
Self-reported questionnaires		

For peer review only

Marital status	X
Highest completed degree	X
Years of experience with shift work	X
Children living at home	X

For peer review only

Figure caption

Figure 1. Flow Diagram of timeline for recruitment, randomization, assessments and for undertaking primary and secondary analyses

For peer review only

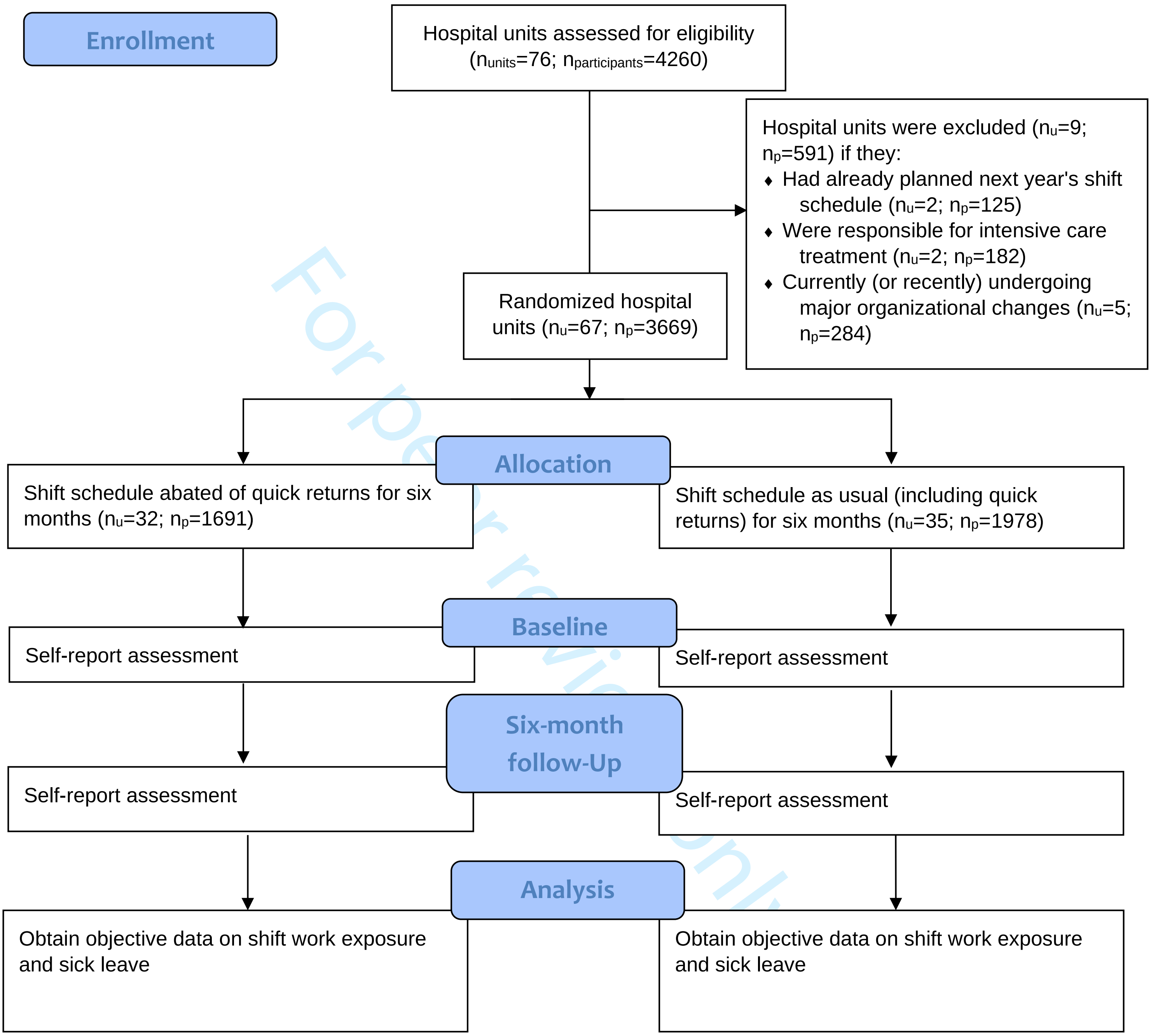

Figure 1. Flow Diagram of timeline for recruitment, randomization, assessments and for undertaking primary and secondary analyses

SPIRIT

STANDARD PROTOCOL ITEMS: RECOMMENDATIONS FOR INTERVENTIONAL TRIALS

SPIRIT 2013 Checklist: Recommended items to address in a clinical trial protocol and related documents*

Section/item	ItemNo	Description	Page or section on which item is reported
**Administrative information**			
Title	1	Descriptive title identifying the study design, population, interventions, and, if applicable, trial acronym	1 (front/cover page)
Trial registration	2a	Trial identifier and registry name. If not yet registered, name of intended registry	2 and 8
	2b	All items from the World Health Organization Trial Registration Data Set	Included in a separate document with the submission
Protocol version	3	Date and version identifier	1 (front/cover page)
Funding	4	Sources and types of financial, material, and other support	24
Roles and responsibilities	5a	Names, affiliations, and roles of protocol contributors	1 (front/cover page) and Author statement on page 24
	5b	Name and contact information for the trial sponsor	24

For peer review only

	5c	Role of study sponsor and funders, if any, in study design; collection, management, analysis, and interpretation of data; writing of the report; and the decision to submit the report for publication, including whether they will have ultimate authority over any of these activities	24
	5d	Composition, roles, and responsibilities of the coordinating centre, steering committee, endpoint adjudication committee, data management team, and other individuals or groups overseeing the trial, if applicable (see Item 21a for data monitoring committee)	NA
**Introduction**			
Background and rationale	6a	Description of research question and justification for undertaking the trial, including summary of relevant studies (published and unpublished) examining benefits and harms for each intervention	See introduction from page 4
	6b	Explanation for choice of comparators	See Methods from page 8
Objectives	7	Specific objectives or hypotheses	See aims on page 7
Trial design	8	Description of trial design including type of trial (eg, parallel group, crossover, factorial, single group), allocation ratio, and framework (eg, superiority, equivalence, noninferiority, exploratory)	See Methods from page 8
**Methods: Participants, interventions, and outcomes**			
Study setting	9	Description of study settings (eg, community clinic, academic hospital) and list of countries where data will be collected. Reference to where list of study sites can be obtained	See Methods from page 8

For peer review only

Eligibility criteria	10	Inclusion and exclusion criteria for participants. If applicable, eligibility criteria for study centres and individuals who will perform the interventions (eg, surgeons, psychotherapists)	See Participants and procedure from page 9
Interventions	11a	Interventions for each group with sufficient detail to allow replication, including how and when they will be administered	See Intervention from page 11
	11b	Criteria for discontinuing or modifying allocated interventions for a given trial participant (eg, drug dose change in response to harms, participant request, or improving/worsening disease)	NA
	11c	Strategies to improve adherence to intervention protocols, and any procedures for monitoring adherence (eg, drug tablet return, laboratory tests)	See Methods from page 8
	11d	Relevant concomitant care and interventions that are permitted or prohibited during the trial	NA
Outcomes	12	Primary, secondary, and other outcomes, including the specific measurement variable (eg, systolic blood pressure), analysis metric (eg, change from baseline, final value, time to event), method of aggregation (eg, median, proportion), and time point for each outcome. Explanation of the clinical relevance of chosen efficacy and harm outcomes is strongly recommended	See Assessments from page 12
Participant timeline	13	Time schedule of enrolment, interventions (including any run-ins and washouts), assessments, and visits for participants. A schematic diagram is highly recommended (see Figure)	See Methods from page 8 and Figure 1
Sample size	14	Estimated number of participants needed to achieve study objectives and how it was determined, including clinical and statistical assumptions supporting any sample size calculations	See Sample size from page 17
Recruitment	15	Strategies for achieving adequate participant enrolment to reach target sample size	See Methods from page 8

Methods: Assignment of interventions (for controlled trials)

Allocation:			
Sequence generation	16a	Method of generating the allocation sequence (eg, computer-generated random numbers), and list of any factors for stratification. To reduce predictability of a random sequence, details of any planned restriction (eg, blocking) should be provided in a separate document that is unavailable to those who enrol participants or assign interventions	See Methods from page 8
Allocation concealment mechanism	16b	Mechanism of implementing the allocation sequence (eg, central telephone; sequentially numbered, opaque, sealed envelopes), describing any steps to conceal the sequence until interventions are assigned	See Methods from page 8 and Randomisation from page 11
Implementation	16c	Who will generate the allocation sequence, who will enrol participants, and who will assign participants to interventions	See Methods from page 8 and Randomisation from page 11
Blinding (masking)	17a	Who will be blinded after assignment to interventions (eg, trial participants, care providers, outcome assessors, data analysts), and how	See Methods from page 8 and Randomisation and masking from page 11
	17b	If blinded, circumstances under which unblinding is permissible, and procedure for revealing a participant's allocated intervention during the trial	See Methods from page 8 and Randomisation and masking from page 11

Methods: Data collection, management, and analysis

For peer review only - http://bmjopen.bmj.com/site/about/guidelines.xhtml

Data collection methods	18a	Plans for assessment and collection of outcome, baseline, and other trial data, including any related processes to promote data quality (eg, duplicate measurements, training of assessors) and a description of study instruments (eg, questionnaires, laboratory tests) along with their reliability and validity, if known. Reference to where data collection forms can be found, if not in the protocol	See Methods from page 8
	18b	Plans to promote participant retention and complete follow-up, including list of any outcome data to be collected for participants who discontinue or deviate from intervention protocols	We use registry data for the primary outcome. See Methods from page 8
Data management	19	Plans for data entry, coding, security, and storage, including any related processes to promote data quality (eg, double data entry; range checks for data values). Reference to where details of data management procedures can be found, if not in the protocol	See Methods from page 8
Statistical methods	20a	Statistical methods for analysing primary and secondary outcomes. Reference to where other details of the statistical analysis plan can be found, if not in the protocol	See Data analysis plan from page 18
	20b	Methods for any additional analyses (eg, subgroup and adjusted analyses)	See Data analysis plan from page 18
	20c	Definition of analysis population relating to protocol non-adherence (eg, as randomised analysis), and any statistical methods to handle missing data (eg, multiple imputation)	See Data analysis plan from page 18
**Methods: Monitoring**			
Data monitoring	21a	Composition of data monitoring committee (DMC); summary of its role and reporting structure; statement of whether it is independent from the sponsor and competing interests; and reference to where further details about its charter can be found, if not in the protocol. Alternatively, an explanation of why a DMC is not needed	There is no data monitoring committee beyond the research group.

	21b	Description of any interim analyses and stopping guidelines, including who will have access to these interim results and make the final decision to terminate the trial	NA
Harms	22	Plans for collecting, assessing, reporting, and managing solicited and spontaneously reported adverse events and other unintended effects of trial interventions or trial conduct	See page 16, Additional measures of unwanted/negative effects
Auditing	23	Frequency and procedures for auditing trial conduct, if any, and whether the process will be independent from investigators and the sponsor	The shifts schedule is prepared in advance for the entire intervention period, and there will be no changes to the schedule during the intervention period, with the exception of necessary short-notice shift swaps, as described on page 12. This limits the need for auditing.
**Ethics and dissemination**			
Research ethics approval	24	Plans for seeking research ethics committee/institutional review board (REC/IRB) approval	See Ethics form page 20

For peer review only - http://bmjopen.bmj.com/site/about/guidelines.xhtml

Protocol amendments	25	Plans for communicating important protocol modifications (eg, changes to eligibility criteria, outcomes, analyses) to relevant parties (eg, investigators, REC/IRBs, trial participants, trial registries, journals, regulators)	NA
Consent or assent	26a	Who will obtain informed consent or assent from potential trial participants or authorised surrogates, and how (see Item 32)	See Ethics form page 20
	26b	Additional consent provisions for collection and use of participant data and biological specimens in ancillary studies, if applicable	NA
Confidentiality	27	How personal information about potential and enrolled participants will be collected, shared, and maintained in order to protect confidentiality before, during, and after the trial	See Ethics and dissemination from page 20
Declaration of interests	28	Financial and other competing interests for principal investigators for the overall trial and each study site	Page 24
Access to data	29	Statement of who will have access to the final trial dataset, and disclosure of contractual agreements that limit such access for investigators	See Data statement on page 24
Ancillary and post-trial care	30	Provisions, if any, for ancillary and post-trial care, and for compensation to those who suffer harm from trial participation	NA
Dissemination policy	31a	Plans for investigators and sponsor to communicate trial results to participants, healthcare professionals, the public, and other relevant groups (eg, via publication, reporting in results databases, or other data sharing arrangements), including any publication restrictions	See Stakeholder and public involvement and Ethics and dissemination from page 20

For peer review only - http://bmjopen.bmj.com/site/about/guidelines.xhtml

	31b	Authorship eligibility guidelines and any intended use of professional writers	The Vancouver recommendations apply to authorship
	31c	Plans, if any, for granting public access to the full protocol, participant-level dataset, and statistical code	See Data statement on page 24
**Appendices**			
Informed consent materials	32	Model consent form and other related documentation given to participants and authorised surrogates	The consent form used is based on a template from the Regional Committee for Medical and Health Research Ethics in Norway. We do not consider it necessary to publish this with the protocol paper.
Biological specimens	33	Plans for collection, laboratory evaluation, and storage of biological specimens for genetic or molecular analysis in the current trial and for future use in ancillary studies, if applicable	NA

*It is strongly recommended that this checklist be read in conjunction with the SPIRIT 2013 Explanation & Elaboration for important clarification on the items. Amendments to the protocol should be tracked and dated. The SPIRIT checklist is copyrighted by the SPIRIT Group under the Creative Commons "Attribution-NonCommercial-NoDerivs 3.0 Unported" license.

For peer review only

Items from the World Health Organization Trial Registration Data Set

**Data category**	**Information**
Primary registry and trial identifying number	ClinicalTrials.gov
	NCT04693182
Date of registration in primary registry	Des, 2020
Secondary identifying numbers	N/A
Source(s) of monetary or material support	The Research Council of Norway (303671) and the University of Bergen, Bergen, Norway
Primary sponsor	The Research Council of Norway (303671)
Secondary sponsor(s)	University of Bergen, Bergen, Norway
Contact for public queries	Øystein Vedaa, PhD [Oystein.Vedaa@fhi.no]
Contact for scientific queries	Øystein Vedaa, PhD [Oystein.Vedaa@fhi.no]
	Department of Health Promotion, Norwegian Institute of Public Health, Bergen, Norway
Public title	Health promoting work schedules: Protocol for a large-scale cluster randomized controlled trial on the effects of a work schedule without quick returns on sickness absence among healthcare workers
Scientific title	Health promoting work schedules: Protocol for a large-scale cluster randomized controlled trial on the effects of a work schedule without quick returns on sickness absence among healthcare workers
Countries of recruitment	Norway
Health condition(s) or problem(s) studied	Shift work, sickness absence, health and sleep
Intervention(s)	Active comparator: a work schedule without quick returns for six months
	Placebo comparator: a work schedule with quick returns for six months
Key inclusion and exclusion criteria	Ages eligible for study: ≥18 years
	Sexes eligible for study: both
	Accepts healthy volunteers: yes
	Inclusion criteria: the unit-level inclusion criteria are that the units should have 1) healthcare workers (other than physicians) who work rotating shifts, 2) employees who regularly have quick returns in their work schedule, and 3) a new shift rotation year commencing from the first half of 2021 (which is the case for most units at the included hospitals)
	Exclusion criteria: exclusion criteria at the unit-level are 1) units recently (or will in the near future) went through other major organizational changes that may confound the results of the trial (this includes during the period from one year before the intervention starts until the intervention period is over), or 2) unit's manager or a substantial number of employees strongly oppose participation

For peer review only

Study type	Interventional
	Allocation: cluster randomized intervention model. It is not possible to blind the intervention for the participants, but the statistician who carries out the analyzes will be blinded to group allocation.
	Primary purpose: prevention
Date of first enrolment	January 2021
Target sample size	3669
Recruitment status	Recruiting
Primary outcome(s)	Sickness absence data will be retrieved from the local records kept by the hospital.
Key secondary outcomes	Questionnaire data: Insomnia, Shift work disorder, Occupational Fatigue, Psychological distress, Job Satisfaction, Work–family spillover, Work-related negative incidents, Turnover Intention, Work Engagement, Subjective Health Complaints, Recovery Experience, Sleepiness, and Sleep

For peer review only

Health promoting work schedules: Protocol for a large-scale cluster randomized controlled trial on the effects of a work schedule without quick returns on sickness absence among healthcare workers

Øystein Vedaa[1,2,3], Ingebjørg Louise Rockwell Djupedal[4], Erling Svensen[5], Siri Waage[6,7], Bjørn Bjorvatn[6,7], Ståle Pallesen[4,7,8], Stein Atle Lie[9], Morten Birkeland Nielsen[4,10], Anette Harris[4]

[1] Department of Health Promotion, Norwegian Institute of Public Health, Bergen, Norway;

[2] Department of Mental Health, Norwegian University of Science and Technology, Trondheim, Norway;

[3] Department of Research and Development, St Olavs University Hospital, Trondheim, Norway

[4] Department of Psychosocial Science, University of Bergen, Bergen, Norway;

[5] Haukeland University Hospital, Bergen, Norway;

[6] Department of Global Public Health and Primary Care, University of Bergen, Bergen, Norway;

[7] Norwegian Competence Center for Sleep Disorders, Haukeland University Hospital, Bergen, Norway;

[8] Optentia at the Vaal Triangle Campus of the North-West University, South-Africa;

[9] Department of Clinical Dentistry, University of Bergen, Bergen, Norway;

[10] National Institute of Occupational Health, Oslo, Norway

Correspondence to: Øystein Vedaa, Department of Health Promotion, Norwegian Institute of Public Health, Zander Kaaes gate 7, 5018 Bergen, Norway; e-mail: Oystein.Vedaa@fhi.no

Word count: 5975

Date and version identifier:

- Issue date: 14 Jan 2022
- Protocol amendment number: 02

For peer review only

Abstract

Introduction In shift work, quick returns refer to transitions between two shifts with less than 11 hours available rest time. Twenty-three per cent of employees in European countries reported having quick returns. Quick returns are related to short sleep duration, fatigue, sleepiness, work-related accidents, and sickness absence. The present study is the first randomized controlled trial (RCT) to investigate the effect of a work schedule without quick returns for six months, compared to a work schedule that maintains quick returns during the same time frame.

Methods and analysis A parallel-group cluster RCT in a target sample of about 4000 healthcare workers at Haukeland University Hospital in Norway will be conducted. About 70 hospital units will be randomized to a work schedule without quick returns for six months or continue with a schedule that maintains quick returns. The primary outcome is objective records of sickness absence; secondary outcomes are questionnaire data (n ≈ 4000 invited) on sleep and functioning, physical and psychological health, work-related accidents, and turnover intention. For a subsample, sleep diaries and objective sleep registrations with radar technology (n ≈ 50) will be collected.

Ethics and dissemination The study protocol was approved by the Regional Committee for Medical and Health Research Ethics in Western Norway (2020/200386). Findings from the trial will be disseminated in peer-reviewed journals and presented at national and international conferences. Exploratory analyses of potential mediators and moderators will be reported. User-friendly outputs will be disseminated to relevant stakeholders, unions and other relevant societal groups.

Trial registration number NCT04693182; Pre-recruitment.

Key words: Quick returns, Shift work, Sickness absence, Sick leave

For peer review only

Article summary:

Strength and limitations of the study:

- This is the first randomized controlled trial to investigate the effect of a work schedule without quick returns.
- The primary outcome measure is objective register data on sickness absence with no missing data.
- As this is an evaluation of an organizational quality improvement measure implemented for all employees at the hospital, we get to study the effect on the entire target population with full representativeness.
- One concern in this trial is how well the intervention group will succeed in abolishing quick returns from the shift schedule (given that this is a study conducted in a naturalistic setting).
- Another concern in this trial is that a shift schedule that does not include quick returns may unintentionally include other unfavourable shift characteristics that could potentially confound the results (e.g., more consecutive evening shifts).

For peer review only

INTRODUCTION

An important principle when planning shift schedules is that employees are apportioned sufficient time to rest and recover between shifts. According to the EU's Working Time Directive (2003/88 / EC),[1] employees are entitled to minimum 11 hours of rest between two consecutive shifts. Still, in some countries, including Norway, employers and the employees' representatives can agree on rest periods less than 11 hours between two shifts. In this realm, the term *quick return* refers to transitions between two shifts with less than 11 hours available rest time. Quick returns occur most often between an evening shift and a day shift the following day, but can also occur between a night shift and an evening shift, and between a day shift and a night shift the subsequent night.[2] In the sixth European Working Conditions Survey published in 2016,[3] 23 per cent of employees in European countries reported having at least one quick return during the last month. Quick returns seem to be particularly prevalent in the healthcare sector. In a large Danish register survey (n = 69,200), it was shown that, on average per year, 65 per cent of nurses, 38 per cent of physicians, and 26 per cent of medical secretaries had quick returns in their work schedule.[4]

Eleven hours define the upper limit of potential time for rest between two shifts in a quick return, while the actual time available is often substantially shorter. A Norwegian study investigating payroll data from nurses found that almost 2/3 of the quick returns involved rest time less than 9 hours between two shifts, and some employees (2%) even had rest time of less than 7 hours.[5] The time available for sleep and recuperation is further curtailed by the time it takes to commute to and from work, time for self-care, meals, family obligations and house chores. A systematic literature review reported that sleep duration in quick returns between evening and day shifts typically is reduced to 5-6.5 hours, compared to 7-8 hours on non-quick return nights.[2] In addition to reduced sleep duration, the most robust findings in the literature review were that quick returns were associated with more fatigue, higher levels of

For peer review only

sleepiness, and shift work disorder (i.e., sleep problems or sleepiness related to a recurring shift schedule). Individual studies also showed that quick returns were associated with poorer sleep quality, impaired general health and well-being, higher self-reported stress, and lower job satisfaction.[2]

The most immediate consequence of quick returns is probably shortened sleep.[6] It is reasonable to think that this in turn leads to a number of other negative consequences. In a diary study (sleep- and work-schedule), we found that nurses reported higher sleepiness during the day shift when they had quick return to the day shift, as compared to during other regular day shifts.[6] In fact, the results showed that the nurses were as sleepy during the day shift after a quick return as they were during night shifts. It is conceivable that high sleepiness represents a greater problem when it occurs during day shifts than during night shifts, since day shifts are often busier[7] and typically experienced as more stressful.[6] The combination of a high level of sleepiness during a stressful shift might represent a type of circumstance that increases the risk of accidents. Indeed, the association between quick returns and work-related accidents or injuries is established in previous research. In a large register-based study from Denmark, researchers linked payroll data of healthcare workers with national registers of injuries. The results showed that quick returns were associated with a 39 per cent higher risk of injury, compared with having 15-17 hours off between two shifts.[4] A longitudinal study found an increased risk of needlestick injuries among nurses who reported having quick returns as compared to nurses without quick returns.[8] A study based on cross-sectional data found that quick returns were associated with an increased risk of falling asleep at work, of experiencing work-related injuries to themselves, of injuring patients or others, and of damaging equipment at work.[9] In fact, the risk of experiencing injuries to themselves and damaging equipment at work was greater with quick returns than with night shifts. Another longitudinal study, partly based on the same data, demonstrated that nurses who experienced

For peer review only

an increase in the number of quick returns over time also had an increased risk of work-related accidents, whereas a decrease in the number of quick returns over time was associated with reduced risk of accidents.[10]

Over the past five years, researchers have increasingly begun to use register/payroll data on exposure to shift work when examining the consequences of different shift characteristics. These data are registered by the employees, typically at healthcare institutions and include information about the date and start and stop time for all shifts performed. In some cases, it is also possible to retrieve data on sickness absence from the same registers. These data comprise information on the date of each day of absence (self-certified and medically certified absence) due to illness. In a Finnish study using such register data from healthcare workers, the relationship between quick returns and short-term sick leave (1 to 3 days) was investigated. The results showed that having few quick returns (defined as 3 or fewer over a period of 28 days) was associated with a lower risk of short-term sick leave, while having many quick returns (5 or more over a period of 28 days) was associated with a higher risk of short-term sickness absence, compared to having no quick returns.[11] In a study based on Danish and Finnish register data, it was found that healthcare workers who had at least 13 quick returns during a year had a higher risk of long-term sick leave than those with fewer quick returns.[12] These findings are in line with results using corresponding register data in Norway.[5] In one study, the findings showed that exposure to quick returns one month was associated with a higher risk of sick leave the following month. On average, nurses had 3 quick returns per month, which corresponded to 21 per cent more sickness absence days the subsequent month (over and above the sickness absence days of workers without quick return).[5]

Research on quick return and health and safety related outcomes have so far all been based on correlational studies. We do not yet know whether these health outcomes are caused by

For peer review only

exposure to quick returns. The present study is the first randomized controlled trial (RCT) conducted to determine the effects of abolishing quick return from the work schedule.

Aims

This paper describes the protocol for a two-arm cluster randomized controlled trial that assesses the consequences of a shift work schedule abolishing quick returns, compared to a schedule maintaining quick returns for a six months period. First, we will examine any differential change in sickness absence (primary outcome) during the six-month intervention period. Second, we will examine if there are differential changes in sleep and functioning, physical and mental health, work-related accidents, and turnover intention, among others (secondary outcomes). Third, we will investigate if individual characteristics associated with shift work tolerance including sex, age, personality and subjectively reported sleep need moderate the negative effects of quick returns on the primary and secondary outcomes. Finally, the study will investigate if individual factors like satisfaction with work schedule, job satisfaction, job engagement and work-family interference moderate the negative effects of quick returns on the primary and secondary outcomes.

For peer review only

METHODS AND ANALYSIS

The protocol for the current trial follows the SPIRIT checklist for intervention trials. The trial is further pre-registered with the Clinical Trials website (ClinicalTrials.gov identified: NCT04693182). The checklist for the current trial is available as online supplementary file 1.

Figure 1 shows the Flow Diagram for the current trial. The flow chart illustrates the timeline for recruitment, randomization, assessments and for undertaking primary and secondary analyses.

Insert Figure 1 about here

Research design

A cluster randomized controlled trial comparing a six months work schedule abolishing quick returns (intervention) with that of a six months work schedule maintaining a normal amount of quick returns (control) will be conducted. The clusters in this trial represent hospital units that are randomly selected to receive (or not receive) the intervention. 'Normal amount of quick returns' refer to that which is the common practice at the respective hospital unit in recent years (i.e., when no explicit changes have been made to the work schedule), which means that the total number of quick returns at the unit will vary from 329–2356 per year (on average, nurses have three quick returns per month at this hospital[5]). In September 2020, the hospital units were informed about the conditions they would be randomized to at the start of the study in 2021. Thus, the autumn of 2020 was spent planning the shift schedule for 2021 (i.e., removing quick returns for the intervention group and maintaining quick returns for the control group). Most hospital units started the intervention period in the first half of 2021,

For peer review only

while some units started the intervention period in the second half of 2021.The intervention period in this study is six calendar months.

The primary outcome is sickness absence retrieved from the local registers kept by the hospital (including short- and long-term sick leave). The baseline measurements will be sickness absence from the year preceding the intervention, which for each individual participant will be matched on duration and season to that of the intervention period. We will apply for ethical approval to use the register data from all employees at the randomised hospital units without obtaining individual consent. In addition, a consent-based part of the trial will be conducted, in which secondary outcome measures will be collected via questionnaire at baseline and six-month follow-up. All employees (n ≈ 4000) at the randomized units will be asked to complete a digital questionnaire. This will be made available to the employees when they log on to enter their working hours ("MinGat"). Baseline assessment will occur prior to the intervention period, and follow-up assessment will occur towards the end of the intervention period. A subsample (n ≈ 50) will be asked to record their sleep with advanced sleep radar technology (Somnofy™)[13] and subjectively with sleep diaries for ≥1 week at the baseline and follow-up assessments, respectively.

Participants and procedure

Recruitment

This trial is carried out in collaboration with the human resources department at Haukeland University Hospital, Bergen, Norway. All hospital care units that have 24-hour staffing at Haukeland University Hospital will be considered for inclusion in this trial. This will include all healthcare workers working shifts, except for physicians. Physicians are to be excluded since they often have a different shift schedule and compensation scheme compared to other occupational groups at the hospital. Hereinafter, 'all employees' refer to all healthcare workers

For peer review only

engaged in shift work at the randomised hospital units, except for physicians. All employees (n ≈ 4000) at the randomized hospital units will be asked to complete a questionnaire prior to, and at the end of, the intervention period. Recruitment for this part of the trial will take place via the hospital's internal website or through the site in which the employees enter their working hours ("MinGat"). Researchers (the authors of this paper) and human resources personnel at the hospital will attend staff meetings at all included units to inform about the research project and encourage participation. A subsample of n ≈ 50 employees (evenly distributed from the intervention and the control units) will be recruited by convenience for the objective sleep monitoring section of the trial.

Eligibility

The unit-level inclusion criteria are that the units should have 1) healthcare workers (other than physicians) who work rotating shifts, 2) employees who regularly have quick returns in their work schedule, and 3) a new shift rotation year commencing from the first half of 2021 (which is the case for most units at the included hospitals). Exclusion criteria at the unit-level are 1) units recently (or will in the near future) went through other major organizational changes that may confound the results of the trial (this includes during the period from one year before the intervention starts until the intervention period is over), or 2) unit's manager or a substantial number of employees strongly oppose participation. Haukeland University Hospital had a total of 76 units which were considered for eligibility, 67 of which were deemed eligible for the trial. **Figure 1** provides an overview of the number of units excluded before the randomization took place.

This trial consists of three different data collections with an expected dissimilar number of participants: A) a register study, i.e. the primary investigation, in which we expect no missing data, B) a questionnaire study, i.e. the secondary investigation, with an expected response rate of 40-50 per cent,[14] and C) the sleep monitoring study, i.e., secondary investigation,

For peer review only

conducted on a subsample of ≈50 employees recruited by convenience. All employees from the randomised hospital units working ≥80 percent of full-time equivalent will participate in the register-based study (investigation A) and the same group will be asked to participate in the questionnaire-based study (investigation B). Finally, participants in the sleep monitoring study (investigation C) will be recruited by convenience from the same sample of healthcare workers requiring that they are working ≥80 percent of full-time equivalent.

Randomisation and masking

The randomization in this trial occurred at the cluster level, in which hospital units constituted the clusters. As shown in Figure 1, a total of 67 hospital units were randomized. Hospital units can vary in terms of how much staff they need over the 24-hour day, hence, the work schedule and the occurrence of, for example, quick returns and night shifts can vary across the units. Similar units were therefore grouped together based on the fact that they shared some attributes or characteristics. Then a stratified randomization was performed to the two study conditions in a 1: 1 ratio. One subgroup could, for example, consist of units with emergency functions, another with intensive care functions, one with mental health care, and one with maternity care, etc. In total we had 10 strata and the sizes of each stratum varied between 2 and 19 hospital units. The randomization list for each stratum was generated by the online randomization webpage, www.randomization.com, and the list for each stratum was saved.

It is not possible for participants to be blinded to the group to which they are assigned. However, statistical analyses will be done by a researcher who is masked to group allocation.

Intervention

The intervention entails implementing a shift schedule which abolishes quick returns for a six-month intervention period. The mean number of quick returns in the various hospital units in this trial varies from 3–32 per year. The intervention means that this number is abolished or

For peer review only

reduced as much as possible. For practical reasons the intervention may be a matter of reducing rather than completely abolishing quick returns. This might be in the case of ensuring adequate staffing (e.g., due to sickness absence), and since employees for various reasons may make short-notice shift swaps in which it is not possible to comply with the rule of avoiding quick returns. The human resources department at the hospital will assist shift planners in identifying appropriate shift schedules that do not include quick returns. **Table 1** shows some of the examples that were used to show shift planners how this could be done.

The control condition in this trial implies that employees maintain the same number of quick returns as in previous years for the six-month intervention period. It is important to note that hospital units in the control group are not expected to experience any increase in the number of quick returns.

Insert Table 1 about here

Assessments

All assessments/instruments in this trial are described below. **Table 2** provides an overview of the source and timing of the assessments. The primary outcome in this trial is sickness absence (number of days or spells). We will compare the sickness absence in intervention group with the control group during the intervention period, while adjusting for previous sick leave from the corresponding period the year preceding the intervention (matched on duration and season). Other measures included in this trial are secondary outcomes or outcomes used in exploratory or subsidiary analyses.

Demographics

For peer review only - http://bmjopen.bmj.com/site/about/guidelines.xhtml

Demographic information will be obtained both from the register at the hospital as well as from a questionnaire. Information on sex, age and percentage of full-time equivalent will be available from the register data; while information on marital status, highest completed education/degree, years of experience with shift work, and if the participant has children living at home will be collected through the questionnaire.

Primary outcome

Sickness absence data will be retrieved from the local records kept by the hospital.[5] This record includes information about the date of any absence of the individual employee, implying that it includes information about both short- and long-term sickness absence. Further, these data include information on whether the absence is self-certified or whether it is certified by a physician, whether the absence is due to a sick child of whom the employee has childcare responsibility of, and whether the absence is due to COVID-19 related issues (e.g., quarantine).

Secondary outcomes

The Bergen Insomnia Scale (BIS)[15] will be used to measure sleep problems among participants. The scale originally comprised six items that assess symptoms of insomnia. An additional item will be included to the scale in which we will ask about the duration of any sleep problems. This makes it possible to define insomnia according to the diagnostic criteria in the International Classification of Sleep Disorders-Third Edition,[16] Diagnostic and Statistical Manual of Mental Disorders-Fifth Edition, and the International Classification of Diseases-11th Revision.

Shift work disorder (SWD) will be measured with three standardised questions.[17] SWD was evaluated with three questions based on the criteria from the third edition of the International Classification of Sleep Disorders (ICSD-3).[16] The questions were: a) Do you have a work

For peer review only

schedule that sometimes overlap with the time you usually sleep?, b) if yes, does this cause insomnia and/or excessive sleepiness due to reduced amount of sleep?, c) if yes, has this lasted for at least three months? Participants will be classified as having SWD when responding "yes" to all three questions.

The revised Swedish Occupational Fatigue Inventory (SOFI) will be used to measure lack of energy, physical exertion, physical discomfort, lack of motivation and sleepiness.[18] Participants are asked to indicate the extent to which they have recently (or for a specified period of time) experienced a list of 20 psychological and physical sensations related to fatigue.

The revised Circadian Type Inventory (rCTI) comprises 11 items, five of which assesses flexibility and six assesses languidity.[19] High scores on flexibility reflect better ability to sleep and work at odd times, whereas high scores on languidity indicate difficulties overcoming drowsiness and feelings of lethargy following sleep loss.

The Horne-Östberg Morningness Eveningness Questionnaire (MEQ) is the most widely used morningness-eveningness inventory,[20] and is designed to determine preferred timing of sleep and activities during the 24-hour day.[21] The MEQ reduced version (rMEQ) will be used in the present trial, which is comprised of five items from the original scale.[22]

Hopkins Symptoms Checklist - 5 (HSCL-5) will be used to measure general psychological distress.[23] HSCL-5 includes five questions about nervousness or inner turmoil, fear or feeling anxious, feeling hopeless about the future, depression or melancholy, worry or restlessness. An average score can be calculated across the five items with values that vary from 1 to 4, in which higher scores indicate a higher degree of psychological distress. The composite score is sometimes recoded into a two-part variable in which a score higher than 2.00 is defined as a high score.

For peer review only

Job Satisfaction Index (JSI) comprise five items measuring satisfaction with work (e.g., "I find real enjoyment in my work").[24] Each item is answered on a 5-point Likert scale, ranging from 1 (strongly disagree) to 5 (strongly agree). Higher scores reflect higher levels of overall job satisfaction.

The Work Family Interface Scale[25] will be used to evaluate the four types of work–family spillover. Consisting of 14 items, the scale was designed to measure both negative and positive work–to– family (NWFS and PWFS) and family–to–work spillover (NFWS and PFWS). The responses were graded by a frequency based on a 1–5 Likert scale, with alternatives ranging from never to very often.

Work-related negative incidents will be assessed using eight items measuring the number of self-reported work-related accidents, near accidents and dozing off at work or while driving to or from work. These questions have been developed in connection with the Norwegian Survey of Shift work, Sleep and Health among Nurses (SUSSH), and have been used in several previous publications.[26]

The Turnover Intention Scale (TIS) will be used to measure turnover intention, which is comprised of three items adapted from Michigan Organizational Assessment Questionnaire.[27] The three items are: "I will actively look for a new job in the next year," "I often think about quitting," and "I will probably look for a new job by the next year." Responses were recorded on a 5-point Likert scale from 1 (strongly disagree) to 5 (strongly agree), yielding a score range of 3–15. A high score indicates a high degree of turnover intention.

The Utrecht Work Engagement Scale (UWES - 9) will be used to measure work engagement.[28] The UWES is originally comprised of 17 items rated on a 7-point scale ranging from "never" (0) to "always/every day" (6). The 9-item version of the UWES includes three items for each of the three factors; Vigor (e.g., "At my job, I feel strong and vigorous"),

For peer review only

Dedication (e.g., “I am enthusiastic about my job”), and Absorption (e.g., “When I am working, I forget everything else around me”). A higher score indicates more work engagement.

Subjective Health Complaints inventory (SHC)[29] consists of a list of 29 common health complaints that participants grade the intensity of which they experience each complaint on a four-point scale (0 = not at all; 1 = a little; 2 = some; 3 = severe). In this study, we include three of the five subscales; i.e. musculoskeletal complaints, pseudoneurological complaints, and gastrointestinal complaints.

Recovery Experience Questionnaire (REQ)[30] will be used to measure recovery experiences. REQ is originally a 16-item questionnaire with the four subscales psychological detachment, relaxation, mastery, and control. The present study includes the subscales of psychological detachment and relaxation. Each item is scored on a 5-point Likert scale ranging from 1 (strongly disagree) to 5 (strongly agree) of which a higher score indicates better detachment/relaxation.

Epworth Sleepiness Scale (ESS)[31] will be used to measure participants sleepiness. ESS is an eight-item questionnaire asking the participants how likely they are to doze off or fall asleep in different situations of everyday life including (e.g. while sitting and reading, watching TV, when sitting and talking to someone, etc.). For each item, participants report the chance of dozing as never (0), slight (1), moderate (2), or high (3) (total score range between 0 – 24). A higher score indicates higher level of sleepiness.

Additional measures of unwanted/negative effects and other exploratory analyses

Other factors that may have an impact on how the employees react to the intervention will also be investigated. The participants' attitudes to the intervention and the research project will be measured, in addition to how they experience the implementation of the intervention.

For peer review only

A set of questions measuring possible negative or unwanted effects of the intervention will be developed for the purpose of this trial. These questions will specifically ask if the changed work schedule has led to disturbed sleep, more stress, worry, depression, overall less time for recovery between work periods, problems in work-family balance, disrupted social relationships, poorer psychosocial climate at work, experience of reduced quality of care offered to patients, etc. For some employees, it is possible that a work schedule that does not allow for quick returns represents a restricted opportunity to co-design their schedule (i.e., self-rostering) and reduces the duration of free periods. Therefore, we will measure the participants' perceived change in relation to these parameters. Furthermore, we will include questions about satisfaction with work schedule, commute time, habitual and preferred sleep duration, current use of prescribed or over-the-counter sleep medication, current use of light treatment to improve sleep, and participants' physical activity level. Finally, the questionnaire will include an open text box in which participants can write freely, for example about anything they would like to convey related to the intervention (e.g. topics/themes they felt was inadequately addressed in the survey).

Sleep will be assessed more thoroughly for a subsample of ≈50 employees. The measures of sleep will include daily self-rating of sleep-wake patterns reported using the consensus sleep diary,[17] as well as sleep measured objectively using the Xethru sensor, a low-powered ultra-wideband radar.[32] The sleep registration will occur for ≥7 days at baseline and at six-month follow-up.

Sample size

In this trial, all available hospital units at Haukeland University Hospital with healthcare workers who work rotating shifts were assessed for eligibility. This included 76 units and 4260 healthcare workers. As shown in Figure 1, a total of 67 of these units were finally included, i.e. 3669 healthcare workers. Based on previous published data[5] we have calculated

For peer review only

that a total of 2028 participants is sufficient to reveal a difference in days of sick leave of 0.9 and 1.25 with an ICC of 0.1 and an average size of the units of 52 (calculation made in: StataCorp. 2015).[33] With 67 hospital units and 3669 participants, we will thus be well within the number of participants required for the primary outcome variable and we consider this sufficient for all conceivable purposes of this trial.

Data analysis plan

All analyses will be conducted based on the intention-to-treat population, unless otherwise stated. To examine the effects of a shift schedule abated of quick returns on primary and secondary outcomes, the observed rates or scores will be analysed by means of latent growth models (or other equivalent models such as generalized linear mixed models). The observed rates or scores before and during the intervention period will be modelled by a random intercept and a fixed slope. The effect of the intervention will be estimated by using the group variable (intervention vs. control) as a predictor of the slope. Between-group effect sizes (Cohen's d) will be calculated by dividing the mean difference in estimated change in scores from baseline to the follow-up assessment by the pooled SD at baseline. Robust maximum likelihood will be used as the estimator, providing unbiased estimates under the assumption of data being missing at random,[34] which might be partly met through the inclusion of baseline scores to the model. The primary outcome measure in this trial is sickness absence data retrieved from the register at the hospital, in which we expect no missing data. However, it is reasonable to expect some missing data on the secondary outcome measures, as data are collected through questionnaire or via the sleep radar and sleep diary.

As some data for the follow-up questionnaire and sleep radar/diary assessment will be missing not at random, the robustness of the results under the missing-at-random assumption will be tested by sensitivity analyses in which the missing scores at follow-up will be replaced by baseline values for each respective individual. Since it is possible to imagine that some

For Peer review only

participants may experience worsening because of the intervention, we will consider carrying out more rigorous sensitivity analyses. For example, by replacing missing scores at the follow-up assessment with baseline scores multiplied by a given factor (higher or lower than 1.00 depending on the direction that indicates a worsening) in the intervention group and by 1.00 in the control group. These sensitivity analyses will only be performed on selected variables depending on the focus in the respective article.

The intention-to-treat analyses may be accompanied by selected per-protocol analyses in which we, based on payroll data, define a group that has completely abolished or had a satisfactory reduction in the number of quick returns during the intervention period.

The primary outcome of sick leave will mainly be analysed in terms of the total number of sickness absence days and periods (spells) for a given period *before* compared to *during* the intervention period.[5] The models of sickness absence will take into account the zero inflation in this type of data. Other operationalisations of sickness absence might also be considered in accordance with recommendations in the literature.[35] For a further investigation of the sickness absence data, we will consider the use of more complex survival analyses (e.g., Cox proportional hazards model), and we will also consider modelling time to return to work (from sickness absence) and/or time before taking sickness absence according to group allocation.

Since the introduction of a work schedule without quick returns may entail an alternative schedule with an increase in other undesirable characteristics (e.g., more consecutive evening shifts), we will consider conducting analyses that adjust for such characteristics.

Mediator and moderator analyses will be performed for exploratory purposes, based on the basic principle for such analyses in randomised controlled trials as described by others (e.g., [36]). For example, some of the data collected on demographics, sleep-related personality traits

For peer review only

(rCTI and MEQ), mental health, among others, can be used to examine factors that may moderate the impact of the intervention.

Stakeholder and public involvement

This trial is carried out in close collaboration with the HR department at Haukeland University Hospital. In addition, representatives from all relevant trade unions at the hospital will be involved in the planning and implementation of the research project. The findings of the trial will be disseminated via scholars in terms of scientific paper and conference presentations, and by stakeholder/union advocacy and other relevant public and community groups. Further, Haukeland University Hospital will arrange a conference for other relevant stakeholders, in which research results will be presented and the implications of the findings will be discussed.

Patient involvement

No patient involved.

Ethics and dissemination

The study protocol was approved by the Regional Committee for Medical and Health Research Ethics in Western Norway (2020/200386). In this trial, all employees at the included hospital units will be randomized to one of two conditions, and we will retrieve register data on working hours and sickness absence without collecting individual consent. This poses an ethical dilemma since all participation in research – especially when people are exposed to an intervention – should be consent-based. However, the intervention in this trial is to abolish or substantially reduce quick returns, and *not* to increase any exposure. This is thus considered not to represent a significant burden on the participants, as the presence of quick returns is

For peer review only

already a violation of the Working Environment Act. In addition, we expect that the intervention primarily will have beneficial effects on employees' health and safety. Abolishing or reducing the number of quick returns is a quality improvement measure that the Health Trust wants to implement independently of the present research project. The fact that the intervention is carried out as a research project is considered an advantage for the employees, as far as we are able to uncover any unintended negative effects of the intervention and further to be able to empirically document potential benefits on health and safety.

The result of this trial will potentially impact subsequent standards and practice when it comes to planning shift schedules and their compliance with the Working Environment Act. As vast number of employees might be affected by the trial results, it is equally important that the results are representative of the employees. We believe this justifies the use of the employees' register data without obtaining individual consent.

Participants will be required to provide informed consent before participating in the questionnaire and sleep diary/radar part of the trial. The recruitment and consent process emphasizes that participation is voluntary and that participants can withdraw from this part of the trial at any time point without any consequences. Self-report data are recorded in electronic files that are encrypted and password protected. No identifying information will be stored alongside the self-report data. Furthermore, only researchers directly involved in data analysis will be granted supervised access to de-identified participant data.

Findings from this randomized controlled trial will be disseminated in peer-reviewed publications and as conference presentations. After the research project is completed, Haukeland University Hospital will arrange a conference for stakeholders where the results and experience from the research will be disseminated and discussed.

For peer review only

DISCUSSION

To the best of our knowledge, this is the first randomized controlled trial to investigate the effect of a work schedule abolishing quick returns. Previous research on quick returns has been dominated by cross-sectional studies and a few longitudinal investigations. Although quick returns have consistently been associated with negative health and safety outcomes, it is unclear whether quick returns are the cause of these negative outcomes. This trial will thus be the first sincere attempt to establishing such a causal relationship.

There are several major strengths to this trial. The intervention is carried out in all eligible hospital units at Haukeland University Hospital, in which we retrieve objective register data (notably with no missing data) on the primary outcome measure – sickness absence. Hence reporting bias such as social desirability and memory biases will be avoided. This study is unique as it will imply complete access to the entire target population, also including individuals who typically choose not to participate in such studies. Hence this ensures full representativeness, strengthening the external validity of the study. Further, we have access to objective data on exposure to shift work (quick returns and other shift characteristics) during the intervention period. This provides us the opportunity to accurately assess compliance with the intervention and the true reduction in quick returns that occur, as well as monitoring other systemic differences that might occur in the shift schedule between the two parallel conditions. It is also an asset that we combine objective data with data collected via questionnaire. This provides us the opportunity to study the effect of abolishing or reducing quick returns on sleep, health and safety, as well as being able, for example, to study potential moderators to any effects we observe.

There are also some possible limitations with this trial that should be mentioned. The trial is conducted in a naturalistic setting which does not allow for the same strict control as generally would be preferred in experimental designs. One main concern is how well the intervention

For peer review only - http://bmjopen.bmj.com/site/about/guidelines.xhtml

group will succeed in abolishing quick returns from the shift schedule. We expect that for many individuals it will be a matter of reducing the number of quick returns, rather than complete abolition, for example, since such shift transitions occasionally may be necessary to ensure adequate staffing. Another concern is that a shift schedule that does not include quick returns may unintentionally include other unfavourable shift characteristics that could potentially confound the results. However, during the implementation of the trial, shift planners are provided with recommendations on how to set up shift schedules without quick returns, e.g. avoiding backward shift rotations, which as far as possible avoids other unfavourable shift characteristics. Further, for the participants in this trial it will be obvious which study condition they have been allocated to, thus their expectations can potentially have an impact on results based on self-reported data.[37] A questionnaire was used to measure most secondary outcome variables in this trial. An important limitation with such subjective reports is possible bias related to the validity of the instruments and recall bias.[38] However, most of the variables were based on standardized questionnaires with adequate psychometric properties. Furthermore, most variables are subjective by their very nature and need accordingly to be measured with self-reports.

If a shift schedule without quick returns is shown to be associated with less sickness absence or positive effects on other outcomes compared to a control group, this may encourage a stricter compliance with the workers' right to have at least 11 hours off between two subsequent shifts. The results of this trial will provide valuable information to stakeholders (nurses responsible for developing shift schedules, trade unions, politicians, and innovators) about the effect of quick returns and individual tolerance to quick returns.

For peer review only

Author statement: AH, ØV, SP, BB, SW, SAL, ES, and MBN conceived the study. ØV and ILRD produced the first draft of the manuscript. All authors assisted in drafting of the final, submitted version of manuscript and all authors have approved this version.

Conflict of Interest: The authors declare that they have no conflict of interest.

Funding: The study was funded from The Research Council of Norway (303671) and the University of Bergen, Bergen, Norway. The Research Council of Norway, Drammensveien 288, 0283 Oslo, Norway, Telephone: +47 22 03 70 00, E-mail: post@forskningsradet.no. University of Bergen, P.O.Box 7800, 5020 Bergen, Norway, Telephone: +47 55 58 80 81, E-mail: post@uib.no. The sponsors had no role in a study design, collection, management, analysis, and interpretation of data; writing of the report; and the decision to submit the report for publication, including whether they will have ultimate authority over any of these activities. The sponsors had no authority over any of the above activities.

Data statement: De-identified data that underlie the results reported from the trial described in this protocol will be available to researchers from accredited research institutions. Access to data will be limited to investigators who provide a methodologically sound proposal and will be limited to a specified time period (commencing about 3 months after publication of a respective Article and ending after 5 years). To ensure compliance with the General Data Protection Regulation, data processing must be covered by the European Commission's standard contractual clauses for the transfer of data, which must be signed by the data requesters. Proposals and requests for data access should be directed to the corresponding author of the respective Article. User-friendly output from the trial will be disseminated to stakeholder and other relevant organisations.

Acknowledgments: We would like to thank Ljiljana Djuric-Rakovic and John Olav Larssen at Haukeland University Hospital for their invaluable help in setting up and distributing the electronic questionnaires for this study. We would also like to thank Helga Berdal Lorentzen and Ole-Daniel Tuft Virkesdal at the HR department at Haukeland University Hospital, and employee representatives of the Norwegian Nurses Organisation, Trade Union Delta, the joint organization for Child Welfare Educators, Social Workers and Learning Disability Nurse and others trade unions for their support and contribution in the implementation of this research project. We would also like to thank Lukas Krondorf at Vital Things AS for technical support during the registration of nurses' sleep using radar technology.

For peer review only

References

1. EU Directive. Directive 2003/88/EC of the European Parliament and of the Council of 4 November 2003 concerning certain aspects of the organisation of working time. In: European Parliament, ed., 2003.

2. Vedaa Ø, Harris A, Bjorvatn B, et al. Systematic review of the relationship between quick returns in rotating shift work and health-related outcomes. *Ergonomics* 2016;59:1-14.

3. European Foundation for the Improvement of Living and Working Conditions. Sixth European working conditions survey–overview report. Luxembourg: Publications Office of the European Union., 2016.

4. Nielsen HB, Hansen ÅM, Conway SH, et al. Short time between shifts and risk of injury among Danish hospital workers: a register-based cohort study. *Scandinavian Journal of Work, Environment & Health* 2018 doi: 10.5271/sjweh.3770

5. Vedaa Ø, Pallesen S, Waage S, et al. Short rest between shift intervals increases the risk of sick leave: a prospective registry study. *Occupational and Environmental Medicine* 2017;74:496-501.

6. Vedaa Ø, Mørland E, Larsen M, et al. Sleep detriments associated with quick returns in rotating shift work: A diary study. *Journal of Occupational and Environmental Medicine* 2017;59:522-27.

7. Nicoletti C, Spengler CM, Läubli T. Physical workload, trapezius muscle activity, and neck pain in nurses' night and day shifts: a physiological evaluation. *Applied ergonomics* 2014;45:741-46.

8. Trinkoff AM, Le R, Geiger-Brown J, et al. Work schedule, needle use, and needlestick injuries among registered nurses. *Infection Control and Hospital Epidemiology* 2007;28:156-64. doi: 10.1086/510785

9. Vedaa Ø, Harris A, Erevik E, et al. Short rest between shifts (quick returns) and night work are associated with work related accidents. *International Archives of Occupational and Environmental Health* 2019;92:829-35. doi: doi.org/10.1007/s00420-019-01421-8

For peer review only

10. Vedaa Ø, Harris A, Waage S, et al. A longitudinal study on the association between quick returns and occupational accidents. *Scandinavian Journal of Work, Environment & Health* 2020 doi: 10.5271/sjweh.3906

11. Ropponen A, Koskinen A, Puttonen S, et al. Exposure to working-hour characteristics and short sickness absence in hospital workers: A case-crossover study using objective data. *Int J Nurs Stud* 2019;91:14-21.

12. Larsen AD, Ropponen A, Hansen J, et al. Working time characteristics and long-term sickness absence: a large register-based study of Danish and Finnish nurses. *Int J Nurs Stud* 2020 doi: https://doi.org/10.1016/j.ijnurstu.2020.103639

13. Toften S, Pallesen S, Hrozanova M, et al. Validation of sleep stage classification using non-contact radar technology and machine learning (Somnofy®). *Sleep Medicine* 2020;75:54-61.

14. Baruch Y, Holtom BC. Survey response rate levels and trends in organizational research. *Human Relations* 2008;61:1139-60.

15. Pallesen S, Bjorvatn B, Nordhus IH, et al. A new scale for measuring insomnia: the Bergen Insomnia Scale. *Percept Mot Skills* 2008;107:691-706.

16. American Academy of Sleep M. International classification of sleep disorders, 3rd ed. 3rd ed. ed: Darien, IL: American Academy of Sleep Medicine 2014.

17. Waage S, Moen BE, Pallesen S, et al. Shift work disorder among oil rig workers in the North Sea. *Sleep* 2009;32(4):558-65. [published Online First: 2009/05/06]

18. Åhsberg E. Dimensions of fatigue in different working populations. *Scandinavian Journal of Psychology* 2000;31:231-41.

19. Di Milia L, Smith PA, Folkard S. A validation of the revised circadian type inventory in a working sample. *Personality and Individual Differences* 2005;39:1293-305.

20. Adan A, Archer SN, Hidalgo MP, et al. Circadian Typology: A Comprehensive Review. *Chronobiology International, 2012, Vol29(9), p1153-1175* 2012;29(9):1153-75. doi: 10.3109/07420528.2012.719971

21. Horne JA, Ostberg O. A self-assessment questionnaire to determine morningness-eveningness in human circadian rhythms. *International journal of chronobiology* 1976;4(2):97-110. [published Online First: 1976/01/01]

22. Adan A, Almirall H. Horne & Östberg morningness-eveningness questionnaire: A reduced scale. *Personality and Individual Differences* 1991;12(3):241-53. doi: https://doi.org/10.1016/0191-8869(91)90110-W

23. Schmalbach B, Zenger M, Tibubos AN, et al. Psychometric properties of two brief versions of the Hopkins Symptom Checklist: HSCL-5 and HSCL-10. *Assessment* 2021;28:617-31.

24. Brayfield AH, Rothe HF. An index of job satisfaction. *Journal of applied psychology* 1951;35:307.

25. Kinnunen U, Feldt T, Geurts S, et al. Types of work-family interface: Well-being correlates of negative and positive spillover between work and family. *Scandinavian Journal of Psychology* 2006;47:149-62.

26. Vedaa O, Harris A, Erevik EK, et al. Short rest between shifts (quick returns) and night work is associated with work-related accidents. *Int Arch Occup Environ Health* 2019 doi: 10.1007/s00420-019-01421-8 [published Online First: 2019/03/18]

27. Singh J, Verbeke W, Rhoads GK. Do organizational practices matter in role stress processes? A study of direct and moderating effects for marketing-oriented boundary spanners. *Journal of Marketing* 1996;60:69-86. doi: 10.2307/1251842

28. Schaufeli WB, Salanova M, González-Romá V, et al. The measurement of engagement and burnout: a confirmative analytic approach. *Journal of Happiness Studies* 2002;3:71– 92.

29. Eriksen HR, Ihlebæk C, Ursin H. A scoring system for subjective health complaints (SHC). *Scandinavian Journal of Public Health* 1999;27:63-72.

30. Sonnentag S, Fritz C. The Recovery Experience Questionnaire: development and validation of a measure for assessing recuperation and unwinding from work. *Journal of Occupational Health Psychology* 2007;12:204-21.

31. Johns MW. A new method for measuring daytime sleepiness: the Epworth sleepiness scale. *Sleep* 1991;14:540-45.

For peer review only

32. Pallesen S, Grønli J, Myhre K, et al. A pilot study of impulse radio ultra wideband radar technology as a new tool for sleep assessment. *Journal of Clinical Sleep Medicine* 2018;14:1249-54.

33. StataCorp. Stata Statistical Software: Release 14. College Station, TX: StataCorp LP 2015.

34. Enders CK. Applied missing data analysis. New York, NY, US: Guilford Press 2010.

35. Hensing G, Alexanderson K, Allebeck P, et al. How to measure sickness absence? Literature review and suggestion of five basic measures. *Scandinavian Journal of Public Health* 1998;26:133-44.

36. Kraemer HC, Wilson GT, Fairburn CG, et al. Mediators and moderators of treatment effects in randomized clinical trials. *Archives of General Psychiatry* 2002;59:877-83.

37. Orne MT. On the social psychology of the psychological experiment: With particular reference to demand characteristics and their implications. *American Psychologist* 1962;17:776–83.

38. Weiss NS. Analytic approaches for dealing with possible recall bias in case-control studies—reply. *American Journal of Epidemiology* 1995;141:299.

For peer review only

Table 1. Examples of a two-week cycle of rotating shift work with and without quick returns

	Week 1							Week 2						
	Monday	Tuesday	Wednesday	Thursday	Friday	Saturday	Sunday	Monday	Tuesday	Wednesday	Thursday	Friday	Saturday	Sunday
Scenario1: Rotating three-shift **with** quick returns	Day	Day	Night	Night				Evening	Day	Day		Evening	Day	Evening
Scenario1: Rotating three-shift **without** quick returns	Day	Day	Night	Night				Day	Day	Day		Evening	Evening	Evening
Scenario2: Rotating three-shift **with** quick returns	Evening	Day	Day		Night	Night	Night			Evening	Day	Day		
Scenario2: Rotating three-shift **without** quick returns	Day	Evening	Evening		Night	Night	Night			Day	Day	Day		
Scenario3: Weekend shift **with** quick returns	Evening	Day	Day		Evening	Day	Evening	Day		Day	Day			
Scenario3: Weekend shift **without** quick returns	Day	Day	Day		Day	Evening	Evening		Day	Day	Evening			
Scenario4: Rotating two-shift **with** quick returns		Day	Day	Evening	Day			Evening	Day	Evening		Day		
Scenario4: Rotating two-shift **without** quick returns	Evening		Day	Day	Day			Evening	Evening		Day	Day		

Note. Rotating three-shift refers to a shift schedule in which the workers alternates between day-, evening- and night shifts. Rotating two-shift refers to a shift schedule in which the workers alternates between only two of the shifts (e.g., only working day and evening shifts).

For peer review only

Table 2. Key measures and timing of assessment

	Baseline	Six-month follow-up
**Primary outcome**		
From hospital register		
Sickness absence	X	X
**Secondary outcomes**		
Self-reported questionnaires		
The Bergen Insomnia Scale (BIS)	X	X
Shift work disorder (SWD)	X	X
The Swedish Occupational Fatigue Inventory (SOFI)	X	X
The revised Circadian Type Inventory (rCTI)	X	
The Horne-Östberg Morningness Eveningness Questionnaire (MEQ)	X	
The Hopkins Symptom Checklist - 5 (HSCL- 5)	X	X
Job Satisfaction Index (JSI)	X	X
The Work-Family Interface Scale (WFIS)	X	X
Work-related negative incidents	X	X
The Turnover Intention Scale (TIS)	X	X
The Utrecht Work Engagement Scale (UWES - 9)	X	X
Subjective Health Complaints inventory (SHC) (three of five subscales)	X	X
Recovery Experience Questionnaire (REQ) (two of four dimensions)	X	X
Epworth Sleepiness Scale (ESS)	X	X
Sleep monitoring study (≈50)		
Sleep diary (≥7 days)	X	X
Xethru sensor (≥7 days)	X	X
**Additional measures**		
Self-reported questionnaires		
Unwanted/negative effects		X
Self-rostering	X	X
Experience of the implementation of the intervention		X
Physical activity	X	X
Commute time	X	
Sleep duration and perceived need for sleep	X	X
Use of sleep medication and light treatment	X	X
Satisfaction with work schedule	X	X
Preferred presence of quick return in work schedule	X	X
**Demographics and background information**		
From hospital register		
Sex	X	
Age	X	
Percentage of full-time equivalent	X	X
Payroll data	X	X
Self-reported questionnaires		

For peer review only

Marital status	X
Highest completed degree	X
Years of experience with shift work	X
Children living at home	X

For peer review only

Figure caption

Figure 1. Flow Diagram of timeline for recruitment, randomization, assessments and for undertaking primary and secondary analyses

For peer review only

BMJ Open

Health promoting work schedules: protocol for a large-scale cluster randomized controlled trial on the effects of a work schedule without quick returns on sickness absence among healthcare workers

Journal:	*BMJ Open*
Manuscript ID	bmjopen-2021-058309.R2
Article Type:	Protocol
Date Submitted by the Author:	14-Feb-2022
Complete List of Authors:	Vedaa, Oystein; Norwegian Institute of Public Health, Department of Health Promotion; Norwegian University of Science and Technology, Department of Mental Health<br>Djupedal, Ingebjørg Louise Rockwell; University of Bergen, Department of Psychosocial Science<br>Svensen, Erling; Haukeland Universitetssjukehus<br>Waage, Siri; University of Bergen, Department of Global Public Health and Primary Care<br>Bjorvatn, Bjørn; Universitetet i Bergen Det medisinsk-odontologiske fakultet, Department of Global Public Health and Primary Care; Haukeland Universitetssjukehus, Norwegian Competence Center for Sleep Disorders<br>Pallesen, Ståle; University of Bergen<br>Lie, S; University of Bergen, Department of Dentistry<br>Nielsen, Morten; University of Bergen<br>Harris, Anette; Universitetet i Bergen Det Psykologiske Fakultet
**Primary Subject Heading**:	Occupational and environmental medicine
Secondary Subject Heading:	Public health
Keywords:	OCCUPATIONAL & INDUSTRIAL MEDICINE, Adult psychiatry < PSYCHIATRY, SLEEP MEDICINE

SCHOLARONE™
Manuscripts

Health promoting work schedules: protocol for a large-scale cluster randomized controlled trial on the effects of a work schedule without quick returns on sickness absence among healthcare workers

Øystein Vedaa[1,2,3], Ingebjørg Louise Rockwell Djupedal[4], Erling Svensen[5], Siri Waage[6,7], Bjørn Bjorvatn[6,7], Ståle Pallesen[4,7,8], Stein Atle Lie[9], Morten Birkeland Nielsen[4,10], Anette Harris[4]

[1] Department of Health Promotion, Norwegian Institute of Public Health, Bergen, Norway;

[2] Department of Mental Health, Norwegian University of Science and Technology, Trondheim, Norway;

[3] Department of Research and Development, St Olavs University Hospital, Trondheim, Norway

[4] Department of Psychosocial Science, University of Bergen, Bergen, Norway;

[5] Haukeland University Hospital, Bergen, Norway;

[6] Department of Global Public Health and Primary Care, University of Bergen, Bergen, Norway;

[7] Norwegian Competence Center for Sleep Disorders, Haukeland University Hospital, Bergen, Norway;

[8] Optentia at the Vaal Triangle Campus of the North-West University, South-Africa;

[9] Department of Clinical Dentistry, University of Bergen, Bergen, Norway;

[10] National Institute of Occupational Health, Oslo, Norway

Correspondence to: Øystein Vedaa, Department of Health Promotion, Norwegian Institute of Public Health, Zander Kaaes gate 7, 5018 Bergen, Norway; e-mail: Oystein.Vedaa@fhi.no

For peer review only

Word count: 5962

Date and version identifier:

- Issue date: 13 Feb 2022

Protocol amendment number: 03

Abstract

Introduction In shift work, quick returns refer to transitions between two shifts with less than 11 hours available rest time. Twenty-three per cent of employees in European countries reported having quick returns. Quick returns are related to short sleep duration, fatigue, sleepiness, work-related accidents, and sickness absence. The present study is the first randomized controlled trial (RCT) to investigate the effect of a work schedule without quick returns for six months, compared to a work schedule that maintains quick returns during the same time frame.

Methods and analysis A parallel-group cluster RCT in a target sample of more than 4000 healthcare workers at Haukeland University Hospital in Norway will be conducted. More than 70 hospital units will be assessed for eligibility and randomized to a work schedule without quick returns for six months or continue with a schedule that maintains quick returns. The primary outcome is objective records of sickness absence; secondary outcomes are questionnaire data (n ≈ 4000 invited) on sleep and functioning, physical and psychological health, work-related accidents, and turnover intention. For a subsample, sleep diaries and objective sleep registrations with radar technology (n ≈ 50) will be collected.

Ethics and dissemination The study protocol was approved by the Regional Committee for Medical and Health Research Ethics in Western Norway (2020/200386). Findings from the trial will be disseminated in peer-reviewed journals and presented at national and international conferences. Exploratory analyses of potential mediators and moderators will be reported. User-friendly outputs will be disseminated to relevant stakeholders, unions and other relevant societal groups.

Trial registration number NCT04693182; Pre-recruitment.

Key words: Quick returns, Shift work, Sickness absence, Sick leave

For peer review only

Article summary:

Strengths and limitations of this study:

- This is a randomized controlled trial to investigate the effect of a work schedule without quick returns.
- The primary outcome measure is objective register data on sickness absence with no missing data.
- As this is an evaluation of an organizational quality improvement measure implemented for all employees at the hospital, we get to study the effect on the entire target population with full representativeness.
- One concern in this trial is how well the intervention group will succeed in abolishing quick returns from the shift schedule (given that this is a study conducted in a naturalistic setting).
- Another concern in this trial is that a shift schedule that does not include quick returns may unintentionally include other unfavourable shift characteristics that could potentially confound the results (e.g., more consecutive evening shifts).

For peer review only

INTRODUCTION

An important principle when planning shift schedules is that employees are apportioned sufficient time to rest and recover between shifts. According to the EU's Working Time Directive (2003/88 / EC),[1] employees are entitled to minimum 11 hours of rest between two consecutive shifts. Still, in some countries, including Norway, employers and the employees' representatives can agree on rest periods less than 11 hours between two shifts. In this realm, the term *quick return* refers to transitions between two shifts with less than 11 hours available rest time. Quick returns occur most often between an evening shift and a day shift the following day, but can also occur between a night shift and an evening shift, and between a day shift and a night shift the subsequent night.[2] In the sixth European Working Conditions Survey published in 2016,[3] 23 per cent of employees in European countries reported having at least one quick return during the last month. Quick returns seem to be particularly prevalent in the healthcare sector. In a large Danish register survey (n = 69,200), it was shown that, on average per year, 65 per cent of nurses, 38 per cent of physicians, and 26 per cent of medical secretaries had quick returns in their work schedule.[4]

Eleven hours define the upper limit of potential time for rest between two shifts in a quick return, while the actual time available is often substantially shorter. A Norwegian study investigating payroll data from nurses found that almost 2/3 of the quick returns involved rest time less than 9 hours between two shifts, and some employees (2%) even had rest time of less than 7 hours.[5] The time available for sleep and recuperation is further curtailed by the time it takes to commute to and from work, time for self-care, meals, family obligations and house chores. A systematic literature review reported that sleep duration in quick returns between evening and day shifts typically is reduced to 5-6.5 hours, compared to 7-8 hours on non-quick return nights.[2] In addition to reduced sleep duration, the most robust findings in the literature review were that quick returns were associated with more fatigue, higher levels of

For peer review only

sleepiness, and shift work disorder (i.e., sleep problems or sleepiness related to a recurring shift schedule). Individual studies also showed that quick returns were associated with poorer sleep quality, impaired general health and well-being, higher self-reported stress, and lower job satisfaction.[2]

The most immediate consequence of quick returns is probably shortened sleep.[6] It is reasonable to think that this in turn leads to a number of other negative consequences. In a diary study (sleep- and work-schedule), we found that nurses reported higher sleepiness during the day shift when they had quick return to the day shift, as compared to during other regular day shifts.[6] In fact, the results showed that the nurses were as sleepy during the day shift after a quick return as they were during night shifts. It is conceivable that high sleepiness represents a greater problem when it occurs during day shifts than during night shifts, since day shifts are often busier[7] and typically experienced as more stressful.[6] The combination of a high level of sleepiness during a stressful shift might represent a type of circumstance that increases the risk of accidents. Indeed, the association between quick returns and work-related accidents or injuries is established in previous research. In a large register-based study from Denmark, researchers linked payroll data of healthcare workers with national registers of injuries. The results showed that quick returns were associated with a 39 per cent higher risk of injury, compared with having 15-17 hours off between two shifts.[4] A longitudinal study found an increased risk of needlestick injuries among nurses who reported having quick returns as compared to nurses without quick returns.[8] A study based on cross-sectional data found that quick returns were associated with an increased risk of falling asleep at work, of experiencing work-related injuries to themselves, of injuring patients or others, and of damaging equipment at work.[9] In fact, the risk of experiencing injuries to themselves and damaging equipment at work was greater with quick returns than with night shifts. Another longitudinal study, partly based on the same data, demonstrated that nurses who experienced

For peer review only

an increase in the number of quick returns over time also had an increased risk of work-related accidents, whereas a decrease in the number of quick returns over time was associated with reduced risk of accidents.[10]

Over the past five years, researchers have increasingly begun to use register/payroll data on exposure to shift work when examining the consequences of different shift characteristics. These data are registered by the employees, typically at healthcare institutions and include information about the date and start and stop time for all shifts performed. In some cases, it is also possible to retrieve data on sickness absence from the same registers. These data comprise information on the date of each day of absence (self-certified and medically certified absence) due to illness. In a Finnish study using such register data from healthcare workers, the relationship between quick returns and short-term sick leave (1 to 3 days) was investigated. The results showed that having few quick returns (defined as 3 or fewer over a period of 28 days) was associated with a lower risk of short-term sick leave, while having many quick returns (5 or more over a period of 28 days) was associated with a higher risk of short-term sickness absence, compared to having no quick returns.[11] In a study based on Danish and Finnish register data, it was found that healthcare workers who had at least 13 quick returns during a year had a higher risk of long-term sick leave than those with fewer quick returns.[12] These findings are in line with results using corresponding register data in Norway.[5] In one study, the findings showed that exposure to quick returns one month was associated with a higher risk of sick leave the following month. On average, nurses had 3 quick returns per month, which corresponded to 21 per cent more sickness absence days the subsequent month (over and above the sickness absence days of workers without quick return).[5]

Research on quick return and health and safety related outcomes have so far all been based on correlational studies. We do not yet know whether these health outcomes are caused by

For peer review only

exposure to quick returns. The present study is the first randomized controlled trial (RCT) conducted to determine the effects of abolishing quick return from the work schedule.

Aims

This paper describes the protocol for a two-arm cluster randomized controlled trial that assesses the consequences of a shift work schedule abolishing quick returns, compared to a schedule maintaining quick returns for a six months period. First, we will examine any differential change in sickness absence (primary outcome) during the six-month intervention period. Second, we will examine if there are differential changes in sleep and functioning, physical and mental health, work-related accidents, and turnover intention, among others (secondary outcomes). Third, we will investigate if individual characteristics associated with shift work tolerance including sex, age, personality and subjectively reported sleep need moderate the negative effects of quick returns on the primary and secondary outcomes. Finally, the study will investigate if individual factors like satisfaction with work schedule, job satisfaction, job engagement and work-family interference moderate the negative effects of quick returns on the primary and secondary outcomes.

For peer review only

METHODS AND ANALYSIS

The protocol for the current trial follows the SPIRIT checklist for intervention trials. The trial is further pre-registered with the Clinical Trials website (ClinicalTrials.gov identified: NCT04693182).

Figure 1 shows the Flow Diagram for the current trial. The flow chart illustrates the timeline for recruitment, randomization, assessments and for undertaking primary and secondary analyses.

Insert Figure 1 about here

Research design

A cluster randomized controlled trial comparing a six months work schedule abolishing quick returns (intervention) with that of a six months work schedule maintaining a normal amount of quick returns (control) will be conducted. The clusters in this trial represent hospital units that are randomly selected to receive (or not receive) the intervention. 'Normal amount of quick returns' refer to that which is the common practice at the respective hospital unit in recent years (i.e., when no explicit changes have been made to the work schedule), which means that the total number of quick returns at the unit will vary from 329–2356 per year (on average, nurses have three quick returns per month at this hospital[5]). In September 2020, the hospital units were informed about the conditions they would be randomized to at the start of the study in 2021. Thus, the autumn of 2020 was spent planning the shift schedule for 2021 (i.e., removing quick returns for the intervention group and maintaining quick returns for the control group). Most hospital units started the intervention period in the first half of 2021,

For peer review only

while some units started the intervention period in the second half of 2021.The intervention period in this study is six calendar months.

The primary outcome is sickness absence retrieved from the local registers kept by the hospital (including short- and long-term sick leave). The baseline measurements will be sickness absence from the year preceding the intervention, which for each individual participant will be matched on duration and season to that of the intervention period. We will apply for ethical approval to use the register data from all employees at the randomised hospital units without obtaining individual consent. In addition, a consent-based part of the trial will be conducted, in which secondary outcome measures will be collected via questionnaire at baseline and six-month follow-up. All employees ($n \approx 4000$) at the randomized units will be asked to complete a digital questionnaire. This will be made available to the employees when they log on to enter their working hours ("MinGat"). Baseline assessment will occur prior to the intervention period, and follow-up assessment will occur towards the end of the intervention period. A subsample ($n \approx 50$) will be asked to record their sleep with advanced sleep radar technology (Somnofy™)[13] and subjectively with sleep diaries for ≥1 week at the baseline and follow-up assessments, respectively.

Participants and procedure

Recruitment

This trial is carried out in collaboration with the human resources department at Haukeland University Hospital, Bergen, Norway. All hospital care units that have 24-hour staffing at Haukeland University Hospital will be considered for inclusion in this trial. This will include all healthcare workers working shifts, except for physicians. Physicians are to be excluded since they often have a different shift schedule and compensation scheme compared to other occupational groups at the hospital. Hereinafter, 'all employees' refer to all healthcare workers

For peer review only

engaged in shift work at the randomised hospital units, except for physicians. All employees (n ≈ 4000) at the randomized hospital units will be asked to complete a questionnaire prior to, and at the end of, the intervention period. Recruitment for this part of the trial will take place via the hospital's internal website or through the site in which the employees enter their working hours ("MinGat"). Researchers (the authors of this paper) and human resources personnel at the hospital will attend staff meetings at all included units to inform about the research project and encourage participation. A subsample of n ≈ 50 employees (evenly distributed from the intervention and the control units) will be recruited by convenience for the objective sleep monitoring section of the trial.

Eligibility

The unit-level inclusion criteria are that the units should have 1) healthcare workers (other than physicians) who work rotating shifts, 2) employees who regularly have quick returns in their work schedule, and 3) a new shift rotation year commencing from the first half of 2021 (which is the case for most units at the included hospitals). Exclusion criteria at the unit-level are 1) units recently (or will in the near future) went through other major organizational changes that may confound the results of the trial (this includes during the period from one year before the intervention starts until the intervention period is over), or 2) unit's manager or a substantial number of employees strongly oppose participation. Haukeland University Hospital had a total of 76 units which were considered for eligibility.

This trial consists of three different data collections with an expected dissimilar number of participants: A) a register study, i.e. the primary investigation, in which we expect no missing data, B) a questionnaire study, i.e. the secondary investigation, with an expected response rate of 40-50 per cent,[14] and C) the sleep monitoring study, i.e., secondary investigation, conducted on a subsample of ≈50 employees recruited by convenience. All employees from the randomised hospital units working ≥80 percent of full-time equivalent will participate in

For peer review only

the register-based study (investigation A) and the same group will be asked to participate in the questionnaire-based study (investigation B). Finally, participants in the sleep monitoring study (investigation C) will be recruited by convenience from the same sample of healthcare workers requiring that they are working ≥80 percent of full-time equivalent.

Randomisation and masking

The randomization in this trial occurred at the cluster level, in which hospital units constituted the clusters. Hospital units can vary in terms of how much staff they need over the 24-hour day, hence, the work schedule and the occurrence of, for example, quick returns and night shifts can vary across the units. Similar units were therefore grouped together based on the fact that they shared some attributes or characteristics. Then a stratified randomization was performed to the two study conditions in a 1: 1 ratio. One subgroup could, for example, consist of units with emergency functions, another with intensive care functions, one with mental health care, and one with maternity care, etc. In total we had 10 strata and the sizes of each stratum varied between 2 and 19 hospital units. The randomization list for each stratum was generated by the online randomization webpage, www.randomization.com, and the list for each stratum was saved.

It is not possible for participants to be blinded to the group to which they are assigned. However, statistical analyses will be done by a researcher who is masked to group allocation.

Intervention

The intervention entails implementing a shift schedule which abolishes quick returns for a six-month intervention period. The mean number of quick returns in the various hospital units in this trial varies from 3–32 per year. The intervention means that this number is abolished or reduced as much as possible. For practical reasons the intervention may be a matter of reducing rather than completely abolishing quick returns. This might be in the case of

For peer review only

ensuring adequate staffing (e.g., due to sickness absence), and since employees for various reasons may make short-notice shift swaps in which it is not possible to comply with the rule of avoiding quick returns. The human resources department at the hospital will assist shift planners in identifying appropriate shift schedules that do not include quick returns. **Table 1** shows some of the examples that were used to show shift planners how this could be done.

The control condition in this trial implies that employees maintain the same number of quick returns as in previous years for the six-month intervention period. It is important to note that hospital units in the control group are not expected to experience any increase in the number of quick returns.

Insert Table 1 about here

Assessments

All assessments/instruments in this trial are described below. **Table 2** provides an overview of the source and timing of the assessments. The primary outcome in this trial is sickness absence (number of days or spells). We will compare the sickness absence in intervention group with the control group during the intervention period, while adjusting for previous sick leave from the corresponding period the year preceding the intervention (matched on duration and season). Other measures included in this trial are secondary outcomes or outcomes used in exploratory or subsidiary analyses.

Demographics

Demographic information will be obtained both from the register at the hospital as well as from a questionnaire. Information on sex, age and percentage of full-time equivalent will be

For peer review only

available from the register data; while information on marital status, highest completed education/degree, years of experience with shift work, and if the participant has children living at home will be collected through the questionnaire.

Primary outcome

Sickness absence data will be retrieved from the local records kept by the hospital.[5] This record includes information about the date of any absence of the individual employee, implying that it includes information about both short- and long-term sickness absence. Further, these data include information on whether the absence is self-certified or whether it is certified by a physician, whether the absence is due to a sick child of whom the employee has childcare responsibility of, and whether the absence is due to COVID-19 related issues (e.g., quarantine).

Secondary outcomes

The Bergen Insomnia Scale (BIS)[15] will be used to measure sleep problems among participants. The scale originally comprised six items that assess symptoms of insomnia. An additional item will be included to the scale in which we will ask about the duration of any sleep problems. This makes it possible to define insomnia according to the diagnostic criteria in the International Classification of Sleep Disorders-Third Edition,[16] Diagnostic and Statistical Manual of Mental Disorders-Fifth Edition, and the International Classification of Diseases-11th Revision.

Shift work disorder (SWD) will be measured with three standardised questions.[17] SWD was evaluated with three questions based on the criteria from the third edition of the International Classification of Sleep Disorders (ICSD-3).[16] The questions were: a) Do you have a work schedule that sometimes overlap with the time you usually sleep?, b) if yes, does this cause insomnia and/or excessive sleepiness due to reduced amount of sleep?, c) if yes, has this

For peer review only

lasted for at least three months? Participants will be classified as having SWD when responding “yes” to all three questions.

The revised Swedish Occupational Fatigue Inventory (SOFI) will be used to measure lack of energy, physical exertion, physical discomfort, lack of motivation and sleepiness.[18] Participants are asked to indicate the extent to which they have recently (or for a specified period of time) experienced a list of 20 psychological and physical sensations related to fatigue.

The revised Circadian Type Inventory (rCTI) comprises 11 items, five of which assesses flexibility and six assesses languidity.[19] High scores on flexibility reflect better ability to sleep and work at odd times, whereas high scores on languidity indicate difficulties overcoming drowsiness and feelings of lethargy following sleep loss.

The Horne-Östberg Morningness Eveningness Questionnaire (MEQ) is the most widely used morningness-eveningness inventory,[20] and is designed to determine preferred timing of sleep and activities during the 24-hour day.[21] The MEQ reduced version (rMEQ) will be used in the present trial, which is comprised of five items from the original scale.[22]

Hopkins Symptoms Checklist - 5 (HSCL-5) will be used to measure general psychological distress.[23] HSCL-5 includes five questions about nervousness or inner turmoil, fear or feeling anxious, feeling hopeless about the future, depression or melancholy, worry or restlessness. An average score can be calculated across the five items with values that vary from 1 to 4, in which higher scores indicate a higher degree of psychological distress. The composite score is sometimes recoded into a two-part variable in which a score higher than 2.00 is defined as a high score.

Job Satisfaction Index (JSI) comprise five items measuring satisfaction with work (e.g., “I find real enjoyment in my work”).[24] Each item is answered on a 5-point Likert scale, ranging

For peer review only

from 1 (strongly disagree) to 5 (strongly agree). Higher scores reflect higher levels of overall job satisfaction.

The Work Family Interface Scale[25] will be used to evaluate the four types of work–family spillover. Consisting of 14 items, the scale was designed to measure both negative and positive work–to– family (NWFS and PWFS) and family–to–work spillover (NFWS and PFWS). The responses were graded by a frequency based on a 1–5 Likert scale, with alternatives ranging from never to very often.

Work-related negative incidents will be assessed using eight items measuring the number of self-reported work-related accidents, near accidents and dozing off at work or while driving to or from work. These questions have been developed in connection with the Norwegian Survey of Shift work, Sleep and Health among Nurses (SUSSH), and have been used in several previous publications.[26]

The Turnover Intention Scale (TIS) will be used to measure turnover intention, which is comprised of three items adapted from Michigan Organizational Assessment Questionnaire.[27] The three items are: “I will actively look for a new job in the next year,” “I often think about quitting,” and “I will probably look for a new job by the next year.” Responses were recorded on a 5-point Likert scale from 1 (strongly disagree) to 5 (strongly agree), yielding a score range of 3–15. A high score indicates a high degree of turnover intention.

The Utrecht Work Engagement Scale (UWES - 9) will be used to measure work engagement.[28] The UWES is originally comprised of 17 items rated on a 7-point scale ranging from “never” (0) to “always/every day” (6). The 9-item version of the UWES includes three items for each of the three factors; Vigor (e.g., “At my job, I feel strong and vigorous”), Dedication (e.g., “I am enthusiastic about my job”), and Absorption (e.g., “When I am

For peer review only

working, I forget everything else around me"). A higher score indicates more work engagement.

Subjective Health Complaints inventory (SHC)[29] consists of a list of 29 common health complaints that participants grade the intensity of which they experience each complaint on a four-point scale (0 = not at all; 1 = a little; 2 = some; 3 = severe). In this study, we include three of the five subscales; i.e. musculoskeletal complaints, pseudoneurological complaints, and gastrointestinal complaints.

Recovery Experience Questionnaire (REQ)[30] will be used to measure recovery experiences. REQ is originally a 16-item questionnaire with the four subscales psychological detachment, relaxation, mastery, and control. The present study includes the subscales of psychological detachment and relaxation. Each item is scored on a 5-point Likert scale ranging from 1 (strongly disagree) to 5 (strongly agree) of which a higher score indicates better detachment/relaxation.

Epworth Sleepiness Scale (ESS)[31] will be used to measure participants sleepiness. ESS is an eight-item questionnaire asking the participants how likely they are to doze off or fall asleep in different situations of everyday life including (e.g. while sitting and reading, watching TV, when sitting and talking to someone, etc.). For each item, participants report the chance of dozing as never (0), slight (1), moderate (2), or high (3) (total score range between 0 – 24). A higher score indicates higher level of sleepiness.

Additional measures of unwanted/negative effects and other exploratory analyses

Other factors that may have an impact on how the employees react to the intervention will also be investigated. The participants' attitudes to the intervention and the research project will be measured, in addition to how they experience the implementation of the intervention. A set of questions measuring possible negative or unwanted effects of the intervention will be

For peer review only

developed for the purpose of this trial. These questions will specifically ask if the changed work schedule has led to disturbed sleep, more stress, worry, depression, overall less time for recovery between work periods, problems in work-family balance, disrupted social relationships, poorer psychosocial climate at work, experience of reduced quality of care offered to patients, etc. For some employees, it is possible that a work schedule that does not allow for quick returns represents a restricted opportunity to co-design their schedule (i.e., self-rostering) and reduces the duration of free periods. Therefore, we will measure the participants' perceived change in relation to these parameters. Furthermore, we will include questions about satisfaction with work schedule, commute time, habitual and preferred sleep duration, current use of prescribed or over-the-counter sleep medication, current use of light treatment to improve sleep, and participants' physical activity level. Finally, the questionnaire will include an open text box in which participants can write freely, for example about anything they would like to convey related to the intervention (e.g. topics/themes they felt was inadequately addressed in the survey).

Sleep will be assessed more thoroughly for a subsample of ≈50 employees. The measures of sleep will include daily self-rating of sleep-wake patterns reported using the consensus sleep diary,[17] as well as sleep measured objectively using the Xethru sensor, a low-powered ultra-wideband radar.[32] The sleep registration will occur for ≥7 days at baseline and at six-month follow-up.

Sample size

In this trial, all available hospital units at Haukeland University Hospital with healthcare workers who work rotating shifts will be assessed for eligibility. This includes 76 units and 4260 healthcare workers. Based on previous published data[5] we have calculated that a total of 2028 participants is sufficient to reveal a difference in days of sick leave of 0.9 and 1.25 with an ICC of 0.1 and an average size of the units of 52 (calculation made in: StataCorp. 2015).[33]

For peer review only

Thus, with the planned recruitment strategy (i.e., invite >70 units and >4000 healthcare workers) we expect to exceed this number and be well within the number of participants required for the primary outcome variable.

Data analysis plan

All analyses will be conducted based on the intention-to-treat population, unless otherwise stated. To examine the effects of a shift schedule abated of quick returns on primary and secondary outcomes, the observed rates or scores will be analysed by means of latent growth models (or other equivalent models such as generalized linear mixed models). The observed rates or scores before and during the intervention period will be modelled by a random intercept and a fixed slope. The effect of the intervention will be estimated by using the group variable (intervention vs. control) as a predictor of the slope. Between-group effect sizes (Cohen's d) will be calculated by dividing the mean difference in estimated change in scores from baseline to the follow-up assessment by the pooled SD at baseline. Robust maximum likelihood will be used as the estimator, providing unbiased estimates under the assumption of data being missing at random,[34] which might be partly met through the inclusion of baseline scores to the model. The primary outcome measure in this trial is sickness absence data retrieved from the register at the hospital, in which we expect no missing data. However, it is reasonable to expect some missing data on the secondary outcome measures, as data are collected through questionnaire or via the sleep radar and sleep diary.

As some data for the follow-up questionnaire and sleep radar/diary assessment will be missing not at random, the robustness of the results under the missing-at-random assumption will be tested by sensitivity analyses in which the missing scores at follow-up will be replaced by baseline values for each respective individual. Since it is possible to imagine that some participants may experience worsening because of the intervention, we will consider carrying out more rigorous sensitivity analyses. For example, by replacing missing scores at the

For peer review only

follow-up assessment with baseline scores multiplied by a given factor (higher or lower than 1.00 depending on the direction that indicates a worsening) in the intervention group and by 1.00 in the control group. These sensitivity analyses will only be performed on selected variables depending on the focus in the respective article.

The intention-to-treat analyses may be accompanied by selected per-protocol analyses in which we, based on payroll data, define a group that has completely abolished or had a satisfactory reduction in the number of quick returns during the intervention period.

The primary outcome of sick leave will mainly be analysed in terms of the total number of sickness absence days and periods (spells) for a given period *before* compared to *during* the intervention period.[5] The models of sickness absence will take into account the zero inflation in this type of data. Other operationalisations of sickness absence might also be considered in accordance with recommendations in the literature.[35] For a further investigation of the sickness absence data, we will consider the use of more complex survival analyses (e.g., Cox proportional hazards model), and we will also consider modelling time to return to work (from sickness absence) and/or time before taking sickness absence according to group allocation.

Since the introduction of a work schedule without quick returns may entail an alternative schedule with an increase in other undesirable characteristics (e.g., more consecutive evening shifts), we will consider conducting analyses that adjust for such characteristics.

Mediator and moderator analyses will be performed for exploratory purposes, based on the basic principle for such analyses in randomised controlled trials as described by others (e.g., [36]). For example, some of the data collected on demographics, sleep-related personality traits (rCTI and MEQ), mental health, among others, can be used to examine factors that may moderate the impact of the intervention.

For peer review only

Stakeholder and public involvement

This trial is carried out in close collaboration with the HR department at Haukeland University Hospital. In addition, representatives from all relevant trade unions at the hospital will be involved in the planning and implementation of the research project. The findings of the trial will be disseminated via scholars in terms of scientific paper and conference presentations, and by stakeholder/union advocacy and other relevant public and community groups. Further, Haukeland University Hospital will arrange a conference for other relevant stakeholders, in which research results will be presented and the implications of the findings will be discussed.

Patient involvement

No patient involved.

Ethics and dissemination

The study protocol was approved by the Regional Committee for Medical and Health Research Ethics in Western Norway (2020/200386). In this trial, all employees at the included hospital units will be randomized to one of two conditions, and we will retrieve register data on working hours and sickness absence without collecting individual consent. This poses an ethical dilemma since all participation in research – especially when people are exposed to an intervention – should be consent-based. However, the intervention in this trial is to abolish or substantially reduce quick returns, and *not* to increase any exposure. This is thus considered not to represent a significant burden on the participants, as the presence of quick returns is already a violation of the Working Environment Act. In addition, we expect that the intervention primarily will have beneficial effects on employees' health and safety. Abolishing or reducing the number of quick returns is a quality improvement measure that the Health Trust wants to implement independently of the present research project. The fact that

For peer review only

the intervention is carried out as a research project is considered an advantage for the employees, as far as we are able to uncover any unintended negative effects of the intervention and further to be able to empirically document potential benefits on health and safety.

The result of this trial will potentially impact subsequent standards and practice when it comes to planning shift schedules and their compliance with the Working Environment Act. As vast number of employees might be affected by the trial results, it is equally important that the results are representative of the employees. We believe this justifies the use of the employees' register data without obtaining individual consent.

Participants will be required to provide informed consent before participating in the questionnaire and sleep diary/radar part of the trial (see supplementary files 1 and 2, respectively). The recruitment and consent process emphasizes that participation is voluntary and that participants can withdraw from this part of the trial at any time point without any consequences. Self-report data are recorded in electronic files that are encrypted and password protected. No identifying information will be stored alongside the self-report data. Furthermore, only researchers directly involved in data analysis will be granted supervised access to de-identified participant data.

Findings from this randomized controlled trial will be disseminated in peer-reviewed publications and as conference presentations. After the research project is completed, Haukeland University Hospital will arrange a conference for stakeholders where the results and experience from the research will be disseminated and discussed.

DISCUSSION

To the best of our knowledge, this is the first randomized controlled trial to investigate the effect of a work schedule abolishing quick returns. Previous research on quick returns has

For peer review only

been dominated by cross-sectional studies and a few longitudinal investigations. Although quick returns have consistently been associated with negative health and safety outcomes, it is unclear whether quick returns are the cause of these negative outcomes. This trial will thus be the first sincere attempt to establishing such a causal relationship.

There are several major strengths to this trial. The intervention is carried out in all eligible hospital units at Haukeland University Hospital, in which we retrieve objective register data (notably with no missing data) on the primary outcome measure – sickness absence. Hence reporting bias such as social desirability and memory biases will be avoided. This study is unique as it will imply complete access to the entire target population, also including individuals who typically choose not to participate in such studies. Hence this ensures full representativeness, strengthening the external validity of the study. Further, we have access to objective data on exposure to shift work (quick returns and other shift characteristics) during the intervention period. This provides us the opportunity to accurately assess compliance with the intervention and the true reduction in quick returns that occur, as well as monitoring other systemic differences that might occur in the shift schedule between the two parallel conditions. It is also an asset that we combine objective data with data collected via questionnaire. This provides us the opportunity to study the effect of abolishing or reducing quick returns on sleep, health and safety, as well as being able, for example, to study potential moderators to any effects we observe.

There are also some possible limitations with this trial that should be mentioned. The trial is conducted in a naturalistic setting which does not allow for the same strict control as generally would be preferred in experimental designs. One main concern is how well the intervention group will succeed in abolishing quick returns from the shift schedule. We expect that for many individuals it will be a matter of reducing the number of quick returns, rather than complete abolition, for example, since such shift transitions occasionally may be necessary to

For peer review only

ensure adequate staffing. Another concern is that a shift schedule that does not include quick returns may unintentionally include other unfavourable shift characteristics that could potentially confound the results. However, during the implementation of the trial, shift planners are provided with recommendations on how to set up shift schedules without quick returns, e.g. avoiding backward shift rotations, which as far as possible avoids other unfavourable shift characteristics. Further, for the participants in this trial it will be obvious which study condition they have been allocated to, thus their expectations can potentially have an impact on results based on self-reported data.[37] A questionnaire was used to measure most secondary outcome variables in this trial. An important limitation with such subjective reports is possible bias related to the validity of the instruments and recall bias.[38] However, most of the variables were based on standardized questionnaires with adequate psychometric properties. Furthermore, most variables are subjective by their very nature and need accordingly to be measured with self-reports.

If a shift schedule without quick returns is shown to be associated with less sickness absence or positive effects on other outcomes compared to a control group, this may encourage a stricter compliance with the workers' right to have at least 11 hours off between two subsequent shifts. The results of this trial will provide valuable information to stakeholders (nurses responsible for developing shift schedules, trade unions, politicians, and innovators) about the effect of quick returns and individual tolerance to quick returns.

For peer review only

Author statement: AH, ØV, SP, BB, SW, SAL, ES, and MBN conceived the study. ØV and ILRD produced the first draft of the manuscript. All authors assisted in drafting of the final, submitted version of manuscript and all authors have approved this version.

Conflict of Interest: The authors declare that they have no conflict of interest.

Funding: The study was funded from The Research Council of Norway (303671) and the University of Bergen, Bergen, Norway. The Research Council of Norway, Drammensveien 288, 0283 Oslo, Norway, Telephone: +47 22 03 70 00, E-mail: post@forskningsradet.no. University of Bergen, P.O.Box 7800, 5020 Bergen, Norway, Telephone: +47 55 58 80 81, E-mail: post@uib.no. The sponsors had no role in a study design, collection, management, analysis, and interpretation of data; writing of the report; and the decision to submit the report for publication, including whether they will have ultimate authority over any of these activities. The sponsors had no authority over any of the above activities.

Data availability statement: De-identified data that underlie the results reported from the trial described in this protocol will be available to researchers from accredited research institutions. Access to data will be limited to investigators who provide a methodologically sound proposal and will be limited to a specified time period (commencing about 3 months after publication of a respective Article and ending after 5 years). To ensure compliance with the General Data Protection Regulation, data processing must be covered by the European Commission's standard contractual clauses for the transfer of data, which must be signed by the data requesters. Proposals and requests for data access should be directed to the corresponding author of the respective Article. User-friendly output from the trial will be disseminated to stakeholder and other relevant organisations.

Acknowledgments: We would like to thank Ljiljana Djuric-Rakovic and John Olav Larssen at Haukeland University Hospital for their invaluable help in setting up and distributing the electronic questionnaires for this study. We would also like to thank Helga Berdal Lorentzen and Ole-Daniel Tuft Virkesdal at the HR department at Haukeland University Hospital, and employee representatives of the Norwegian Nurses Organisation, Trade Union Delta, the joint organization for Child Welfare Educators, Social Workers and Learning Disability Nurse and others trade unions for their support and contribution in the implementation of this research project. We would also like to thank Lukas Krondorf at Vital Things AS for technical support during the registration of nurses' sleep using radar technology.

For peer review only

References

1. EU Directive. Directive 2003/88/EC of the European Parliament and of the Council of 4 November 2003 concerning certain aspects of the organisation of working time. In: European Parliament, ed., 2003.

2. Vedaa Ø, Harris A, Bjorvatn B, et al. Systematic review of the relationship between quick returns in rotating shift work and health-related outcomes. *Ergonomics* 2016;59:1-14.

3. European Foundation for the Improvement of Living and Working Conditions. Sixth European working conditions survey–overview report. Luxembourg: Publications Office of the European Union., 2016.

4. Nielsen HB, Hansen ÅM, Conway SH, et al. Short time between shifts and risk of injury among Danish hospital workers: a register-based cohort study. *Scandinavian Journal of Work, Environment & Health* 2018 doi: 10.5271/sjweh.3770

5. Vedaa Ø, Pallesen S, Waage S, et al. Short rest between shift intervals increases the risk of sick leave: a prospective registry study. *Occupational and Environmental Medicine* 2017;74:496-501.

6. Vedaa Ø, Mørland E, Larsen M, et al. Sleep detriments associated with quick returns in rotating shift work: A diary study. *Journal of Occupational and Environmental Medicine* 2017;59:522-27.

7. Nicoletti C, Spengler CM, Läubli T. Physical workload, trapezius muscle activity, and neck pain in nurses' night and day shifts: a physiological evaluation. *Applied ergonomics* 2014;45:741-46.

8. Trinkoff AM, Le R, Geiger-Brown J, et al. Work schedule, needle use, and needlestick injuries among registered nurses. *Infection Control and Hospital Epidemiology* 2007;28:156-64. doi: 10.1086/510785

9. Vedaa Ø, Harris A, Erevik E, et al. Short rest between shifts (quick returns) and night work are associated with work related accidents. *International Archives of Occupational and Environmental Health* 2019;92:829-35. doi: doi.org/10.1007/s00420-019-01421-8

For peer review only

10. Vedaa Ø, Harris A, Waage S, et al. A longitudinal study on the association between quick returns and occupational accidents. *Scandinavian Journal of Work, Environment & Health* 2020 doi: 10.5271/sjweh.3906

11. Ropponen A, Koskinen A, Puttonen S, et al. Exposure to working-hour characteristics and short sickness absence in hospital workers: A case-crossover study using objective data. *Int J Nurs Stud* 2019;91:14-21.

12. Larsen AD, Ropponen A, Hansen J, et al. Working time characteristics and long-term sickness absence: a large register-based study of Danish and Finnish nurses. *Int J Nurs Stud* 2020 doi: https://doi.org/10.1016/j.ijnurstu.2020.103639

13. Toften S, Pallesen S, Hrozanova M, et al. Validation of sleep stage classification using non-contact radar technology and machine learning (Somnofy®). *Sleep Medicine* 2020;75:54-61.

14. Baruch Y, Holtom BC. Survey response rate levels and trends in organizational research. *Human Relations* 2008;61:1139-60.

15. Pallesen S, Bjorvatn B, Nordhus IH, et al. A new scale for measuring insomnia: the Bergen Insomnia Scale. *Percept Mot Skills* 2008;107:691-706.

16. American Academy of Sleep M. International classification of sleep disorders, 3rd ed. 3rd ed. ed: Darien, IL: American Academy of Sleep Medicine 2014.

17. Waage S, Moen BE, Pallesen S, et al. Shift work disorder among oil rig workers in the North Sea. *Sleep* 2009;32(4):558-65. [published Online First: 2009/05/06]

18. Åhsberg E. Dimensions of fatigue in different working populations. *Scandinavian Journal of Psychology* 2000;31:231-41.

19. Di Milia L, Smith PA, Folkard S. A validation of the revised circadian type inventory in a working sample. *Personality and Individual Differences* 2005;39:1293-305.

20. Adan A, Archer SN, Hidalgo MP, et al. Circadian Typology: A Comprehensive Review. *Chronobiology International, 2012, Vol29(9), p1153-1175* 2012;29(9):1153-75. doi: 10.3109/07420528.2012.719971

For peer review only

21. Horne JA, Ostberg O. A self-assessment questionnaire to determine morningness-eveningness in human circadian rhythms. *International journal of chronobiology* 1976;4(2):97-110. [published Online First: 1976/01/01]

22. Adan A, Almirall H. Horne & Östberg morningness-eveningness questionnaire: A reduced scale. *Personality and Individual Differences* 1991;12(3):241-53. doi: https://doi.org/10.1016/0191-8869(91)90110-W

23. Schmalbach B, Zenger M, Tibubos AN, et al. Psychometric properties of two brief versions of the Hopkins Symptom Checklist: HSCL-5 and HSCL-10. *Assessment* 2021;28:617-31.

24. Brayfield AH, Rothe HF. An index of job satisfaction. *Journal of applied psychology* 1951;35:307.

25. Kinnunen U, Feldt T, Geurts S, et al. Types of work-family interface: Well-being correlates of negative and positive spillover between work and family. *Scandinavian Journal of Psychology* 2006;47:149-62.

26. Vedaa O, Harris A, Erevik EK, et al. Short rest between shifts (quick returns) and night work is associated with work-related accidents. *Int Arch Occup Environ Health* 2019 doi: 10.1007/s00420-019-01421-8 [published Online First: 2019/03/18]

27. Singh J, Verbeke W, Rhoads GK. Do organizational practices matter in role stress processes? A study of direct and moderating effects for marketing-oriented boundary spanners. *Journal of Marketing* 1996;60:69-86. doi: 10.2307/1251842

28. Schaufeli WB, Salanova M, González-Romá V, et al. The measurement of engagement and burnout: a confirmative analytic approach. *Journal of Happiness Studies* 2002;3:71– 92.

29. Eriksen HR, Ihlebæk C, Ursin H. A scoring system for subjective health complaints (SHC). *Scandinavian Journal of Public Health* 1999;27:63-72.

30. Sonnentag S, Fritz C. The Recovery Experience Questionnaire: development and validation of a measure for assessing recuperation and unwinding from work. *Journal of Occupational Health Psychology* 2007;12:204-21.

31. Johns MW. A new method for measuring daytime sleepiness: the Epworth sleepiness scale. *Sleep* 1991;14:540-45.

For peer review only

32. Pallesen S, Grønli J, Myhre K, et al. A pilot study of impulse radio ultra wideband radar technology as a new tool for sleep assessment. *Journal of Clinical Sleep Medicine* 2018;14:1249-54.

33. StataCorp. Stata Statistical Software: Release 14. College Station, TX: StataCorp LP 2015.

34. Enders CK. Applied missing data analysis. New York, NY, US: Guilford Press 2010.

35. Hensing G, Alexanderson K, Allebeck P, et al. How to measure sickness absence? Literature review and suggestion of five basic measures. *Scandinavian Journal of Public Health* 1998;26:133-44.

36. Kraemer HC, Wilson GT, Fairburn CG, et al. Mediators and moderators of treatment effects in randomized clinical trials. *Archives of General Psychiatry* 2002;59:877-83.

37. Orne MT. On the social psychology of the psychological experiment: With particular reference to demand characteristics and their implications. *American Psychologist* 1962;17:776–83.

38. Weiss NS. Analytic approaches for dealing with possible recall bias in case-control studies—reply. *American Journal of Epidemiology* 1995;141:299.

For peer review only

Table 1. Examples of a two-week cycle of rotating shift work with and without quick returns

	Week 1							Week 2						
	Monday	Tuesday	Wednesday	Thursday	Friday	Saturday	Sunday	Monday	Tuesday	Wednesday	Thursday	Friday	Saturday	Sunday
Scenario1: Rotating three-shift **with** quick returns	Day	Day	Night	Night				Evening	Day	Day		Evening	Day	Evening
Scenario1: Rotating three-shift **without** quick returns	Day	Day	Night	Night				Day	Day	Day		Evening	Evening	Evening
Scenario2: Rotating three-shift **with** quick returns	Evening	Day	Day		Night	Night	Night			Evening	Day	Day		
Scenario2: Rotating three-shift **without** quick returns	Day	Evening	Evening		Night	Night	Night			Day	Day	Day		
Scenario3: Weekend shift **with** quick returns	Evening	Day	Day		Evening	Day	Evening	Day		Day	Day			
Scenario3: Weekend shift **without** quick returns	Day	Day	Day		Day	Evening	Evening		Day	Day	Evening			
Scenario4: Rotating two-shift **with** quick returns		Day	Day	Evening	Day			Evening	Day	Evening		Day		
Scenario4: Rotating two-shift **without** quick returns	Evening		Day	Day	Day			Evening	Evening		Day	Day		

Note. Rotating three-shift refers to a shift schedule in which the workers alternates between day-, evening- and night shifts. Rotating two-shift refers to a shift schedule in which the workers alternates between only two of the shifts (e.g., only working day and evening shifts).

For peer review only

For peer review only - http://bmjopen.bmj.com/site/about/guidelines.xhtml

Table 2. Key measures and timing of assessment

	Baseline	Six-month follow-up
**Primary outcome**		
From hospital register		
Sickness absence	X	X
**Secondary outcomes**		
Self-reported questionnaires		
The Bergen Insomnia Scale (BIS)	X	X
Shift work disorder (SWD)	X	X
The Swedish Occupational Fatigue Inventory (SOFI)	X	X
The revised Circadian Type Inventory (rCTI)	X	
The Horne-Östberg Morningness Eveningness Questionnaire (MEQ)	X	
The Hopkins Symptom Checklist - 5 (HSCL- 5)	X	X
Job Satisfaction Index (JSI)	X	X
The Work-Family Interface Scale (WFIS)	X	X
Work-related negative incidents	X	X
The Turnover Intention Scale (TIS)	X	X
The Utrecht Work Engagement Scale (UWES - 9)	X	X
Subjective Health Complaints inventory (SHC) (three of five subscales)	X	X
Recovery Experience Questionnaire (REQ) (two of four dimensions)	X	X
Epworth Sleepiness Scale (ESS)	X	X
Sleep monitoring study (≈50)		
Sleep diary (≥7 days)	X	X
Xethru sensor (≥7 days)	X	X
**Additional measures**		
Self-reported questionnaires		
Unwanted/negative effects		X
Self-rostering	X	X
Experience of the implementation of the intervention		X
Physical activity	X	X
Commute time	X	
Sleep duration and perceived need for sleep	X	X
Use of sleep medication and light treatment	X	X
Satisfaction with work schedule	X	X
Preferred presence of quick return in work schedule	X	X
**Demographics and background information**		
From hospital register		
Sex	X	
Age	X	
Percentage of full-time equivalent	X	X
Payroll data	X	X
Self-reported questionnaires		

For peer review only

Marital status	X
Highest completed degree	X
Years of experience with shift work	X
Children living at home	X

For peer review only

Figure caption

Figure 1. Flow Diagram of timeline for recruitment, randomization, assessments and for undertaking primary and secondary analyses

For peer review only

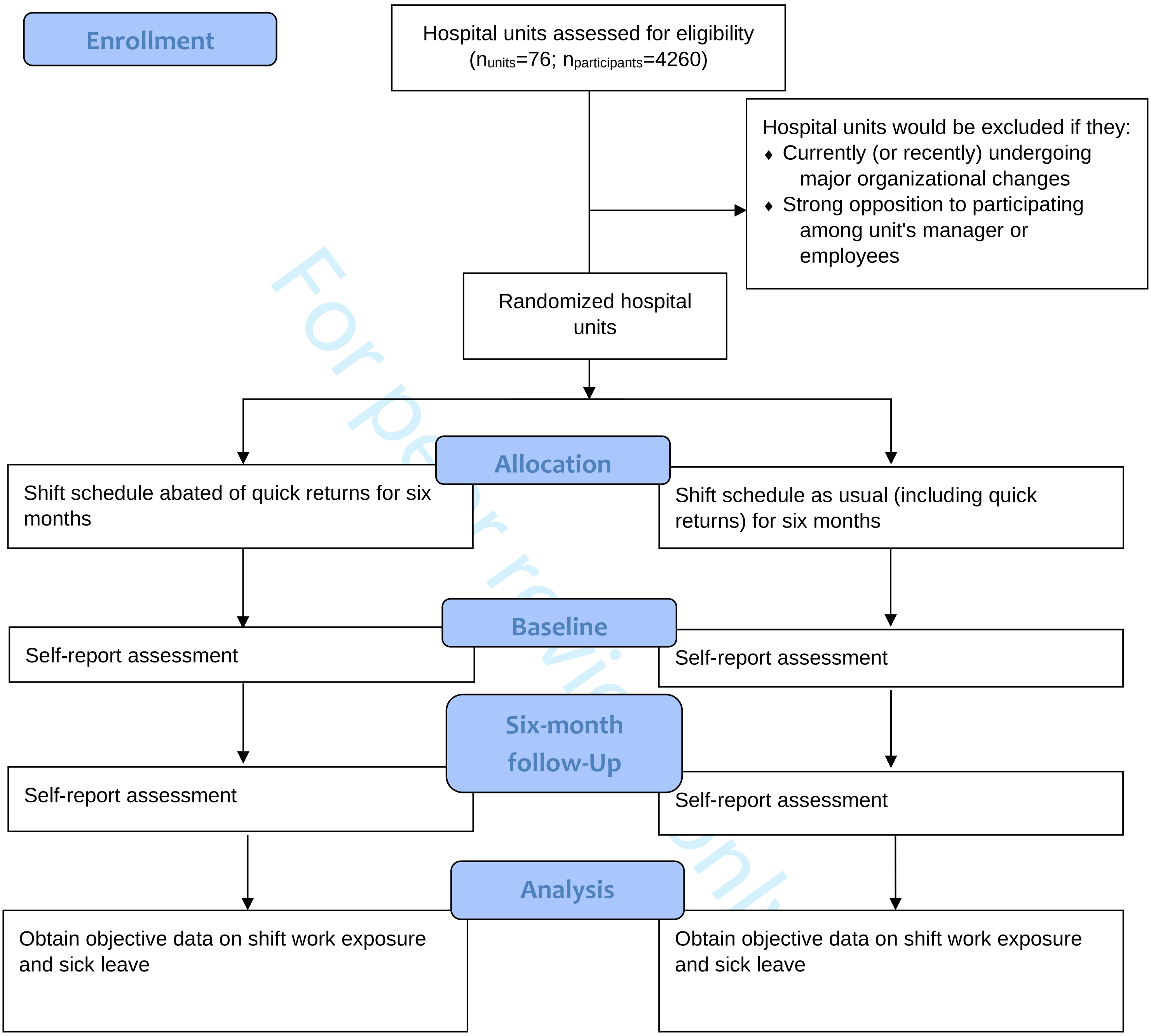

Figure 1. Flow Diagram of timeline for recruitment, randomization, assessments and for undertaking primary and secondary analyses

Supplementary file 1 INFORMASJONSSKRIV – versjon 20.12.2020

REQUEST FOR PARTICIPATION IN QUESTIONNAIRE

This is a request if you want to participate in the project on the effect of a shift schedule without quick returns - a randomized controlled trial among health workers who work shifts at Haukeland University Hospital. The main purpose of the survey is to test whether a shift schedule without quick return will reduce sickness absence among health personnel. In addition, it will be investigated whether such shifts lead to changes in sleep and functioning, including physical and mental health, work-related accidents and turnover intention. The study is conducted by researchers at the University of Bergen, the National Institute of Public Health and Haukeland University Hospital.

WHAT DOES THE PROJECT INVOLVE?

Participation means that you answer the questionnaire that is available when logging in to minGat. You will be asked to answer the questionnaire on two occasions. The first time is now, and then you get the questionnaire again in 6 months. The questionnaire contains questions about background (gender, age, marital status, responsibility for caring for children), work and health. Data will be linked to information about shifts (quick return shifts) and sick leave taken from the payroll register 12 months before and 12 months after the intervention. Data from the payroll register contains information about your work plan and about your sick leave. For your information, the hospital will investigate the connection between working hours and sick leave in the specified period, regardless of whether you agree to participate in the survey or not (cf. EU Privacy Regulation, Article 14). This data linkage is not consent-based as it is part of the hospital's quality improvement work.

POSSIBLE BENEFITS AND DISADVANTAGES

POSSIBLE BENEFITS: Some may find it educational and interesting to participate. By participating, you also get to contribute to research and to identify more health-promoting work schedules. You will get feedback on the results by through short articles published on “Innsiden”. The results can contribute to uncovering problematic conditions in healthcare workers' working conditions / situation.
POSSIBLE DISADVANTAGES: A possible disadvantage of participation may be that some may trigger some negative emotions if they have a problematic relationship with the topics we ask about.

VOLUNTARY PARTICIPATION AND OPPORTUNITY TO WITHDRAW YOUR CONSENT

It is voluntary to participate. As long as you can be identified in the data material, you have the right to: Access what personal information is registered about you, to have personal information about you corrected, to have personal information about you deleted, to receive a copy of your personal information, and to send a complaint to the Data Inspectorate about the processing of your personal data. In such cases, you can contact project manager Anette Harris (+47 55 58 32 19, anette.harris@uib.no). You can also contact the privacy ombudsman in Helse Bergen if you have questions about the health trust's processing of your personal information.

The legal basis for processing your personal data in the project is that the processing is necessary to perform a task in the public interest, and for quality improvement purposes (GDPR art. 6 (1) e) and art. 9 (2) i), and on the basis of your consent to voluntary participation in the project.

WHAT HAPPENS TO YOUR INFORMATION?

In this project, there are two types of information that are kept separate: 1) Data file with personally identifiable information (such as name, social security number and unique ID number) and 2) data file with the actual answers given and your unique ID number. The latter data is used for statistical analyzes. Only the researchers in the project

For peer review only - http://bmjopen.bmj.com/site/about/guidelines.xhtml

Supplementary file 1 INFORMASJONSSKRIV – versjon 20.12.2020

have access to these files. Data file with personally identifiable information and data file with your answers are stored separately on a secure server at UiB. All the researchers involved in the project have a statutory duty of confidentiality. When the project period is over, the file with all the personally identifiable information is deleted for good (no later than 01 January 2031). The answers you have given will then be deidentified.

APPROVAL

The Regional Committee for Medical and Health Research Ethics has made a research ethics assessment and approval of the project (2020/200386). The University of Bergen and project manager Anette Harris are responsible for the privacy of the project. An assessment of privacy conventions (DPIA) has been carried out in collaboration with the privacy ombudsman in Helse Bergen and at UiB.

CONTACT INFORMATION

If you have questions about the project, you can contact project manager Anette Harris (phone: 55 58 32 19; e-mail: anette.harris@uib.no) or work package manager Øystein Vedaa (phone: 21 07 88 34; email: oystein.vedaa@fhi.no). You can also contact us if you experience difficult feelings due to participating in the survey.
You can contact the University of Bergen's privacy representative if you have questions about the processing of your personal information in the project (Janecke Helene Veim, telephone: 55 58 20 29, email: janecke.veim@uib.no).

DO YOU WANT TO PARTICIPATE IN THIS RESEARCH PROJECT?

If you tick the box below, you give your consent to participate in this research project, and that data collected can be linked to information about your work schedule and sick leave retrieved from the payroll register, as described in this document. Tick the box below to give your consent:

☐ **I agree to participate in the research project and that data can be linked to my information from the payroll register**

For peer review only

Supplementary file 2

INFORMASJONSSKRIV – versjon 20.12.2020

REQUEST FOR PARTICIPATION IN QUESTIONNAIRE

This is a request if you want to participate in the project on the effect of a shift schedule without quick returns - a randomized controlled trial among health workers who work shifts at Haukeland University Hospital. The main purpose of the survey is to test whether a shift schedule without quick return will reduce sickness absence among health personnel. In addition, it will be investigated whether such shifts lead to changes in sleep and functioning, including physical and mental health, work-related accidents and turnover intention. The study is conducted by researchers at the University of Bergen, the National Institute of Public Health and Haukeland University Hospital.

WHAT DOES THE PROJECT INVOLVE?

Participation means that you keep a diary of your sleep and that you register your sleep with a sleep radar for 14 days, on two occasions. The first time is now, and then you will receive a sleep diary and sleep radar again in 6 months. The Sleep Diary contains 10 questions that you must answer every day upon awaking from a sleep period, for 14 days. The questions are about when you went to bed, how long it took you to fall asleep, and other questions related to your sleep and how you felt during the day. The sleep radar should be installed on your bedside table (or another table in the bedroom) and point towards your bed. It will collect information about your movement during the night using radar technology. Based on this, we can say something objective about how you sleep.

The data collected will be linked to your answers to the questionnaire you received in minGAT and information about shifts (quick returns) and sick leave retrieved from the payroll register 12 months before and 12 months after the intervention. Data from the payroll register contains information about your work plan and about your sick leave.

POSSIBLE BENEFITS AND DISADVANTAGES

POSSIBLE BENEFITS: Some may find it educational and interesting to participate. By participating, you also get to contribute to research and to identify more health-promoting work schedules. You will get feedback on the results by through short articles published on “Innsiden”. The results can contribute to uncovering problematic conditions in healthcare workers' working conditions / situation.
POSSIBLE DISADVANTAGES: A possible disadvantage of participation may be that some may trigger some negative emotions if they have a problematic relationship with the topics we ask about.

VOLUNTARY PARTICIPATION AND OPPORTUNITY TO WITHDRAW YOUR CONSENT

It is voluntary to participate. As long as you can be identified in the data material, you have the right to: Access what personal information is registered about you, to have personal information about you corrected, to have personal information about you deleted, to receive a copy of your personal information, and to send a complaint to the Data Inspectorate about the processing of your personal data. In such cases, you can contact project manager Anette Harris (+47 55 58 32 19, anette.harris@uib.no). You can also contact the privacy ombudsman in Helse Bergen if you have questions about the health trust's processing of your personal information.

The legal basis for processing your personal data in the project is that the processing is necessary to perform a task in the public interest, and for quality improvement purposes (GDPR art. 6 (1) e) and art. 9 (2) i), and on the basis of your consent to voluntary participation in the project.

WHAT HAPPENS TO YOUR INFORMATION?

In this project, there are two types of information that are kept separate: 1) Data file with personally identifiable information (such as name, social security number and unique ID number) and 2) data file with the actual answers

For peer review only

Supplementary file 2 INFORMASJONSSKRIV – versjon 20.12.2020

given and your unique ID number. The latter data is used for statistical analyzes. Only the researchers in the project have access to these files. Data file with personally identifiable information and data file with your answers are stored separately on a secure server at UiB. All the researchers involved in the project have a statutory duty of confidentiality. When the project period is over, the file with all the personally identifiable information is deleted for good (no later than 01 January 2031). The answers you have given will then be deidentified.

APPROVAL

The Regional Committee for Medical and Health Research Ethics has made a research ethics assessment and approval of the project (2020/200386). The University of Bergen and project manager Anette Harris are responsible for the privacy of the project. An assessment of privacy conventions (DPIA) has been carried out in collaboration with the privacy ombudsman in Helse Bergen and at UiB.

CONTACT INFORMATION

If you have questions about the project, you can contact project manager Anette Harris (phone: 55 58 32 19; e-mail: anette.harris@uib.no) or work package manager Øystein Vedaa (phone: 21 07 88 34; email: oystein.vedaa@fhi.no). You can also contact us if you experience difficult feelings due to participating in the survey.
You can contact the University of Bergen's privacy representative if you have questions about the processing of your personal information in the project (Janecke Helene Veim, telephone: 55 58 20 29, email: janecke.veim@uib.no).

DO YOU WANT TO PARTICIPATE IN THIS RESEARCH PROJECT?

If you tick the box below, you give your consent to participate in this research project, and that data collected can be linked to information about your work schedule and sick leave retrieved from the payroll register, as described in this document. Tick the box below to give your consent:

☐ **I agree to participate in the research project and that data can be linked to my information from the payroll register**

For peer review only

STANDARD PROTOCOL ITEMS: RECOMMENDATIONS FOR INTERVENTIONAL TRIALS

SPIRIT 2013 Checklist: Recommended items to address in a clinical trial protocol and related documents*

Section/item	ItemNo	Description	Page or section on which item is reported
**Administrative information**			
Title	1	Descriptive title identifying the study design, population, interventions, and, if applicable, trial acronym	1 (front/cover page)
Trial registration	2a	Trial identifier and registry name. If not yet registered, name of intended registry	2 and 8
	2b	All items from the World Health Organization Trial Registration Data Set	Included in a separate document with the submission
Protocol version	3	Date and version identifier	1 (front/cover page)
Funding	4	Sources and types of financial, material, and other support	24
Roles and responsibilities	5a	Names, affiliations, and roles of protocol contributors	1 (front/cover page) and Author statement on page 24
	5b	Name and contact information for the trial sponsor	24

For peer review only

	5c	Role of study sponsor and funders, if any, in study design; collection, management, analysis, and interpretation of data; writing of the report; and the decision to submit the report for publication, including whether they will have ultimate authority over any of these activities	24
	5d	Composition, roles, and responsibilities of the coordinating centre, steering committee, endpoint adjudication committee, data management team, and other individuals or groups overseeing the trial, if applicable (see Item 21a for data monitoring committee)	NA
**Introduction**			
Background and rationale	6a	Description of research question and justification for undertaking the trial, including summary of relevant studies (published and unpublished) examining benefits and harms for each intervention	See introduction from page 4
	6b	Explanation for choice of comparators	See Methods from page 8
Objectives	7	Specific objectives or hypotheses	See aims on page 7
Trial design	8	Description of trial design including type of trial (eg, parallel group, crossover, factorial, single group), allocation ratio, and framework (eg, superiority, equivalence, noninferiority, exploratory)	See Methods from page 8
**Methods: Participants, interventions, and outcomes**			
Study setting	9	Description of study settings (eg, community clinic, academic hospital) and list of countries where data will be collected. Reference to where list of study sites can be obtained	See Methods from page 8

For peer review only

Eligibility criteria	10	Inclusion and exclusion criteria for participants. If applicable, eligibility criteria for study centres and individuals who will perform the interventions (eg, surgeons, psychotherapists)	See Participants and procedure from page 9
Interventions	11a	Interventions for each group with sufficient detail to allow replication, including how and when they will be administered	See Intervention from page 11
	11b	Criteria for discontinuing or modifying allocated interventions for a given trial participant (eg, drug dose change in response to harms, participant request, or improving/worsening disease)	NA
	11c	Strategies to improve adherence to intervention protocols, and any procedures for monitoring adherence (eg, drug tablet return, laboratory tests)	See Methods from page 8
	11d	Relevant concomitant care and interventions that are permitted or prohibited during the trial	NA
Outcomes	12	Primary, secondary, and other outcomes, including the specific measurement variable (eg, systolic blood pressure), analysis metric (eg, change from baseline, final value, time to event), method of aggregation (eg, median, proportion), and time point for each outcome. Explanation of the clinical relevance of chosen efficacy and harm outcomes is strongly recommended	See Assessments from page 12
Participant timeline	13	Time schedule of enrolment, interventions (including any run-ins and washouts), assessments, and visits for participants. A schematic diagram is highly recommended (see Figure)	See Methods from page 8 and Figure 1
Sample size	14	Estimated number of participants needed to achieve study objectives and how it was determined, including clinical and statistical assumptions supporting any sample size calculations	See Sample size from page 17
Recruitment	15	Strategies for achieving adequate participant enrolment to reach target sample size	See Methods from page 8

Methods: Assignment of interventions (for controlled trials)

Allocation:			
Sequence generation	16a	Method of generating the allocation sequence (eg, computer-generated random numbers), and list of any factors for stratification. To reduce predictability of a random sequence, details of any planned restriction (eg, blocking) should be provided in a separate document that is unavailable to those who enrol participants or assign interventions	See Methods from page 8
Allocation concealment mechanism	16b	Mechanism of implementing the allocation sequence (eg, central telephone; sequentially numbered, opaque, sealed envelopes), describing any steps to conceal the sequence until interventions are assigned	See Methods from page 8 and Randomisation from page 11
Implementation	16c	Who will generate the allocation sequence, who will enrol participants, and who will assign participants to interventions	See Methods from page 8 and Randomisation from page 11
Blinding (masking)	17a	Who will be blinded after assignment to interventions (eg, trial participants, care providers, outcome assessors, data analysts), and how	See Methods from page 8 and Randomisation and masking from page 11
	17b	If blinded, circumstances under which unblinding is permissible, and procedure for revealing a participant's allocated intervention during the trial	See Methods from page 8 and Randomisation and masking from page 11

Methods: Data collection, management, and analysis

For peer review only - http://bmjopen.bmj.com/site/about/guidelines.xhtml

Data collection methods	18a	Plans for assessment and collection of outcome, baseline, and other trial data, including any related processes to promote data quality (eg, duplicate measurements, training of assessors) and a description of study instruments (eg, questionnaires, laboratory tests) along with their reliability and validity, if known. Reference to where data collection forms can be found, if not in the protocol	See Methods from page 8
	18b	Plans to promote participant retention and complete follow-up, including list of any outcome data to be collected for participants who discontinue or deviate from intervention protocols	We use registry data for the primary outcome. See Methods from page 8
Data management	19	Plans for data entry, coding, security, and storage, including any related processes to promote data quality (eg, double data entry; range checks for data values). Reference to where details of data management procedures can be found, if not in the protocol	See Methods from page 8
Statistical methods	20a	Statistical methods for analysing primary and secondary outcomes. Reference to where other details of the statistical analysis plan can be found, if not in the protocol	See Data analysis plan from page 18
	20b	Methods for any additional analyses (eg, subgroup and adjusted analyses)	See Data analysis plan from page 18
	20c	Definition of analysis population relating to protocol non-adherence (eg, as randomised analysis), and any statistical methods to handle missing data (eg, multiple imputation)	See Data analysis plan from page 18
**Methods: Monitoring**			
Data monitoring	21a	Composition of data monitoring committee (DMC); summary of its role and reporting structure; statement of whether it is independent from the sponsor and competing interests; and reference to where further details about its charter can be found, if not in the protocol. Alternatively, an explanation of why a DMC is not needed	There is no data monitoring committee beyond the research group.

	21b	Description of any interim analyses and stopping guidelines, including who will have access to these interim results and make the final decision to terminate the trial	NA
Harms	22	Plans for collecting, assessing, reporting, and managing solicited and spontaneously reported adverse events and other unintended effects of trial interventions or trial conduct	See page 16, Additional measures of unwanted/negative effects
Auditing	23	Frequency and procedures for auditing trial conduct, if any, and whether the process will be independent from investigators and the sponsor	The shifts schedule is prepared in advance for the entire intervention period, and there will be no changes to the schedule during the intervention period, with the exception of necessary short-notice shift swaps, as described on page 12. This limits the need for auditing.
**Ethics and dissemination**			
Research ethics approval	24	Plans for seeking research ethics committee/institutional review board (REC/IRB) approval	See Ethics form page 20

For peer review only - http://bmjopen.bmj.com/site/about/guidelines.xhtml

Protocol amendments	25	Plans for communicating important protocol modifications (eg, changes to eligibility criteria, outcomes, analyses) to relevant parties (eg, investigators, REC/IRBs, trial participants, trial registries, journals, regulators)	NA
Consent or assent	26a	Who will obtain informed consent or assent from potential trial participants or authorised surrogates, and how (see Item 32)	See Ethics form page 20
	26b	Additional consent provisions for collection and use of participant data and biological specimens in ancillary studies, if applicable	NA
Confidentiality	27	How personal information about potential and enrolled participants will be collected, shared, and maintained in order to protect confidentiality before, during, and after the trial	See Ethics and dissemination from page 20
Declaration of interests	28	Financial and other competing interests for principal investigators for the overall trial and each study site	Page 24
Access to data	29	Statement of who will have access to the final trial dataset, and disclosure of contractual agreements that limit such access for investigators	See Data statement on page 24
Ancillary and post-trial care	30	Provisions, if any, for ancillary and post-trial care, and for compensation to those who suffer harm from trial participation	NA
Dissemination policy	31a	Plans for investigators and sponsor to communicate trial results to participants, healthcare professionals, the public, and other relevant groups (eg, via publication, reporting in results databases, or other data sharing arrangements), including any publication restrictions	See Stakeholder and public involvement and Ethics and dissemination from page 20

For peer review only - http://bmjopen.bmj.com/site/about/guidelines.xhtml

	31b	Authorship eligibility guidelines and any intended use of professional writers	The Vancouver recommendations apply to authorship
	31c	Plans, if any, for granting public access to the full protocol, participant-level dataset, and statistical code	See Data statement on page 24
**Appendices**			
Informed consent materials	32	Model consent form and other related documentation given to participants and authorised surrogates	The consent form used is based on a template from the Regional Committee for Medical and Health Research Ethics in Norway. We do not consider it necessary to publish this with the protocol paper.
Biological specimens	33	Plans for collection, laboratory evaluation, and storage of biological specimens for genetic or molecular analysis in the current trial and for future use in ancillary studies, if applicable	NA

*It is strongly recommended that this checklist be read in conjunction with the SPIRIT 2013 Explanation & Elaboration for important clarification on the items. Amendments to the protocol should be tracked and dated. The SPIRIT checklist is copyrighted by the SPIRIT Group under the Creative Commons "Attribution-NonCommercial-NoDerivs 3.0 Unported" license.

For peer review only

Items from the World Health Organization Trial Registration Data Set

Data category	Information
Primary registry and trial identifying number	ClinicalTrials.gov
	NCT04693182
Date of registration in primary registry	Des, 2020
Secondary identifying numbers	N/A
Source(s) of monetary or material support	The Research Council of Norway (303671) and the University of Bergen, Bergen, Norway
Primary sponsor	The Research Council of Norway (303671)
Secondary sponsor(s)	University of Bergen, Bergen, Norway
Contact for public queries	Øystein Vedaa, PhD [Oystein.Vedaa@fhi.no]
Contact for scientific queries	Øystein Vedaa, PhD [Oystein.Vedaa@fhi.no]
	Department of Health Promotion, Norwegian Institute of Public Health, Bergen, Norway
Public title	Health promoting work schedules: Protocol for a large-scale cluster randomized controlled trial on the effects of a work schedule without quick returns on sickness absence among healthcare workers
Scientific title	Health promoting work schedules: Protocol for a large-scale cluster randomized controlled trial on the effects of a work schedule without quick returns on sickness absence among healthcare workers
Countries of recruitment	Norway
Health condition(s) or problem(s) studied	Shift work, sickness absence, health and sleep
Intervention(s)	Active comparator: a work schedule without quick returns for six months
	Placebo comparator: a work schedule with quick returns for six months
Key inclusion and exclusion criteria	Ages eligible for study: ≥18 years
	Sexes eligible for study: both
	Accepts healthy volunteers: yes
	Inclusion criteria: the unit-level inclusion criteria are that the units should have 1) healthcare workers (other than physicians) who work rotating shifts, 2) employees who regularly have quick returns in their work schedule, and 3) a new shift rotation year commencing from the first half of 2021 (which is the case for most units at the included hospitals)
	Exclusion criteria: exclusion criteria at the unit-level are 1) units recently (or will in the near future) went through other major organizational changes that may confound the results of the trial (this includes during the period from one year before the intervention starts until the intervention period is over), or 2) unit's manager or a substantial number of employees strongly oppose participation

For peer review only

Study type	Interventional
	Allocation: cluster randomized intervention model. It is not possible to blind the intervention for the participants, but the statistician who carries out the analyzes will be blinded to group allocation.
	Primary purpose: prevention
Date of first enrolment	January 2021
Target sample size	3669
Recruitment status	Recruiting
Primary outcome(s)	Sickness absence data will be retrieved from the local records kept by the hospital.
Key secondary outcomes	Questionnaire data: Insomnia, Shift work disorder, Occupational Fatigue, Psychological distress, Job Satisfaction, Work–family spillover, Work-related negative incidents, Turnover Intention, Work Engagement, Subjective Health Complaints, Recovery Experience, Sleepiness, and Sleep

For peer review only
